# Dual α-amylase and α-glucosidase inhibition by 1,2,4-triazole derivatives for diabetes treatment

**DOI:** 10.1038/s41598-025-11214-4

**Published:** 2025-07-25

**Authors:** Mohammed A. Marzouk, Elsayed M. Mahmoud, Wesam S. Shehab, Sherif M. Fawzy, Samar M. Mohammed, Mahmoud Ashraf Abdel-Razek, Ghada E. Khedr, Doaa A. Elsayed

**Affiliations:** 1 Department of Pharmaceutical Organic Chemistry, Faculty of Pharmacy, Zagazig University, Zagazig, 44519 Egypt; 2 Department of Chemistry, Faculty of Science, Zagazig University, Zagazig, 44519 Egypt; 3 Department of Pharmaceutical Chemistry, Faculty of Pharmacy, Sinai University, Kantara Branch, Al-Ismailia, Egypt; 4 Microbiology and Immunology Department, Faculty of Pharmacy, Zagazig University, Zagazig, 44519 Egypt; 5 Department of Analysis and Evaluation, Egyptian Petroleum Research Institute, Cairo, 11727 Egypt

**Keywords:** 1,2,4-Triazole derivatives, Anti-diabetic activity, α-Amylase inhibitors, α-Glucosidase inhibitors, Molecular docking analysis, In vitro enzyme inhibition, Biochemistry, Drug discovery, Molecular biology, Biogeochemistry, Medical research

## Abstract

The development of effective antidiabetic agents remains a critical challenge in diabetes management. In this study, we introduce novel 1,2,4-triazole-based derivatives designed as dual inhibitors of α-amylase and α-glucosidase, key enzymes in carbohydrate metabolism. Molecular docking identified six promising candidates, with compounds 4 and 10 showing the highest potency. Both compounds exhibited strong α-glucosidase inhibition (IC_50_ = 0.27 ± 0.01 µg/mL and 0.31 ± 0.01 μg/mL, respectively), surpassing acarbose, and also demonstrated potent α-amylase inhibition (IC_50_ = 0.19 ± 0.01 μg/mL and 0.26 ± 0.01 μg/mL, respectively). Structure–activity relationship analysis highlighted the crucial role of acetyl and bromo substituents in enhancing enzyme inhibition. These findings position triazole-based scaffolds as promising candidates for the development of next-generation antidiabetic therapies.

**Supplementary Information:**

The online version contains supplementary material available at 10.1038/s41598-025-11214-4.

## Introduction

Diabetes mellitus is a widespread and increasingly prevalent metabolic disorder characterized by chronic hyperglycemia caused by insufficient insulin production, impaired insulin action, or both^1^. It has become a critical global health concern, with serious social and economic implications^2^. According to the International Diabetes Federation (IDF), an estimated 537 million adults worldwide were living with diabetes in 2021, and this number is projected to rise to 783 million by 2045^3^. The condition is associated with high rates of morbidity and mortality due to its chronic complications, including cardiovascular diseases, retinopathy, nephropathy, and neuropathy^4^. These complications arise from sustained hyperglycemia and oxidative stress, which cause progressive damage to blood vessels and organ systems over time.

Type 2 diabetes mellitus (T2DM) accounts for 90–95% of all diabetes cases and typically manifests in adults, although its incidence is rising among younger populations due to sedentary lifestyles and poor dietary habits^5^. Unlike type 1 diabetes, which is primarily autoimmune in origin and results from the destruction of pancreatic β-cells, T2DM is characterized by insulin resistance combined with a relative deficiency in insulin secretion^6^. The primary therapeutic goals in managing T2DM include maintaining optimal blood glucose levels, preventing the onset or progression of complications, and improving patient quality of life. While numerous pharmacological agents are available for this purpose, including insulin sensitizers, insulin secretagogues, and enzyme inhibitors, none are free from side effects or long-term safety concerns.

In recent years, there has been an increasing focus on postprandial glucose regulation as a key aspect of glycemic control^7^. Elevated postprandial blood glucose levels have been strongly associated with the development of diabetic complications, and clinical studies suggest that targeting postprandial hyperglycemia is more effective in reducing cardiovascular risk than focusing solely on fasting glucose levels^7^. One promising strategy in this context involves the inhibition of carbohydrate-hydrolyzing enzymes such as α-amylase and α-glucosidase, which play critical roles in the digestion of complex carbohydrates.

**Fig. 1:**
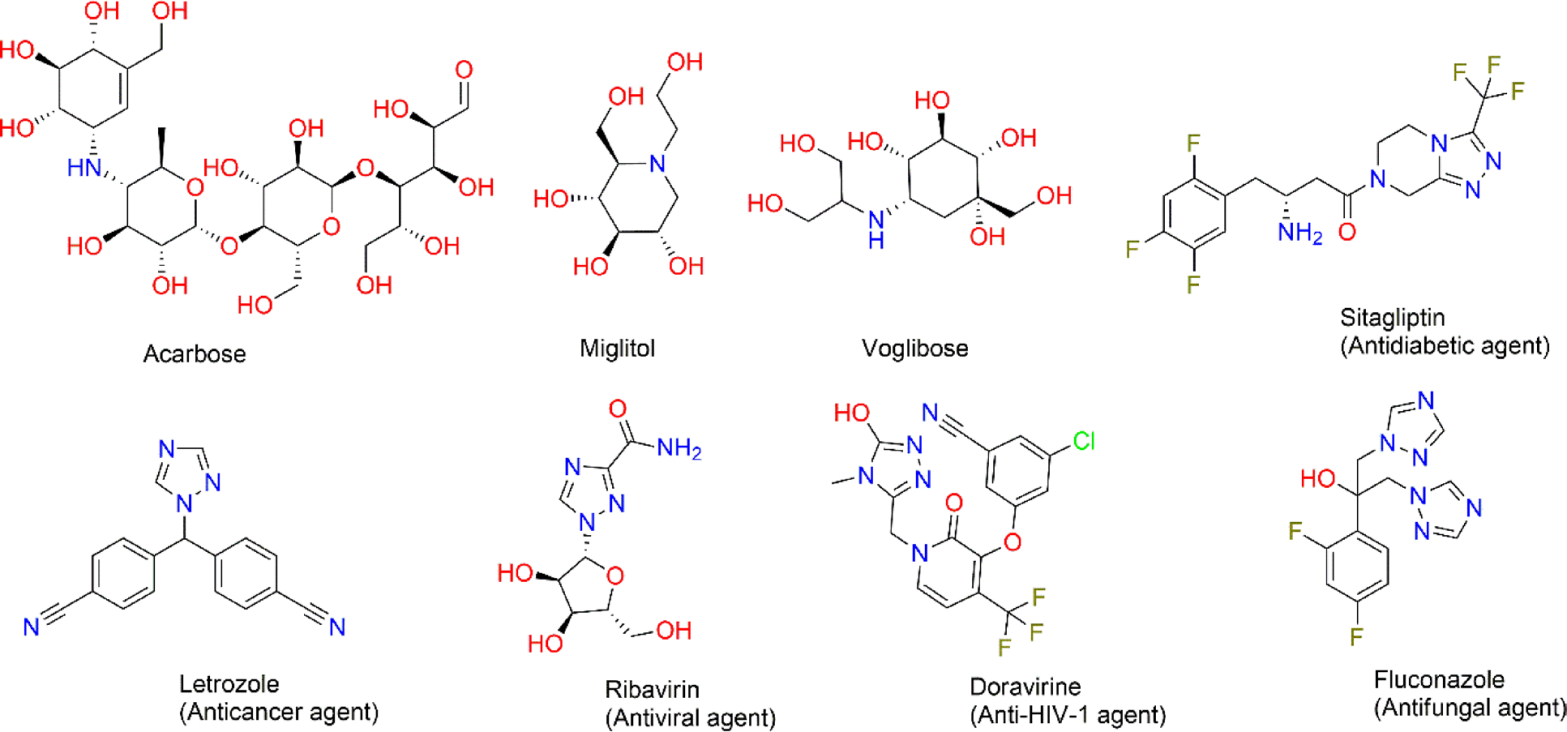
Acarbose, miglitol, voglibose, and some FDA approved bioactive agents containing 1,2,4-triazole ring structure.

α-Amylase is responsible for catalyzing the hydrolysis of α-1,4-glycosidic bonds in starch, producing maltose and oligosaccharides, which are then further broken down by α-glucosidase into glucose for absorption through the intestinal epithelium^8,9^. Inhibiting these enzymes can effectively reduce the rate of glucose release into the bloodstream, thereby preventing sharp post-meal spikes in blood sugar levels. Several drugs targeting these enzymes, including acarbose, miglitol, and voglibose, have been approved for clinical use and have shown efficacy in lowering postprandial glucose levels Fig. 1^10,11^. However, their application is often limited by adverse gastrointestinal effects such as flatulence, abdominal discomfort, and diarrhea, largely due to undigested carbohydrates undergoing fermentation by colonic bacteria^10,11^. These drawbacks underscore the urgent need to develop alternative enzyme inhibitors that retain efficacy while improving tolerability and patient adherence.

Among the diverse classes of heterocycles explored for antidiabetic drug discovery, the 1,2,4-triazole ring has gained considerable attention due to its versatile pharmacological profile and favorable physicochemical properties Fig. 1^12^. The 1,2,4-triazole nucleus is a five-membered ring containing three nitrogen atoms, which imparts strong hydrogen bonding potential, metal ion coordination capability, and metabolic stability. These characteristics make triazoles attractive candidates for drug design, particularly in enzyme inhibition^13^. Previous studies have reported the efficacy of 1,2,4-triazole derivatives in a range of therapeutic areas, including anticancer^13^, antimicrobial^14,15^, antiviral^16^, and anti-inflammatory^17^ applications. The versatility of the 1,2,4-triazole ring arises from its ability to form hydrogen bonds and coordinate with metal ions, enabling strong interactions with various biological targets^18,19^. Notably, their potential as antidiabetic agents has also been explored, with promising findings demonstrating that certain triazole-based molecules exhibit inhibitory activity against α-glucosidase^20,21^. Despite this progress, there remains a relative paucity of triazole derivatives designed specifically for dual inhibition of both α-amylase and α-glucosidase^22–24^, a strategy that could offer synergistic benefits in controlling postprandial hyperglycemia.

**Fig. 2:**
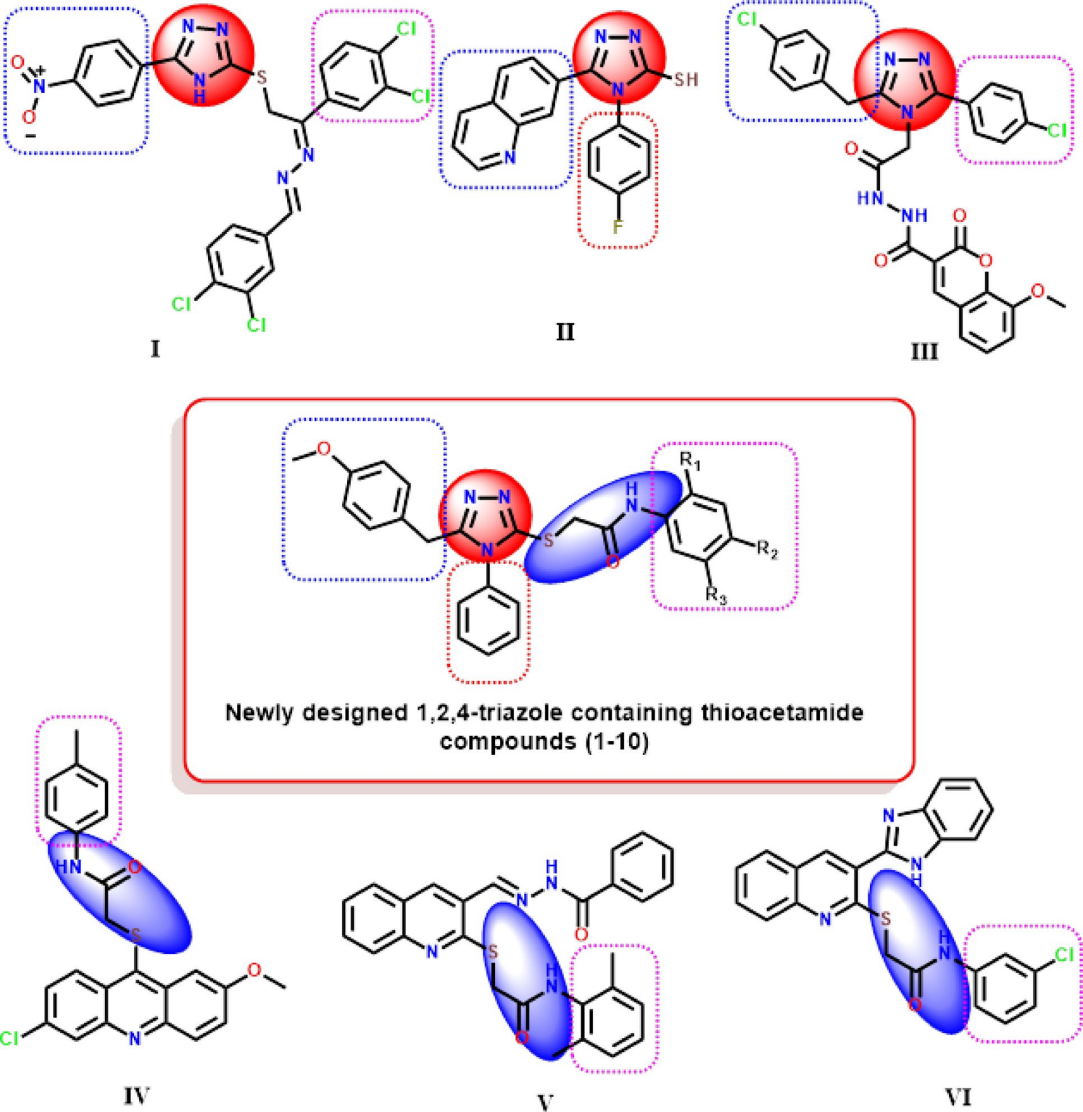
Structures of α-amylase and/or α-glucosidase inhibitors containing 1,2,4-triazole nucleus (**I**,** II**,** III**), α-glucosidase inhibitors containing thioacetamide linker (**IV**,** V**,** VI**) structures, and optimization strategy of designed molecules.

To address this therapeutic gap, we designed and synthesized a new class of 1,2,4-triazole-based compounds incorporating a thioacetamide bridge and *para*-substituted phenyl rings Fig. 2. This hybrid molecular architecture was chosen to synergistically enhance biological activity by combining the enzyme-binding potential of the triazole scaffold with the flexibility and hydrogen bonding capacity of the thioacetamide moiety^25–27^. The thioacetamide linker was strategically introduced to facilitate interactions with polar residues in the enzyme active sites, while allowing structural adaptability to accommodate diverse binding pockets.

**Fig. 3:**
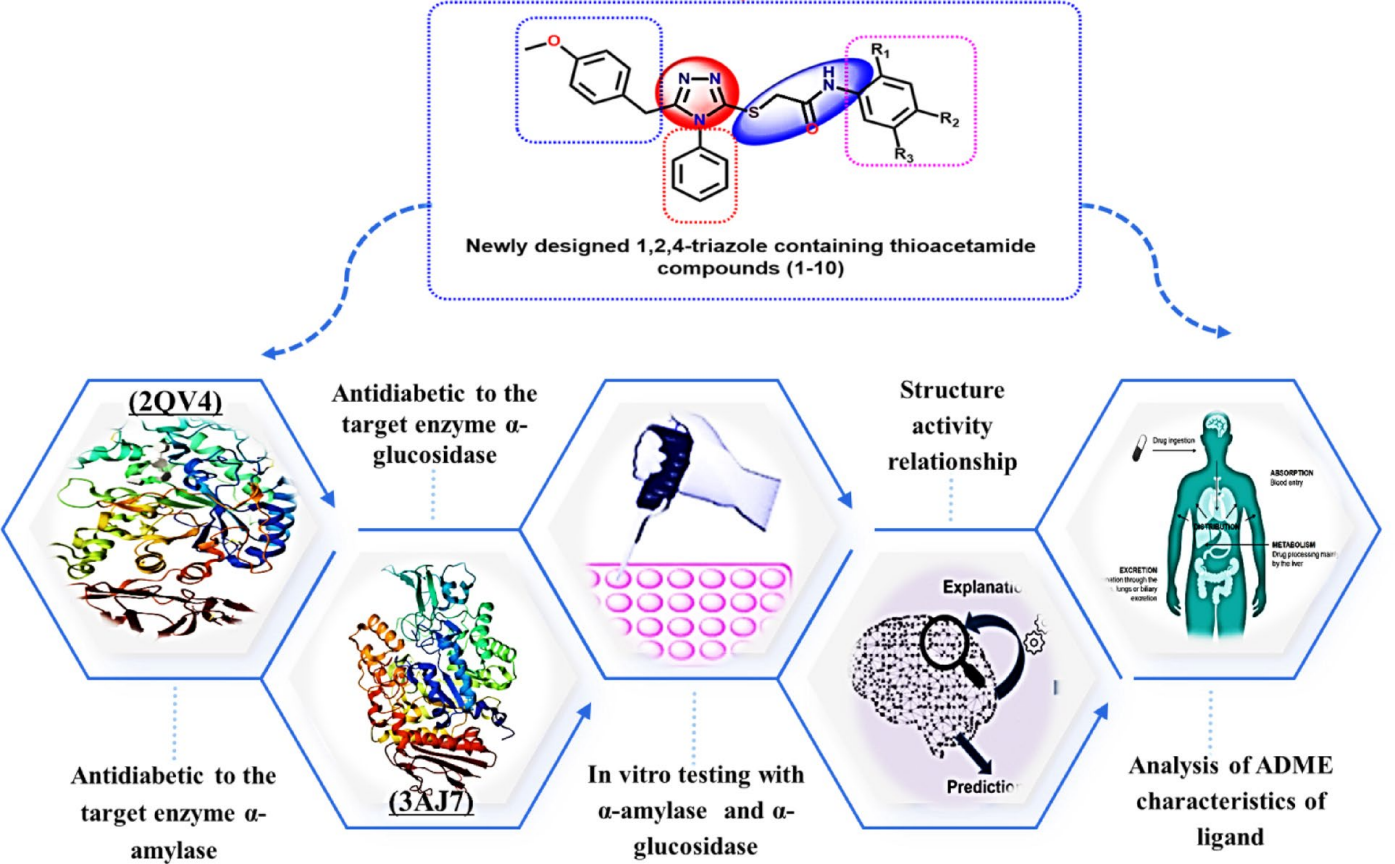
Rational of the work.

In the present study, our design strategy aimed to develop hybrid molecules bearing a 1,2,4-triazole nucleus connected via a thioacetamide bridge to various *para*-substituted phenyl rings Fig. 3. The substituents, including electron-withdrawing groups (such as acetyl and bromo) and electron-donating groups (such as methoxy), were chosen to modulate electronic distribution, hydrogen bonding capability, and lipophilicity^28^. These variations were expected to influence enzyme binding affinity and selectivity based on steric and electronic complementarity with the active sites of α-amylase and α-glucosidase^29,30^. Molecular modeling tools, including docking simulations and pharmacokinetic profiling, were employed to prioritize candidates for synthesis and biological evaluation. In particular, docking analyses guided the selection of substituents by predicting favorable interactions with key catalytic residues, such as TRP 59, ASP 197, and GLU 277, in the respective enzymes^25–27^. Additionally, SwissADME predictions ensured that lead compounds complied with key drug-likeness rules (e.g., Lipinski’s Rule of Five) and exhibited favorable absorption and solubility characteristics^31–34^.

Thus, the objective of this work was to design, synthesize, and characterize a new series of 1,2,4-triazole derivatives as potential dual inhibitors of α-amylase and α-glucosidase. Our approach integrates rational drug design, computational modeling, and biochemical validation to identify potent and selective antidiabetic agents. This study not only addresses the limitations of current enzyme inhibitors but also expands the structural diversity of small molecules available for the treatment of T2DM.

## Results and discussion

### Chemistry

**Scheme 1:**
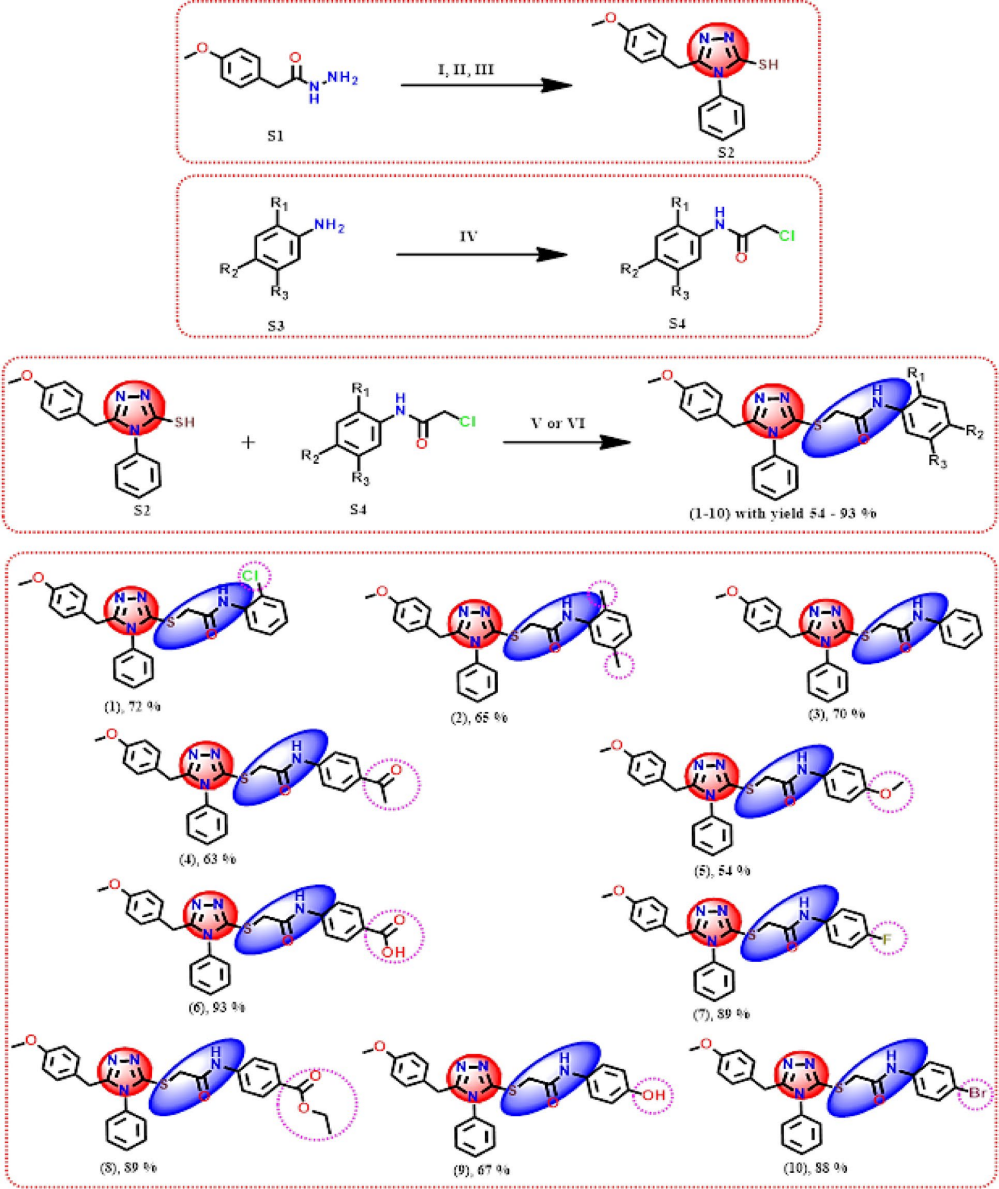
Pathway to the target 1,2,4-triazole new substances containing thioacetamide (1–10). Reagents and conditions: **(I)** Phenyl isothiocyanate, EtOH, reflux; **(II)** 10% NaOH, reflux; **(III)** HCl, r. t; **(IV)** 2-Chloroacetyl chloride, acetic acid, r. t; **(V)** Substituted 2-chloro-*N*-phenylacetamide, NaOH, EtOH, reflux for compounds **(1–7)** and **(9–10)**; **(VI)** Substituted 2-chloro-*N*-phenylacetamide, triethylamine, EtOH, r. t. for compound **8**.

The synthesis of the 1,2,4-triazole based thioacetamide target compounds is depicted in Scheme 1. The synthetic protocol depends on chemical modification of 2-(4-methoxyphenyl) acetohydrazide **S1** and aniline derivatives **S3** to 1,2,4-triazole-3-thiol derivative **S2** and substituted 2-chloro-*N*-phenylacetamides **S4**, respectively, followed by base catalyzed-alkylation of the thiol group of intermediate **S2** using the alkylating reagents **S4**. Initially, the intermediate, 5-(4-methoxybenzyl)-4-phenyl-4*H*-1,2,4-triazole-3-thiol **(S2)**, was synthesized from the starting material 2-(4-methoxyphenyl) acetohydrazide **S1** following previously reported procedures^35,36^. Next, the 2-chloro-*N*-phenylacetamide derivatives **S4** were synthesized from aniline derivatives **S3** using standard procedures^37,38^. Finally, the target 1,2,4-triazole compounds **1–10** were synthesized by alkylating the thiol group of intermediate **S2** with the alkylating reagents **S4** as a source of thioacetamide function. This reaction was carried out under basic conditions using sodium hydroxide in refluxing ethanol to afford the desired compounds **1–10** in moderate to high yield (54–93%), except for compound **8**, which was synthesized smoothly at room temperature using triethylamine as mild catalytic base with yield (89%). The reaction progress was monitored via TLC using EtOAc/Petroleum Ether (1:1) as the solvent system, which confirmed the complete conversion of the starting materials. The structures of the synthesized target compounds were confirmed by IR, ^1^H NMR and ^13^C NMR analyses. For instance, compound **4** showed three stretching bands appeared at 3230, 1680 and 1520 cm^−1^ for NH, C=O, and C=N functions, respectively. Additionally, the absence of the thiol stretching band indicating successful alkylation. It’s ^1^H NMR spectrum, including three singlet characteristic signals at δ = 3.67, 3.89 and 4.13 ppm assigned for (CH_3_) of the *p*-methoxy group and the two methylene protons of the alkylated side chain and thioacetamide linker, respectively. The aromatic protons resonated in the range 6.71–7.92 ppm, while, the singlet proton of the (NH) of the thioacetamide linker appeared at δ 10.65 ppm. Furthermore, the ^13^C NMR spectrum of compound **1** showed three signals at δ_c_ = 29.89, 36.47 and 55.01 ppm for sp^3^ carbons, whereas, the thiol-linked carbon appeared at δ_c_ 149.9 ppm and the amidic carbonyl group at δ_c_ 166.3 ppm. The structure of the new 1,2,4-triazoles **2**, **5** and **7** has been illustrated from their spectral data. For example, their IR spectra revealed the amidic (NH and C=O) and C=N functions at 3200–3229, 1641–1681, and 1490–1588 cm^−1^, respectively. The ^1^H NMR analysis of the triazole derivative **2** indicates the presence of two methyl, methoxy, two methylene, and amidic NH protons at δ_H_ 2.12, 2.22, 3.67, 3.91, 4.10 and 9.61 ppm as six singlets, respectively. The aryl protons appeared at δ_H_ 6.72, 6.80, 6.87, 7.06, 7.25 and (7.49–7.56) as five doublets and one multiplet. Its ^13^C NMR provided 22 signals at δ_C_ 17.31 (CH_3_), 20.71 (CH_3_), 29.88 (CH_2_), 36.40 (CH_2_-S), 54.98 (*O*-CH_3_), 113.7, 124.8, 125.8, 127.2, 127.5, 128.0, 129.4, 129.7, 129.9, 130.1, 132.8, 134.9, 135.7 (Ar–C), 150.0 (C-3), 154.8 (C-5), 157.9 (Ar–C) and 165.7 ppm (C=O) for sp^3^and sp^2^ carbons. The protons and carbons for the other compounds **5** and **7** are appeared at the expected field. Also, the chemical structure of compound **8** was elucidated from its spectral data. It was noted the presence of a broad band recorded in its IR spectrum at 1696 cm^−1^ that provided two (C=O) functions (ester and amide), beside amidic NH at 3220 cm^−1^. Furthermore, its ^1^H NMR assigned methoxy, two methylene, and NH-amide protons at δ_H_ 3.67, 3.89, 4.14 and 10.65 ppm as four independent singlets, respectively. While, the acetyl protons split as triplet and quartet at δ_H_ 1.30 and 4.27 ppm, respectively, with ^*3*^*J* = 6.8 Hz. Additionally, the aromatic protons are split at δ_H_ 6.71, 6.81, 7.25, 7.50, 7.67 and 7.91 ppm as six doublets. Another evidence for the synthesis of 1,2,4-triazole-based derivative **8** comes from its ^13^C NMR that demonstrated 21 signals at δ_C_ = 14.20 (CH_3_), 30.04 (CH_2_), 37.05 (CH_2_-S), 54.99 (*O*-CH_3_), 60.59 (*O*-CH_2_), for sp^3^ carbons and 113.7, 118.2, 123.9, 127.2, 127.5, 129.4, 129.7, 129.9, 130.3, 132.7, 143.0 (Ar–C), 149.8 (C-3), 154.9 (C-5), 157.9 (Ar–C), 165.2 (C=O, amide) and 166.2 ppm (C=O, ester) for sp^2^ carbons. Similar spectral patterns were observed for other derivatives, with variations depending on the substituents. The spectral data for all compounds corroborate their proposed structures and confirm the successful synthesis of the 1,2,4-triazole derivatives. These results validate the designed synthetic strategy and provide a solid foundation for further biological evaluation.

### Structure activity relationship

**Fig. 4:**
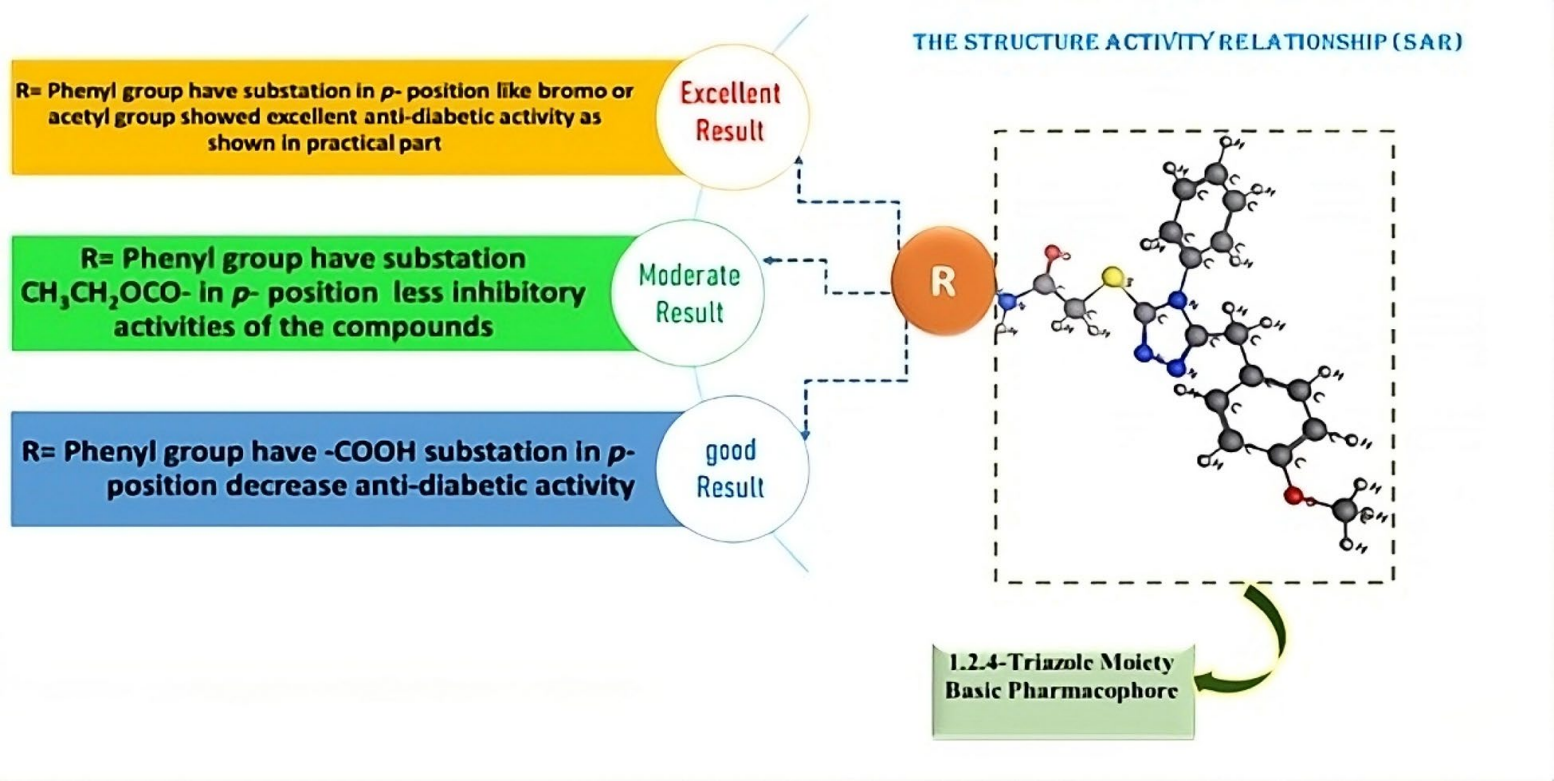
Summarized SAR of the produced 1,2,4-triazole containing thioacetamide.

The inhibitory activities of the 1,2,4-triazole derivatives (3–10) were rationalized through structural analysis, focusing on substituent effects at the *para*-position of the phenyl ring Fig. 4. Key observations include:

1. **Electron-Withdrawing Groups (EWGs)**:

- **Acetyl (Compound 4)**: The carbonyl group’s hydrogen-bonding potential and moderate steric bulk optimized interactions with catalytic residues (e.g., ALA 106 in α-amylase; GLU 411 in α-glucosidase), as evidenced by docking scores (− 7.758 and − 8.116 kcal/mol, respectively).
- **Bromo (Compound 10)**: Halogen bonding with residues (e.g., ARG 315) and balanced lipophilicity enhanced binding affinity.

2. **Steric and Electronic Trade-offs**:

- **Ester (Compound 8)**: The CH_3_CH_2_OCO group’s bulkiness reduced α-amylase inhibition but retained partial α-glucosidase activity due to flexibility in the latter’s active site.
- **Carboxyl (Compound 6)**: High polarity limited membrane permeability and enzyme access, explaining its weaker activity.

3. **Electron-Donating Groups (EDGs)**:

- **Methoxy (Compound 5)**: Lower activity compared to EWGs suggested that EDGs diminish charge stabilization in the enzyme’s catalytic pocket.

These SAR observations correlate with trends reported in Sect. **2.5** and are supported by molecular docking and MD simulation results. Together, they provide a coherent structural basis for understanding how substituent variation modulates dual inhibitory activity.

These SAR observations correlate with trends reported in Sect. "Biological activity" and are supported by molecular docking and MD simulation results. Together, they provide a coherent structural basis for understanding how substituent variation modulates dual inhibitory activity.

### Analysis of ADME characteristics of ligand

The most promising compounds, **4**, **6**, **8**, and **10**, were subjected to expected bioinformatics analysis to forecast their physicochemical and pharmacokinetic features, as well as if they may be bioavailable orally pharmaceutical agents relative to forxiga (Dapagliflozin) and acarbose. The Swiss ADME online tool was applied for assessing the drug-likeness of and the characteristic features of ADME of compounds. The test compounds had good oral bioavailability and excellent physicochemical properties, including size (MW between 150 and 500 g/mol), flexibility (maximum 10 rotatable bonds), polarity (TPSA between 20 and 140 Å), solubility (Log S less than 6), and lipophilicity (Log *P* between − 0.7 and + 5.0). It was determined that compounds varied in solubility from partially soluble to soluble, as opposed to compound **10**, which is weakly soluble^39,40^. All studied compounds followed Lipinski’s rule of five^31^, M.wt. ≤ 500 g/mol except compounds **8** and **10**, log *P* ≤ 5, TPSA ≤ 140 Å, HBA ≤ 10, and HBD < 5 are indicated^32^. As shown in Table 1, the designed compounds generally complied with Lipinski’s rule of five, exhibiting molecular weights below 510 g/mol, log *P* values under 5^33^, and acceptable topological polar surface area (TPSA) values between 94.34 and 131.64 Å^2^. In contrast, acarbose violates multiple Lipinski parameters, including its high molecular weight (645.6 g/mol), excessive number of hydrogen bond donors (14), and TPSA (329.01 Å^2^), which contribute to its poor oral bioavailability. These findings support the favorable drug-likeness and absorption potential of the synthesized compounds. Compounds **4**, **6**, **8**, and **10**’s physicochemical characteristics and drug-likeness are presented in Table 1 in comparison to forxiga (Dapagliflozin) and acarbose. Furthermore, the ADME characteristics analysis demonstrated that drugs might be taken up by the intestine, with the white of the egg model^34^ demonstrating considerable gastrointestinal absorption for all substances. The investigation showed that the drugs could not be taken up passively across the blood–brain barrier, as proven by the egg yolk model^34^. Table 2 compares the ADME analysis results for compounds **4**, **6**, **8**, and 10 to those for forxiga (Dapagliflozin) and acarbose. Generally, every ruling concept that support the conclusion that compounds **4**, **6**, **8**, and **10** exhibit favorable drug-likeness, suggesting that these substances may fulfil the permeability and bioavailability criteria of the cell membrane.

**Table 1:** The expected physicochemical properties and drug-likeness for substances **4**, **6**, **8**, and **10** compared to forxiga and acarbose.

Compounds	^a^ M.W. (g/mol)	^b^ iLog P_o/w_	^c^ Log S	^d^ TPSA (Å)	^e^ HBA	^f^ HBD	^g^ NRB	Lipinski violations
4	472.56	3.96	−5.41	111.41	5	1	10	0
6	474.53	3.53	− 5.33	131.64	6	2	10	0
8	502.58	4.28	−5.77	120.64	6	1	12	1
10	509.42	4.59	−6.37	94.34	4	1	9	2
Forxiga	408.87	2.18	−3.78	99.38	6	4	6	0
Acarbose	645.60	−6.67	2.57	329.01	19	14	13	3

^a^ *MW* molecular weight, ^b^ Log *P*o/w partition coefficient octanol/water, ^c^ Log S aqueous solubility, ^d^ *TPSA* topological polar surface area, ^e^ *HBA* number of H-bond acceptors, ^f^ *HBD* number of H-bond donors, ^g^ *NRB* number of rotatable bonds.

**Table 2:** The expected ADME analysis outcomes for substances **4**, **6**, **8**, and **10** compared to forxiga and acarbose.

Compounds	BBB permeant	GI absorption	Cytochrome P450 (CYP2D6 inhibitor)
4	No	High	Yes
6	No	Low	No
8	No	Low	No
10	No	High	Yes
Forxiga	No	High	Yes
Acarbose	No	Low	No

### Molecular docking

To achieve greater knowledge of how binding forces operate of the 1,2,4-triazole derivatives, a molecular docking analysis of four chosen substances was carried out towards the pancreatic α-amylase’s active regions and Saccharomyces cerevisiae α-glucosidase. Forxiga (Dapagliflozin) and acarbose were used to target α-amylase’s binding regions (PDB ID: 4X9Y), (PDB ID: 2QV4), (PDB ID: 1OSE) and (PDB ID: 5E0F) and α-glucosidase (PDB ID: 3A4A) and (PDB ID: 3AJ7). Compounds **1–10** exhibited stable docking poses in the α-amylase and α-glucosidase binding sites, generating multiple polar and non-polar interactions with the contained amino acid regions, as shown in Table 3S in supporting information file. The docked compounds **4**, **6, 8**, and **10** showed the best results compared with forxiga (Dapagliflozin) and acarbose as reference drugs.

#### Antidiabetic to the target enzyme α-amylase (2QV4)

##### Ligand interactions of forxiga (Dapagliflozin) and Acarbose as reference drugs

**Fig. 5:**
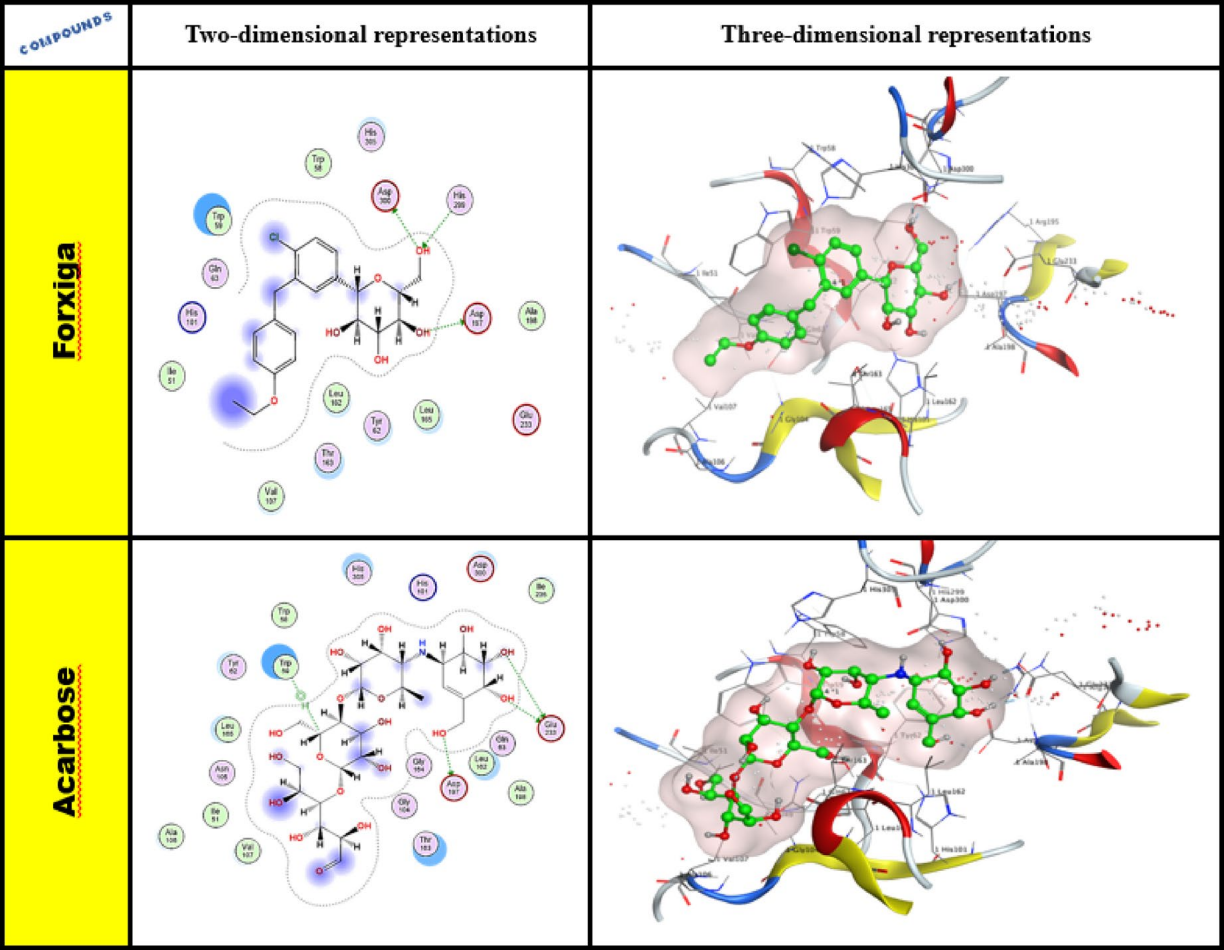
Two- and three-dimensional representations for enzyme α-amylase (PDB ID: 2QV4) for forxiga and acarbose.

The molecular docking research on forxiga and acarbose revealed a variety of interactions with binding site residues. Forxiga (Dapagliflozin), the ASP 300, ASP 197, and HIS 299 residues produced a hydrogen bonding with the hydroxy group’s lone pair of oxygen, as illustrated in Fig. 5. Acarbose can hydrogen bond with GLU 233 (A) H-donor, ASP 197 (A) H-donor, and TRP 59, which are participating in the σ-H-interaction with the cyclic ring π-system.

##### Ligand interactions of compound 4,6,8 and 10

**Fig. 6:**
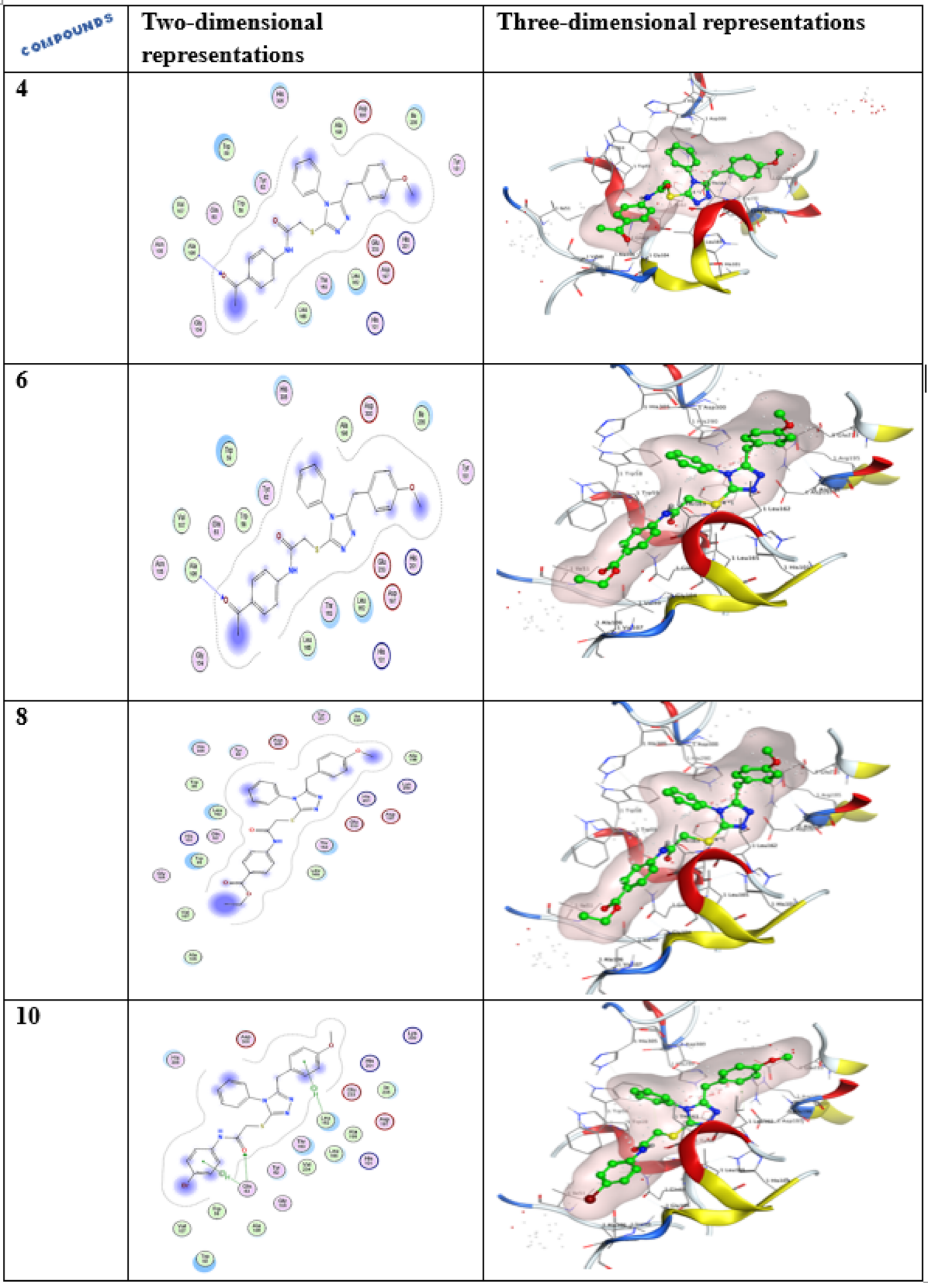
Two- and three-dimensional representations for enzyme α-amylase (PDB ID: 2QV4).

Compound **4**, the carbonyl group has the capability to bind with the ALA 106 with hydrogen bonding having a docking result of − 7.758 kcal/mol, while, compounds **6** and **10** are involved in a σ-H-interaction with the GLN 63 and LEU 162 with docking scores of − 7.396 and − 7.682 kcal/mol, correspondingly, as shown in Table 3 and Fig. 6.

**Table 3:** The docking results and amino acids contributing to interactions for substances forxiga, acarbose, **4**, **6**, **8**, and **10.**

Compounds	Docking score (Kcal/mol)	Amino acid H-bond (Bond length Ǻ)
Forxiga (Dapagliflozin)	− 6.780	ASP 300 (A) H-donor 2.86 ASP 197 (A) H-donor 2.81 HIS 299 (A) H-acceptor 2.93
Acarbose	− 8.766	GLU 233 (A) H-donor 2.84 GLU 233 (A) H-donor 2.80 ASP 197 (A) H-donor 2.95 TRP 59 (A) H-pi 4.70
4	− 7.758	ALA 106 (A) H-acceptor 3.13
6	− 7.396	GLN 63 (A) pi-H 4.49
8	− 7.924	–
10	− 7.682	GLN 63 (A) H-acceptor 2.92 GLN 63 (A) pi-H 4.42 LEU 162(A) pi-H 3.88

#### Antidiabetic to the target enzyme α-glucosidase (3AJ7)

##### Ligand interactions of forxiga and acarbose as reference drugs

**Fig. 7:**
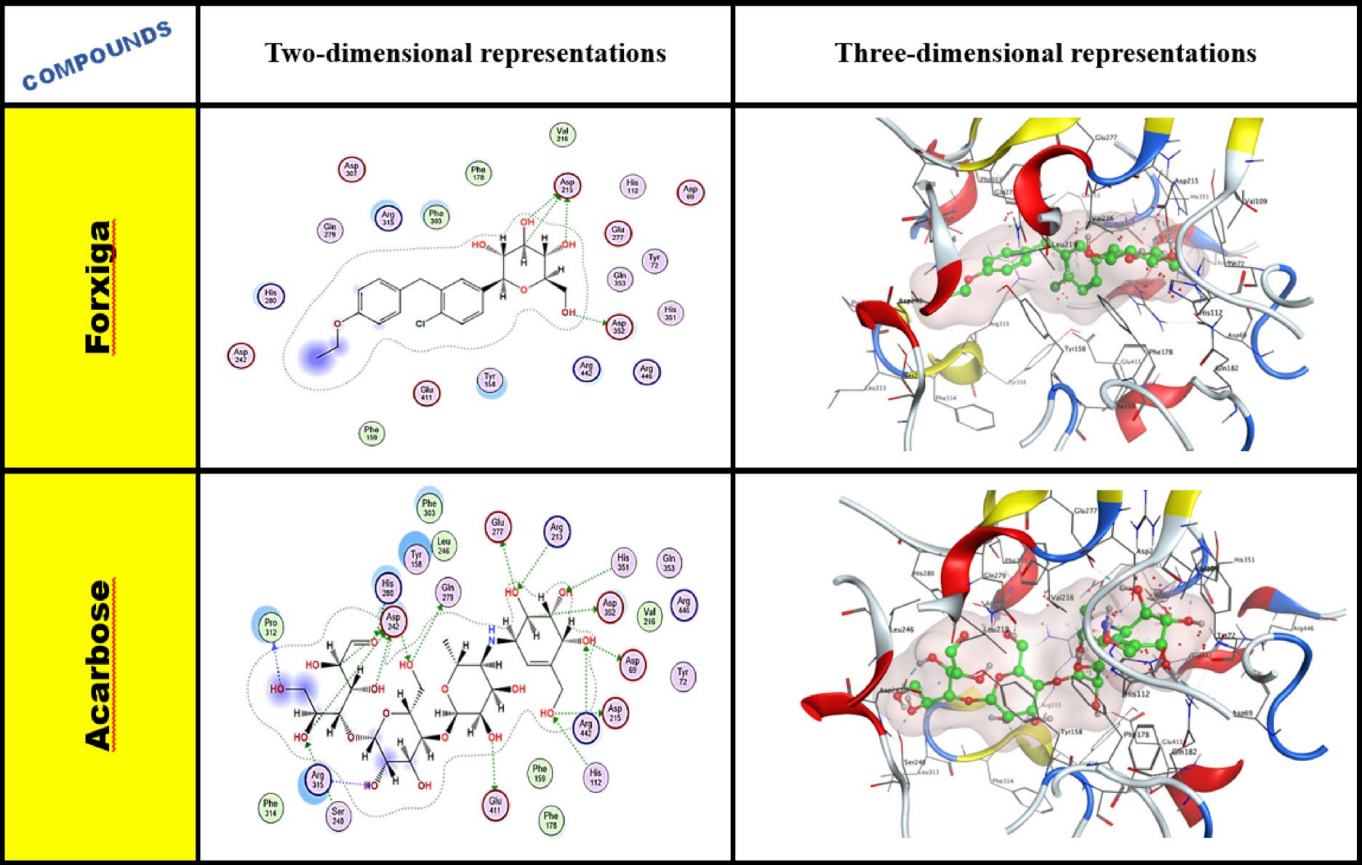
Two- and three-dimensional representations for enzyme α-glucosidase (PDB ID: 3AJ7) for forxiga and acarbose.

The molecular docking study of forxiga (Dapagliflozin) and acarbose demonstrated multiple types of interactions with the regions of the binding location. For forxiga (Dapagliflozin), the ASP 352, 4ASP 215 residues exhibited a hydrogen bonding interaction with the lone pair of oxygen of the hydroxy group and CH, as shown in Fig. 7. While the analysis of acarbose’s molecular docking is also able to hydrogen bond with more amino acids, as mentioned in Table 4.

**Table 4:** The docking results and amino acids contributing to interactions for substances forxiga (Dapagliflozin), acarbose, **4**, **6**, **8**, **9** and **10.**

Compounds	Docking score (Kcal/mol)	Amino acid H-bond (Bond length Ǻ)
Forxiga (Dapagliflozin)	− 7.893	ASP 352 (A) H-donor 2.87 ASP 215 (A) H-donor 3.02 ASP 215 (A) H-donor 2.85 ASP 215 (A) H-donor 3.07 ASP 215 (A) H-donor 3.02
Acarbose	− 10.063	PRO 312 (A) H-donor 2.73 ASP 242 (A) H-donor 2.78 ASP 242 (A) H-donor 3.10 ASP 242 (A) H-donor 2.71 GLN 279 (A) H-donor 3.09 GLU 411 (A) H-donor 2.87 ASP 352 (A) H-donor 3.32 GLU 277 (A) H-donor 2.99 ASP 69 (A) H-donor 2.58 ASP 215 (A) H-donor 2.72 ARG 315 (A) H-acceptor 3.04 SER 240 (A) H-acceptor 2.91 HIS 280 (A) H-acceptor 2.86 HIS 280 (A) H-acceptor 3.29 ARG 213 (A) H-acceptor 3.37 HIS 351 (A) H-acceptor 3.18 ARG 442 (A) H-acceptor 2.81 HIS 112 (A) H-acceptor 3.22
4	− 8.116	GLU 411 (A) H-donor 4.09 PRO 312 (A) pi-H 3.73
6	− 8.248	TYR 158 (A) H-donor 3.26 GLU 277 (A) H-donor 2.89 GLU 277 (A) H-donor 3.29 HIS 280 (A) H-acceptor 3.78 TYR 158 (A) H-pi 4.14
8	− 8.948	GLN 279 (A) H-donor 2.92 ASN 415 (A) H-acceptor 3.19
9	− 8.158	GLU 411 (A) H-donor 3.26 ASP 352 (A) H-donor 3.07
10	− 8.555	ARG 315 (A) pi-H 4.29

##### Ligand interactions of compound 4,6,8, 9 and 10

**Fig. 8:**
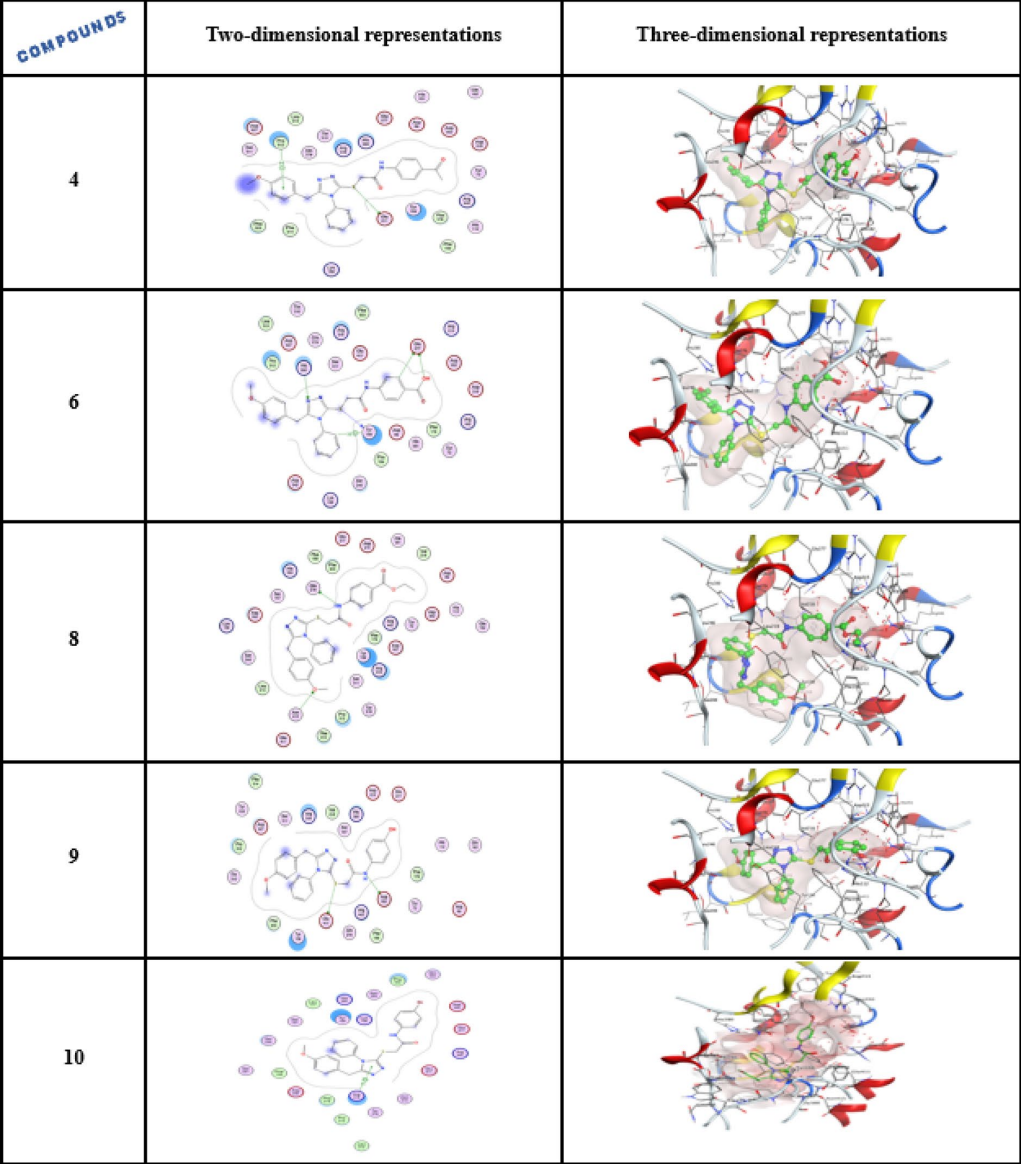
Two- and three-dimensional representations for enzyme α-glucosidase (PDB ID: 3AJ7).

All the examined compounds scored better than the commercial drug forxiga (Dapagliflozin). In compound **4**, the sulfur atom is able to hydrogen bond with the GLU 411 and PRO 312 participated in the σ-H-interaction with the phenyl ring π-system with a docking result of − 8.116 Kcal/mol, while compound **6** is able to hydrogen bond with the TYR 158, GLU 277, GLU 277, HIS 280, and TYR 158 involved in a σ-H-interaction with the phenyl ring. The molecular docking studies of compounds **8** and **9** are also able to hydrogen bond with more amino acids, whereas compound **10** is able to bond with ARG315 by pi-H, as mentioned in Table 4, Fig. 8.

#### Comparative analysis of docking interactions

Acarbose and forxiga (dapagliflozin), used as reference drugs, formed multiple hydrogen bonds and π-interactions with catalytic residues in α-amylase and α-glucosidase. Acarbose exhibited strong hydrogen bonding with GLU 233 and ASP 197, and π-stacking with TRP 59 in α-amylase, with a docking score of − 8.766 kcal/mol. In comparison, compound **4** formed hydrogen bonds with ALA 106 and π-interactions with PRO 312 in α-glucosidase, yielding a docking score of − 8.116 kcal/mol, while compound **10** engaged ARG 315 via halogen bonding and π-H interaction, with a comparable score of − 8.555 kcal/mol Table 5.

**Table 5:** Comparative docking scores and interactions of compounds **4, 10**, and reference drugs.

Compound	Enzyme	Key residues involved	Docking score (kcal/mol)
Acarbose	α-Amylase	GLU 233, ASP 197, TRP 59	− 8.766
Forxiga	α-Amylase	ASP 197, ASP 300, HIS 299	− 6.780
4	α-Amylase	ALA 106	− 7.758
10	α-Amylase	GLN 63, LEU 162	− 7.682
Acarbose	α-Glucosidase	GLU 277, ASP 352, ARG 315	− 10.063
4	α-Glucosidase	GLU 411, PRO 312	− 8.116
10	α-Glucosidase	ARG 315	− 8.555

For α-amylase, forxiga interacted with ASP 197, ASP 300, and HIS 299, with a docking score of − 6.780 kcal/mol. Compound **4** achieved a stronger binding score (− 7.758 kcal/mol) through hydrogen bonding with ALA 106 and π-interactions. Compound **10** also surpassed forxiga, forming stable contacts with GLN 63 and LEU 162 Table 5.

These results suggest that the designed compounds not only mimic key interactions seen in reference drugs but also exploit additional binding pockets via halogen bonding and optimized hydrogen bond networks, enhancing affinity and selectivity toward both target enzymes. This comparative analysis supports the relevance of the docking predictions and highlights the improved binding performance of compounds **4** and **10** over existing clinical inhibitors. The comparative analysis confirms that our triazole derivatives achieve potent inhibition through focused interactions with catalytic residues, unlike acarbose’s nonspecific binding. This aligns with their superior in vitro activity (IC_50_ values 0.19–0.31 μg/mL vs. acarbose’s 0.34 μg/mL).

### Biological activity

#### In vitro testing with α-amylase

The substances were examined for their potential to inhibit α-amylase in vitro. Acarbose is a widely used standard inhibitor of α-amylase. The superior inhibitory activity of compounds **4** and **10** over acarbose highlights their therapeutic potential and warrants further investigation into their pharmacokinetics and safety profiles. Two compounds, including **4** (IC_50_ = 0.19 ± 0.01 µg/mL) and **10** (IC_50_ = 0.26 ± 0.01 µg/mL), showed high activity relative to the reference acarbose. The presence of electron-withdrawing substituents (acetyl in compound **4** and bromo in compound **10** appears to enhance binding through electronic effects, stabilizing the inhibitor-enzyme complex. Compound **4**’s stronger activity suggests that steric and electronic factors of the acetyl group are better aligned with the enzyme’s active site geometry.

**Fig. 9:**
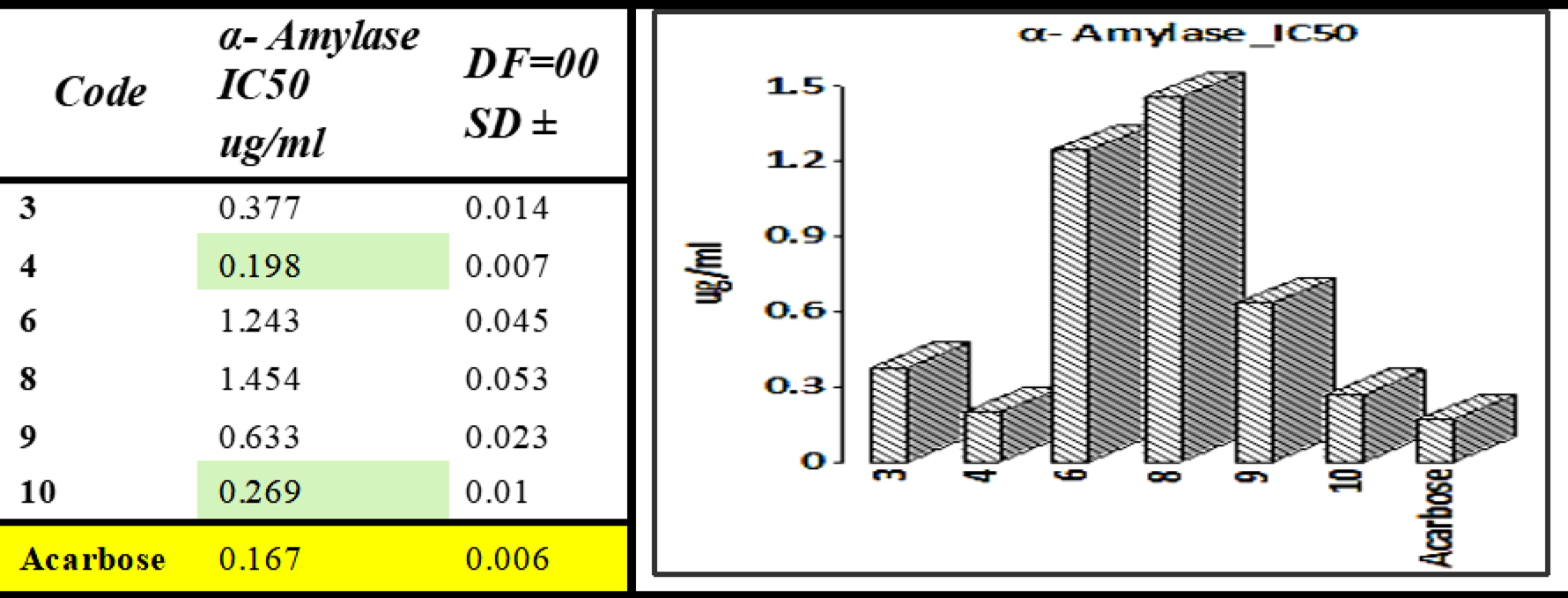
α-amylase activity of derivatives** 3**,** 4**,** 6**,** 8**,** 9**, and** 10** compared to acarbose.

Figure 9 shows a graphical depiction of the synthesized compounds and displays their IC_50_ values. The remaining four derivatives (**3**, **6**, **8**, and **9**) demonstrated superb to less suppression with corresponding IC_50_ values of 0.37 ± 0.01, 1.24 ± 0.04, 1.45 ± 0.05, and 0.63 ± 0.02 µg/mL. Compound **3** showed moderate activity, likely due to a balance of steric and electronic effects that do not optimize binding. Compounds **6** and **8** showed weak activity, which can be attributed to unfavorable steric hindrance (in compound **8**) or excessive polarity (in compound **6**), both of which disrupt efficient binding. Detailed results for α-amylase activity are shown in Table 1S.

#### In vitro testing with α-glucosidase

The synthesized compounds were evaluated at concentrations ranging from 63 to 1000 µg/ml for their ability to block α-glucosidase.

**Fig. 10:**
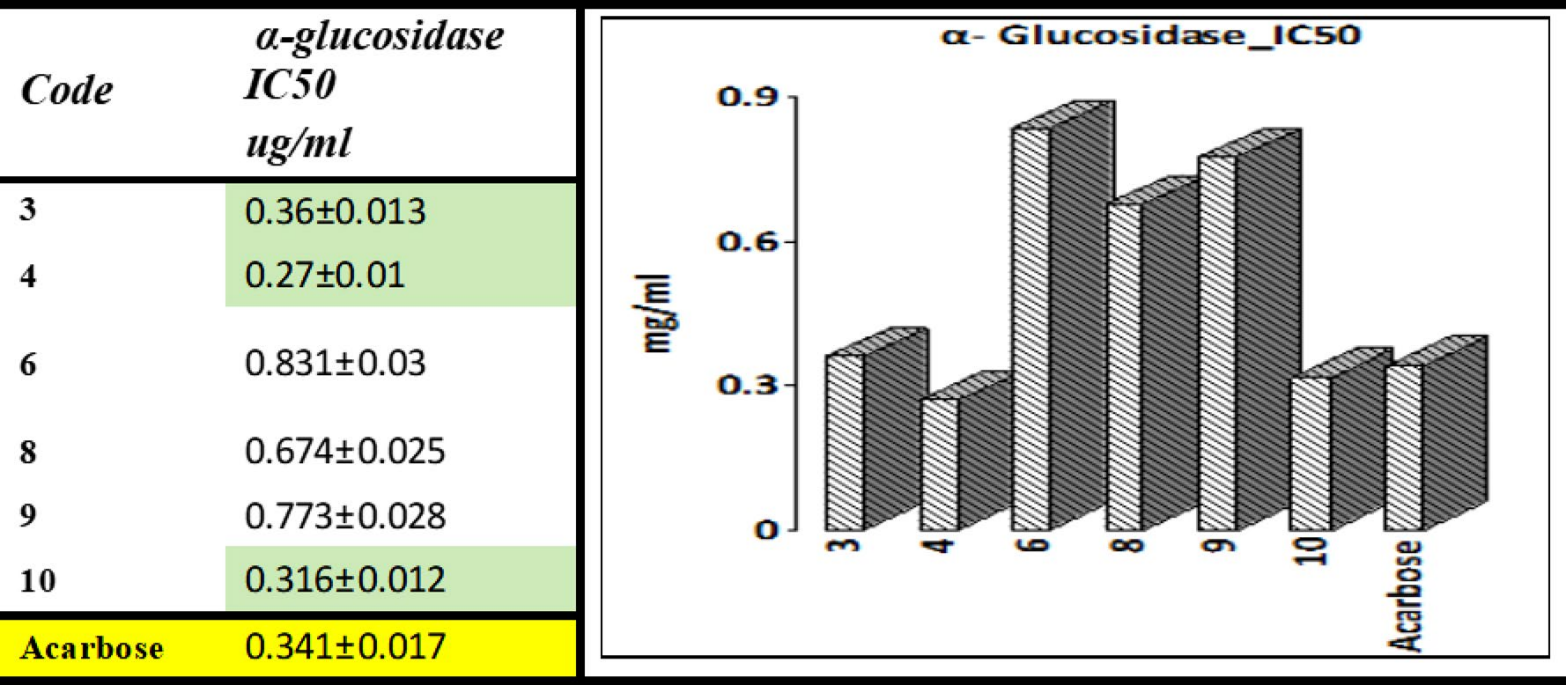
*α*-Glucosidase activity of derivatives** 3**,** 4**, **6**, **8**, **9**, and **10** compared to acarbose.

The outcomes are presented in Fig. 10. The charts show that increasing concentration leads to higher activity levels. Yet, the IC_50_ value provided the most precise comparability. Compounds’ inhibitory effects on α-glucosidase were compared using their IC_50_ values. A total of six substances were explored for potential α-glucosidase inhibitory effects. The substances had a substantial inhibitory impact on α-glucosidase, as shown in the results.

The results show that the compounds have substantial inhibitory action. Between these substances, **3**, **4,** and **10** possess the strongest inhibitory action, as demonstrated by IC_50_ values of 0.36 ± 0.01, 0.27 ± 0.01, and 0.31 ± 0.01 µg/mL, correspondingly, followed by **6**, **8,** and **9** with IC_50_ values of 0.83 ± 0.03, 0.67 ± 0.02, and 0.77 ± 0.02 µg/mL, correspondingly. Detailed results for α-glucosidase activity are shown in Table 2S.

Mechanism of Action, α-Glucosidase inhibitors delay carbohydrate breakdown into glucose, reducing postprandial blood sugar levels. The strong inhibition observed with compounds **3**, **4**, and **10** suggests they effectively block the enzyme’s active site, likely through well-placed functional groups that form stable enzyme-inhibitor complexes. Compounds **4** and **10** feature electron-withdrawing groups (acetyl and bromo, respectively) at the *para*-position, which enhance binding affinity by stabilizing interactions within the enzyme’s catalytic domain. In contrast, compounds **6**, **8**, and **9** have bulkier or less electron-attracting substituents that may introduce steric hindrance or suboptimal alignment within the binding pocket, leading to reduced efficacy.

Therapeutic Potential: The potency of compounds **3**, **4**, and **10** suggests they could serve as promising leads for developing *α*-glucosidase inhibitors. Their IC_50_ values outperform or rival existing drugs, highlighting their relevance for managing hyperglycemia in diabetes.

### Molecular dynamics analysis

To evaluate the binding affinity of compounds **4** and **10** with the target proteins α-amylase (PDB ID: 2QV4) and α-glucosidase (PDB ID: 3AJ7), we conducted molecular dynamics simulations using GROMACS 2024.2. The 4:2QV4, 10:2QV4, 4:3AJ7, and 10:3AJ7 complexes were simulated over a period of 50 ns.

**Fig. 11:**
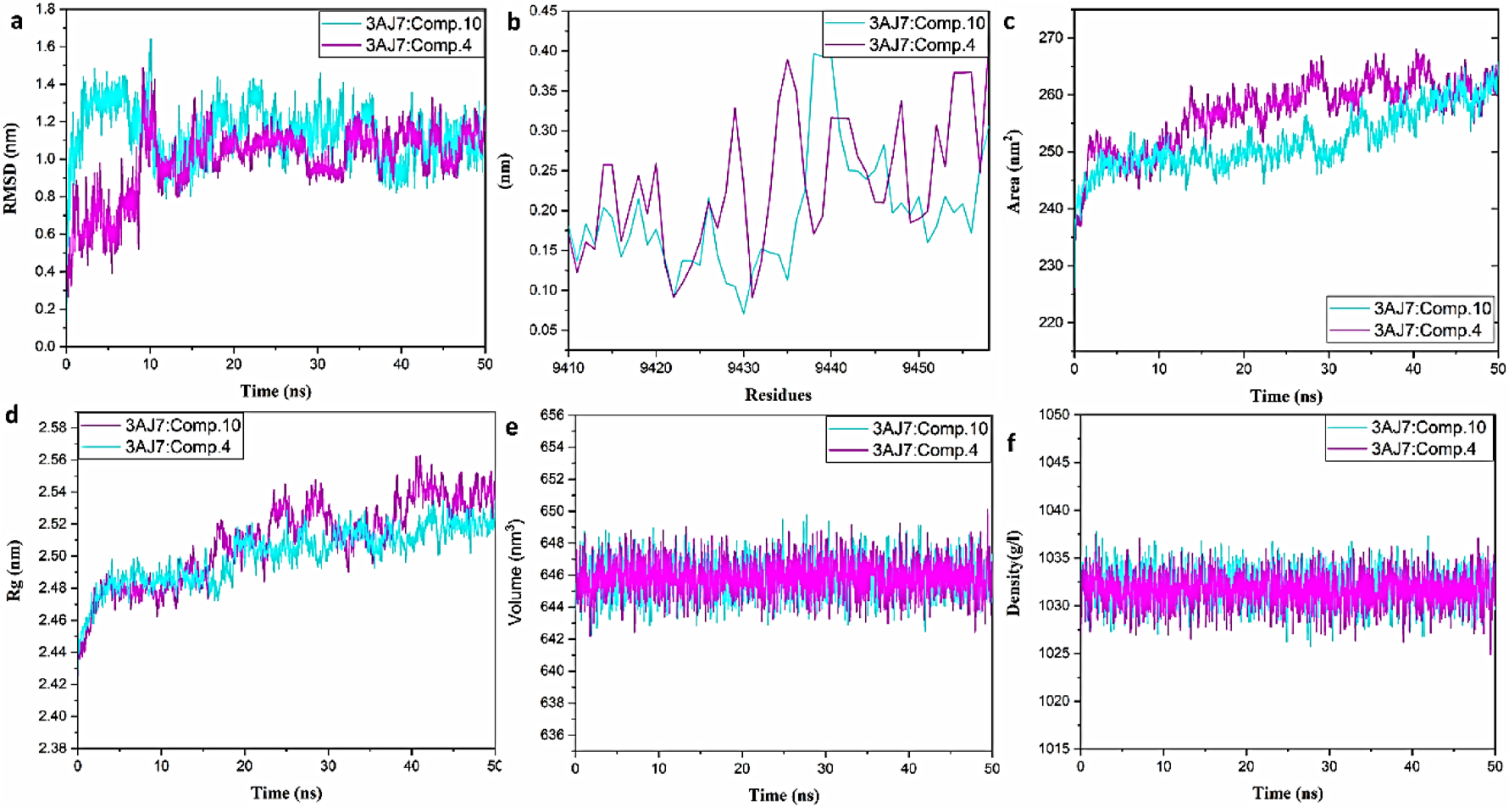
**(a)** Plot of protein backbone RMSD against time; **(b)** RMS fluctuation; **(c)** solvent-accessible surface area; **(d)** radius of gyration; **(e)** volume; and **(f)** density analysis for the complexes 3AJ7: compound **10** (pink) and 3AJ7: compound **4** (cyan).

**Fig. 12:**
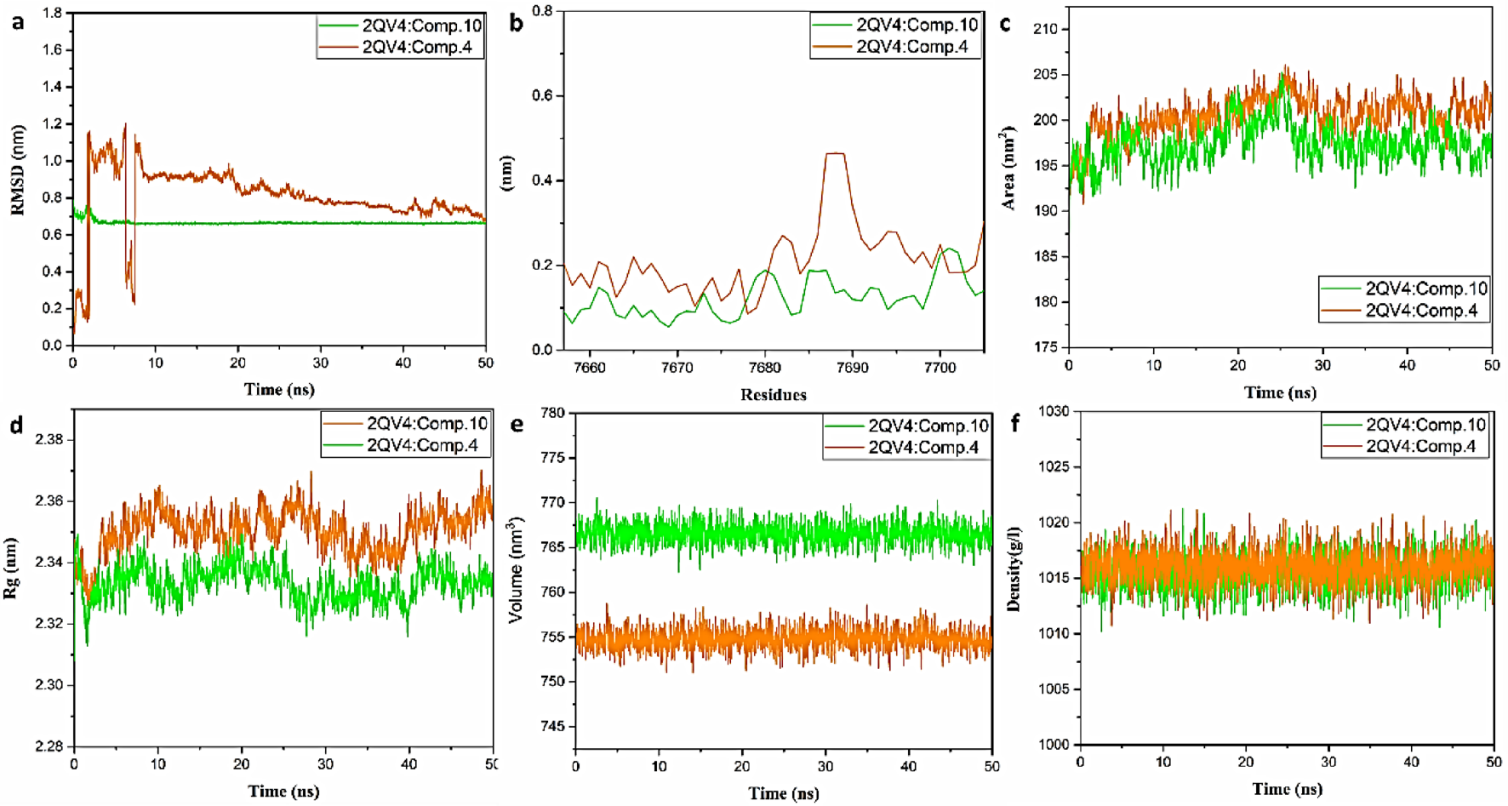
**(a)** Plot of protein backbone RMSD against time; **(b)** RMS fluctuation; **(c)** solvent-accessible surface area; **(d)** radius of gyration; **(e)** volume; and **(f)** density analysis for the complexes 2QV4: compound **10** (orange) and 2QV4: compound **4** (green).

The results, illustrated in Figs. 11a–f and 12a–f, include various simulation plots depicting the behavior of the complexes. Root Mean Square Deviation (RMSD) analysis revealed that the RMSD values remained consistently below 1.7 nm, indicating stable binding throughout the simulation period. This suggests that the overall structures of the 4:2QV4, 10:2QV4, 4:3AJ7, and 10:3AJ7 complexes remained relatively unchanged, reflecting stability in the binding interactions. Root Mean Square Fluctuation (RMSF) analysis, which assesses the flexibility of individual residues, showed minimal fluctuations. This indicates that while the complexes were stable, the binding region exhibited limited mobility or flexibility, pointing to a robust and rigid interaction between the ligands and the proteins.

Solvent Accessible Surface Area (SASA) analysis reached a maximum value of approximately 270 nm^2^ for 3AJ7 and 205 nm^2^ for 2QV4, suggesting that the interaction surfaces between compound **4** or compound **10** and 2QV4 or 3AJ7 were sufficiently exposed to the solvent, consistent with expected binding dynamics. Radius of Gyration (Rg) analysis further supported the stability of the complexes, demonstrating that the binding of compounds **4** and **10** to 3AJ7 and 2QV4 remained stable throughout the simulation. The simulation results indicate that compounds **4** and **10** bind stably to the target proteins 2QV4 and 3AJ7, with consistent RMSD values and minimal RMSF fluctuations. These findings confirm the stability and robustness of the 4:2QV4, 10:2QV4, 4:3AJ7, and 10:3AJ7 complexes throughout the 50 ns simulation period.

## Experimental

### General information

Reagents, chemical solvents, and precursors were purchased from reliable vendors including Sigma-Aldrich, Loba, Merck, Acros, and El-Gomhoria. The melting points (°C) are unadjusted and were measured using open capillaries and a Stuart SMP10 melting point device. Using silica gel 60 GF254 (E-Merck) for thin layer chromatography, all reactions were tracked and visible under a UV lamp set at a wavelength (λ) of 254 nm. NMR spectra were acquired using dimethyl sulfoxide (DMSO)-d_6_ as the solvent and tetramethylsilane (TMS) as an internal standard on a Brüker high-performance digital FT-NMR spectrometer Advance 400 MHz for ^1^H NMR and 100 MHz for ^13^C NMR (see the supporting information for the spectra). In the ^1^H NMR spectrum, the letters ‘s’, ‘d’, ‘t’, ‘q’, and ‘m’ stand for singlet, doublet, triplet, quartet and multiplet, respectively. The coupling constants (*J*) and chemical shifts (δ) are reported in hertz (Hz) and parts per million (ppm), respectively. The Perkin-Elmer CHN-elemental analyzer was used for the microanalysis.

### Chemistry

#### Standard synthetic method of compound (S2)

Compound **S2** is a known intermediate and was synthesized according to previously reported methods^35,36^. A mixture of 2-(4-methoxyphenyl) acetohydrazide (1.0 equiv.) and phenyl isothiocyanate (1.1 equiv.) in ethanol was refluxed for 4 h. After cooling, 10% NaOH was added, and the mixture was refluxed for an additional 2 h. Acidification with HCl yielded the crude product, which was recrystallized from ethanol (Yield: 75%, m.p.: 180–182 °C).

#### Standard synthetic method of compounds (S4)

Compounds **S4** are known intermediates and were synthesized according to previously reported methods^37,38^. Substituted aniline (1.0 equiv.) was reacted with 2-chloroacetyl chloride (1.2 equiv.) in acetic acid at 0 °C to r. t. for 3 h. The precipitate was filtered and recrystallized from ethanol/water (Yields: 70–85%).

#### Standard synthetic method of compounds (1–5) and compounds (7, 10)

A mixture of compound **S2** (0.3 g, 1 mmol) and NaOH (0.06 g, 1.5 mmol) was refluxed with stirring in absolute ethanol (5 ml) for 0.5 h, then cooled to r. t. The appropriate 2-chloro-*N*-phenylacetamide derivative **S4** (1.05 mmol) in absolute ethanol (5 ml) was dropped gradually to the above reaction mixture and then refluxed with stirring for an additional 1.5 h. Next, under lower pressure, the reaction mixture was evaporated until it was dry, yielding residue. After adding ice water to the residue, stir the mixture for an additional 20 min. In order to obtain the crude products, the precipitates were finally vacuum-filtered, completely cleaned with water, and dried. Recrystallization of the crude products from the suitable solvent gives the pure target compounds.

##### *N*-(2-chlorophenyl)-2-((5-(4-methoxybenzyl)-4-phenyl-4*H*-1,2,4-triazol-3-yl)thio)acetamide (1)

White fluffy material (absolute ethanol: glacial acetic acid, 9: 1); yield: 72%; m.p.: 200–202 °C. I.R. (KBr, υcm^−1^): 3250 (NH), 1683 (C=O, amide), 1594 (C=N). ^1^H NMR (DMSO-d_6_): δ = 3.67 (s, 3H, *O*-CH_3_), 3.91 (s, 2H, CH_2_), 4.14 (s, 2H, CH_2_-S), 6.71 (d, 2H, *J* = 8.60 Hz, Ar-H), 6.80 (d, 2H, *J* = 8.60 Hz, Ar-H), 7.18 (t, 1H, *J* = 7.31 Hz, Ar-H), 7.26 (d, 1H, *J* = 6.32 Hz, Ar-H), 7.32 (t, 1H, *J* = 7.24 Hz, Ar-H), 7.47–7.54 (m, 5H, Ar-H), 7.76 (d, 1H, *J* = 7.88 Hz, Ar-H), 9.92 ppm (s, 1H, NH). ^13^C NMR (DMSO-d_6_): δ = 29.89 (CH_2_), 36.47 (CH_2_-S), 55.01 (*O*-CH_3_), 113.7, 125.1, 125.6, 126.2, 127.2, 127.5, 129.4, 129.5, 129.8, 130.0, 132.7, 134.6 (Ar-C), 149.9 (C-3), 154.9 (C-5), 157.9 (Ar-C) and 166.3 ppm (C=O). Anal. Calcd. for C_24_H_21_ClN_4_O_2_S (464.97): C, 62.00; H, 4.55; N, 12.05%. Found: C, 62.07; H, 4.56; N, 12.03%.

##### *N*-(2,5-dimethylphenyl)-2-((5-(4-methoxybenzyl)-4-phenyl-4*H*-1,2,4-triazol-3-yl)thio)acetamide (2)

Off-white scales (absolute ethanol: glacial acetic acid, 9: 1); yield: 65%; m.p.: 168–170 °C. I.R. (KBr, υcm^−1^): 3229 (NH), 1681 (C=O, amide), 1530 (C=N). ^1^H NMR (DMSO-d_6_): δ = 2.12 (s, 3H, CH_3_), 2.22 (s, 3H, CH_3_), 3.67 (s, 3H, *O*-CH_3_), 3.91 (s, 2H, CH_2_), 4.10 (s, 2H, CH_2_-S), 6.72 (d, 2H, *J* = 8.56 Hz, Ar-H), 6.80 (d, 2H, *J* = 8.48 Hz, Ar-H), 6.87 (d, 1H, *J* = 7.60 Hz, Ar-H), 7.06 (d, 1H, *J* = 7.64 Hz, Ar-H), 7.25–7.28 (d, *J* = 7.64, 1H, Ar-H), 7.49–7.56 (m, 5H, Ar-H), 9.61 ppm (s, 1H, NH). ^13^C NMR (DMSO-d_6_): δ = 17.31 (CH_3_), 20.71 (CH_3_), 29.88 (CH_2_), 36.40 (CH_2_-S), 54.98 (*O*-CH_3_), 113.7, 124.8, 125.8, 127.2, 127.5, 128.0, 129.4, 129.7, 129.9, 130.1, 132.8, 134.9, 135.7 (Ar-C), 150.0 (C-3), 154.8 (C-5), 157.9 (Ar-C) and 165.7 ppm (C=O). Anal. Calcd. for C_26_H_26_N_4_O_2_S (458.58): C, 68.10; H, 5.72; N, 12.22%. Found: C, 68.05; H, 5.70; N, 12.24%.

##### 2-((5-(4-methoxybenzyl)-4-phenyl-4*H*-1,2,4-triazol-3-yl)thio)-*N*-phenylacetamide (3)

White fluffy material (90% acetic acid); yield: 70%; m.p.: 183-185 °C. I.R. (KBr, υcm^−1^): 3210 (NH), 1665 (C=O, amide), 1591 (C=N). ^1^H NMR (DMSO-d_6_): δ = 3.67 (s, 3H, *O*-CH_3_), 3.90 (s, 2H, CH_2_), 4.10 (s, 2H, CH_2_-S), 6.71 (d, 2H, *J* = 8.52 Hz, Ar-H), 6.80 (d, 2H, *J* = 8.44 Hz, Ar-H), 7.05 (t, 1H, *J* = 7.24 Hz, Ar-H), 7.26–7.32 (m, 4H, Ar-H), 7.50–7.55 (m, 5H, Ar-H), 10.31 ppm (s, 1H, NH). ^13^C NMR (DMSO-d_6_): δ = 30.23 (CH_2_), 37.46 (CH_2_-S), 55.46 (*O*-CH_3_), 114.2, 119.5, 123.9, 127.7, 128.0, 129.2, 129.8, 130.2, 130.4, 133.2, 139.2 (Ar-C), 150.4 (C-3), 155.3 (C-5), 158.3 (Ar-C) and 166.0 ppm (C=O). Anal. Calcd. for C_24_H_22_N_4_O_2_S (430.53): C, 66.96; H, 5.15; N, 13.01%. Found: C, 66.91; H, 5.16; N, 13.04%.

##### *N*-(4-acetylphenyl)-2-((5-(4-methoxybenzyl)-4-phenyl-4*H*-1,2,4-triazol-3-yl)thio)acetamide (4)

White powder (75% acetic acid); yield: 63%; m.p.: 186–188 °C. I.R. (KBr, υcm^−1^): 3230 (NH), 1680 (br, 2C=O, ketone, amide), 1520 (C=N). ^1^H NMR (DMSO-d_6_): δ = 2.52 (s, 3H, COCH_3_), 3.67 (s, 3H, *O*-CH_3_), 3.89 (s, 2H, CH_2_), 4.13 (s, 2H, CH_2_-S), 6.71 (d, 2H, *J* = 8.80 Hz, Ar-H), 6.79 (d, 2H, *J* = 8.40 Hz, Ar-H), 7.26 (d, 2H, *J* = 6.4 Hz, Ar-H), 7.51 (t, 3H, *J* = 7.6 Hz, Ar-H), 7.67 (d, 2H, *J* = 8.8 Hz, Ar-H), 7.92 (d, 2H, *J* = 8.8 Hz, Ar-H), 10.65 ppm (s, 1H, NH). ^13^C NMR (DMSO-d_6_): δ = 29.88 (COCH_3_), 30.35 (CH_2_), 36.99 (CH_2_-S), 54.99 (*O*-CH_3_), 113.7, 118.3, 127.2, 127.5, 129.4, 129.5, 129.7, 129.9, 131.9, 132.7, 143.0 (Ar-C), 149.8 (C-3), 154.9 (C-5), 157.9 (Ar-C), 166.2 (C=O, amide) and 196.5 ppm (COCH_3_). Anal. Calcd. for C_26_H_24_N_4_O_3_S (472.16): C, 66.08; H, 5.12; N, 11.86%. Found: C, 66.13; H, 5.10; N, 11.83%.

##### 2-((5-(4-methoxybenzyl)-4-phenyl-4*H*-1,2,4-triazol-3-yl)thio)-*N*-(4-methoxyphenyl)acetamide (5)

Faint violet fine material (80% acetic acid); yield: 54%; m.p.: 188–190 °C. I.R. (KBr, υcm^−1^): 3220 (NH), 1641 (C=O), 1490 (C=N). ^1^H NMR (DMSO-d_6_): δ = 3.67 (s, 3H, *O*-CH_3_), 3.71 (s, 3H, *O*-CH_3_), 3.89 (s, 2H, CH_2_), 4.06 (s, 2H, CH_2_-S), 6.71 (d, 2H, *J* = 8.48 Hz, Ar-H), 6.80 (d, 2H, *J* = 8.44 Hz, Ar-H), 6.86 (d, 2H, *J* = 8.84 Hz, Ar-H), 7.27 (d, 2H, *J* = 7.16 Hz, Ar-H), 7.43 (d, 2H, *J* = 8.8 Hz, Ar-H), 7.50–7.56 (m, 3H, Ar-H), 10.16 ppm (s, 1H, NH). ^13^C NMR (DMSO-d_6_): δ = 30.35 (CH_2_), 37.30 (CH_2_-S), 55.46 (CH_3_), 55.63 (CH_3_), 114.1, 114.4, 121.1, 127.7, 128.0, 129.8, 130.2, 130.4, 132.3, 133.2 (Ar-C), 150.4 (C-3), 155.3 (C-5), 155.8, 158.3 (Ar-C) and 165.4 ppm (C=O). Anal. Calcd. for C_25_H_24_N_4_O_3_S (460.55): C, 65.20; H, 5.25; N, 12.17%. Found: C, 65.26; H, 5.24; N, 12.21%.

##### *N*-(4-fluorophenyl)-2-((5-(4-methoxybenzyl)-4-phenyl-4*H*-1,2,4-triazol-3-yl)thio)acetamide (7)

White fluffy material (90% acetic acid); yield: 89%; m.p.: 190–192 °C. I.R. (KBr, υcm^−1^): 3200 (NH), 1651 (C=O), 1588 (C=N). ^1^H NMR (DMSO-d_6_): δ = 3.66 (s, 3H, *O*-CH_3_), 3.89 (s, 2H, CH_2_), 4.08 (s, 2H, CH_2_-S), 6.71 (d, 2H, *J* = 8.56 Hz, Ar-H), 6.79 (d, 2H, *J* = 8.52 Hz, Ar-H), 7.14 (t, 2H, *J* = 8.8 Hz, Ar-H), 6.25 (d, 2H, *J* = 6.4 Hz, Ar-H), 7.50–7.57 (m, 5H, Ar-H), 10.37 ppm (s, 1H, NH); ^13^C NMR (DMSO-d_6_): δ = 29.64 (CH_2_), 36.97 (CH_2_-S), 55.14 (*O*-CH_3_), 113.8, 115.3, 121.0, 127.3, 127.6, 129.5, 129.9, 130.0, 132.8, 135.2 (Ar-C), 150.1 (C-3), 155.1 (C-5), 157.2, 158.0, 159.4 (Ar-C) and 165.5 ppm (C=O). Anal. Calcd. for C_24_H_21_FN_4_O_2_S (448.52): C, 64.27; H, 4.72; N, 12.49. Found: C, 64.32; H, 4.73; N, 12.45.

##### *N*-(4-bromophenyl)-2-((5-(4-methoxybenzyl)-4-phenyl-4*H*-1,2,4-triazol-3-yl)thio)acetamide (10)

White fluffy material (90% acetic acid); yield: 88%; m.p.: 201–203 °C. I.R. (KBr, υcm^−1^): 3210 (NH), 1680 (C=O), 1599 (C=N). ^1^H NMR (DMSO-d_6_): δ = 3.67 (s, 3H, *O*-CH_3_), 3.90 (s, 2H, CH_2_), 4.10 (s, 2H, CH_2_-S), 6.71 (d, 2H, *J* = 8.0 Hz, Ar-H), 6.80 (d, 2H, *J* = 8.0 Hz, Ar-H), 7.26 (d, 2H, Ar-H), 7.48–7.54 (m, 7H, Ar-H), 10.45 ppm (s, 1H, NH). ^13^C NMR (DMSO-d_6_): δ = 29.77 (CH_2_), 37.05 (CH_2_-S), 55.00 (*O*-CH_3_), 113.5, 115.1, 121.0, 127.2, 127.5, 129.4, 129.7, 129.9, 131.6, 132.7, 138.1 (Ar-C), 149.8 (C-3), 154.8 (C-5), 157.9 (Ar-C) and 165.7 ppm (C=O). Anal. Calcd. for C_24_H_21_BrN_4_O_2_S (509.42): C, 56.59; H, 4.16; N, 11.00%. Found: C, 56.51; H, 4.18; N, 11.07%.

#### Synthetic method of compound (6)

A mixture of compound **S2** (0.3 g, 1 mmol) and NaOH (0.06 g, 1.5 mmol) was refluxed with stirring in absolute ethanol (5 ml) for 0.5 h, then cooled to r. t. The appropriate 2-chloro-*N*-phenylacetamide derivative **S4** (1.05 mmol) in absolute ethanol (5 ml) was dropped gradually to the above reaction mixture and then refluxed with stirring for an additional 1.5 h. Next, under lower pressure, the reaction mixture was evaporated until it was dry, yielding residue. After adding acidified ice water to the residue, stir the mixture for an additional 20 min. In order to obtain the crude product, the precipitate was finally vacuum-filtered, completely cleaned with water, and dried. Recrystallization of the crude product from the suitable solvent gives the pure target compound.

##### 4-(2-((5-(4-methoxybenzyl)-4-phenyl-4*H*-1,2,4-triazol-3-yl)thio)acetamido)benzoic acid (6)

White powder (glacial acetic acid); yield: 93%; m.p.: 231–233 °C. I.R. (KBr, υcm^−1^): 3210 (OH and NH), 1681 (2C=O, acid and amide), 1509 (C=N). ^1^H NMR (DMSO-d_6_): δ = 3.67 (s, 3H, *O*-CH_3_), 3.89 (s, 2H, CH_2_), 4.13 (s, 2H, CH_2_-S), 6.71 (d, 2H, *J* = 8.8 Hz, Ar-H), 6.79 (d, 2H, *J* = 8.8 Hz, Ar-H), 7.26 (t, 3H, *J* = 6.4 Hz, Ar-H), 7.50 (d, 2H, *J* = 7.6 Hz, Ar-H), 7.65 (d, 2H, *J* = 8.8 Hz, Ar-H), 7.89 (d, 2H, *J* = 8.4 Hz, Ar-H), 10.62 (s, 1H, NH), 12.71 ppm (br, 1H, OH). ^13^C NMR (DMSO-d_6_): δ = 29.90 (CH_2_), 37.16 (CH_2_-S), 55.01 (*O*-CH_3_), 113.7, 118.4, 125.4, 127.2, 127.5, 129.4, 129.8, 130.0, 130.4, 132.8, 142.7 (Ar-C), 149.8 (C-3), 154.9 (C-5), 158.0 (Ar-C), 166.1 (C=O, amide) and 166.9 ppm (C=O, acid). Anal. Calcd. for C_25_H_22_N_4_O_4_S (474.54): C, 63.28; H, 4.67; N, 11.81%. Found: C, 63.22; H, 4.69; N, 11.84%.

#### Synthetic method of compound (8)

A mixture of compound **S2** (0.3 g, 1 mmol) and triethylamine (0.21 ml, 1.5 mmol) was stirred at r. t. in absolute ethanol (5 ml) for 20 min. The appropriate 2-chloro-*N*-phenylacetamide derivative **S4** (1.05 mmol) in absolute ethanol (5 ml) was dropped gradually to the above reaction mixture and then stirred for another 3 h. Next, under lower pressure, the reaction mixture was evaporated until it was dry, yielding residue. After adding ice water to the residue, stir the mixture for an additional 20 min. In order to obtain the crude product, the precipitate was finally vacuum-filtered, completely cleaned with water, and dried. Recrystallization of the crude product from the suitable solvent gives the pure target compound.

##### Ethyl 4-(2-((5-(4-methoxybenzyl)-4-phenyl-4*H*-1,2,4-triazol-3-yl)thio)acetamido)benzoate (8)

White fluffy material (absolute ethanol); yield: 89%; m.p.: 162–164 °C. I.R. (KBr, υcm^−1^): 3220 (NH), 1696 (br, 2C=O, ester and amide), 1513 (C=N). ^1^H NMR (DMSO-d_6_): δ = 1.30 (t, 3H, ^3^*J* = 6.8 Hz, CH_2_CH_3_), 3.67 (s, 3H, *O*-CH_3_), 3.89 (s, 2H, CH_2_), 4.14 (s, 2H, CH_2_-S), 4.27 (q, 2H, ^*3*^*J* = 6.8 Hz, CH_2_CH_3_), 6.71 (d, 2H, *J* = 8.0 Hz, Ar-H), 6.81 (d, 2H, *J* = 8.0 Hz, Ar-H), 7.25 (d, 2H, *J* = 6.4 Hz, Ar-H), 7.50 (d, 3H, *J* = 7.2 Hz, Ar-H), 7.67 (d, 2H, *J* = 8.4 Hz, Ar-H), 7.91 (d, 2H, *J* = 8.0 Hz, Ar-H), 10.65 ppm (s, 1H, NH). ^13^C NMR (DMSO-d_6_): δ = 14.20 (CH_3_), 30.04 (CH_2_), 37.05 (CH_2_-S), 54.99 (*O*-CH_3_), 60.59 (*O*-CH_2_), 113.7, 118.2, 123.9, 127.2, 127.5, 129.4, 129.7, 129.9, 130.3, 132.7, 143.0 (Ar-C), 149.8 (C-3), 154.9 (C-5), 157.9 (Ar-C), 165.2 (C=O, amide), 166.2 ppm (C=O, ester). Anal. Calcd. for C_27_H_26_N_4_O_4_S (502.59): C, 64.53; H, 5.21; N, 11.15%. Found: C, 64.57; H, 5.22; N, 11.11%.

#### Synthetic method of compound (9)

A mixture of compound **S2** (0.3 g, 1 mmol) and NaOH (0.06 g, 1.5 mmol) was refluxed with stirring in absolute ethanol (5 ml) for 0.5 h, then cooled to r. t. The 2-chloro-*N*-phenylacetamide derivative **S4** (1.05 mmol) in absolute ethanol (5 ml) was dropped gradually to the above reaction mixture and then refluxed with stirring for an additional 1.5 h. Next, under lower pressure, the reaction mixture was evaporated until it was dry, yielding residue. After adding acidified ice water to the residue, stir the mixture for an additional 20 min. The obtained crude product in the form of sticky material was completely cleaned with water, and dried. Recrystallization of the crude product from the suitable solvent gives the pure target compound.

##### *N*-(4-hydroxyphenyl)-2-((5-(4-methoxybenzyl)-4-phenyl-4*H*-1,2,4-triazol-3-yl)thio)acetamide (9)

Brown fine material (glacial acetic acid); yield: 67%; m.p.: 227–229 °C. I.R. (KBr, υcm^−1^): 3470 (OH), 3200 (NH), 1667 (C=O), 1585 (C=N). ^1^H NMR (DMSO-d_6_): δ = 3.67 (s, 3H, *O*-CH_3_), 3.89 (s, 2H, CH_2_), 4.04 (s, 2H, CH_2_-S), 6.68–6.73 (m, 4H, Ar*-*H), 6.80 (d, 2H, *J* = 8.44 Hz, Ar*-*H), 7.25 (d, 2H, *J* = 6.88 Hz, Ar*-*H), 7.31 (d, 2H, *J* = 8.64 Hz, Ar*-*H), 7.50–7.55 (m, 3H, Ar*-*H), 9.22 (s, 1H, OH), 10.05 ppm (s, 1H, NH). ^13^C NMR (DMSO-d_6_): δ = 29.89 (CH_2_), 37.15 (CH_2_-S), 55.00 (*O*-CH_3_), 114.0, 115.0, 120.6, 127.2, 127.6, 129.4, 129.6, 129.9, 130.3, 132.7 (Ar-C), 149.9 (C-3), 153.5 (C-5), 154.8, 157.9 (Ar-C), 164.7 ppm (C=O). Anal. Calcd. for C_24_H_22_N_4_O_3_S (446.53): C, 64.56; H, 4.97; N, 12.55%. Found: C, 64.50; H, 4.98; N, 12.51%.

### ADMET properties

Promising compounds **4**, **6**, **8**, and **10** underwent in silico bioinformatic studies to predict their pharmacokinetics (liver metabolizing enzyme Cytochrome P450 metabolism, distribution, and GIT absorption) and physicochemical properties (size, lipophilicity, solubility, polarity, and flexibility). Additionally, in contrast to acarbose and forxiga (Dapagliflozin), if they are candidates for oral bioavailability (using Lipinski’s criterion). The compounds were assessed for drug-likeness and ADME qualities using the Swiss ADME online tool (www.SwissADME.ch/, accessed May 2, 2023)^39^.

### Molecular docking

Molecular docking simulations were conducted with the Molecular Operating Environment (MOE) software 2022, as reported in the literature^40,41^. The MOE builder was used to generate ligands, which were 3D protonated and assigned partial charges before being energy-minimized using the MMFF94x force field. Forxiga (Dapagliflozin) and acarbose were used to target the binding regions of α-amylase (PDB ID: 4X9Y)^42^, (PDB ID: 2QV4)^43^, (PDB ID: 1OSE)^44^ and (PDB ID: 5E0F)^45^ and α-glucosidase (PDB ID: 3A4A)^46^ and (PDB ID: 3AJ7)^47^, which were got from the RCSB-Protein Data Bank. Proteins were prepared using traditional techniques such as 3D-protonation, automated atom type, and linkage correction. They were then potentially fixed. MOE’s alpha site finder was utilized to identify active regions. MOE’s induced fit docking technology was used. Validation typically involves the original ligand being re-docked against the active regions of 2QV4 and 3AJ7 with RMSD values < 2.0 Å. The triangle matcher approach and the London dG scoring function were used to investigate ligand interactions at active sites, with bond rotation as the initial scoring function. The GBVI/WSA dG forcefield-based scoring mechanism was employed as a secondary scoring function, yielding five poses out of thirty. Poses with higher S-values and lower RMSD were identified.

### Biological assay

#### In vitro testing with α-amylase

Based on the manufacturer’s instructions as previously described^48,49^, the in vitro α-amylase suppression effect for the synthesized 1,2,4-triazole derivatives **3**, **4**, **6**, **8**, **9**, and **10** was conducted using BioVision’s α-Amylase Inhibitor Screening Kit (Catalogue #K482-100) and a multi-well spectrophotometer (ELISA reader) using a 96-well microplate at (OD = 405 nm). The α-amylase inhibition assay was performed using a modified DNSA (3,5-dinitrosalicylic acid) method. Test compounds (dissolved in DMSO) were serially diluted (100, 10, 1, 0.1, and 0.01 µg/mL) and pre-incubated with porcine pancreatic α-amylase (0.5 U/mL in phosphate buffer, pH 6.8) at 37 °C for 10 min. Starch solution (1% w/v) was added, and the reaction proceeded for 30 min at 37 °C. The reaction was terminated by adding DNSA reagent, followed by heating at 85 °C for 10 min. Absorbance was measured at 540 nm using a microplate reader. Acarbose was used as a positive control. The percentage inhibition was calculated as: [(A_control_–A_sample_)/A_control_] × 100, where A_control_ represents enzyme activity without inhibitor. IC_50_ values were determined from dose-response curves using GraphPad Prism. Kinetic parameters (K_Activity_) were derived from ∆RFU (Relative Fluorescence Units) and slope values Table 1S.

#### In vitro testing with α-glucosidase

Using BioVision’s α-Glucosidase Inhibitor Screening Kit (Colorimetric) (Catalogue #K938-100) at (OD: 410 nm), the in vitro α-glucosidase suppression effect for the designed 1,2,4-triazole derivatives **3**, **4**, **6**, **8**, **9**, and **10** was carried out. The results were analyzed on a multi-well spectrophotometer (ELISA reader) using a 96-well microplate in compliance with the manufacturer’s instructions as previously described^49,50^. The α-glucosidase inhibition assay was conducted using Saccharomyces cerevisiae α-glucosidase (0.5–1 U/mL in phosphate buffer, pH 6.8). Test compounds (63–1000 µg/mL) were pre-incubated with the enzyme at 37 °C for 10 min, followed by addition of pNPG (5 mM) and further incubation for 30 min. The reaction was stopped with 0.2 M Na_2_CO_3_, and absorbance was measured at 405 nm. Acarbose served as the positive control. Inhibition (%) was calculated as above, and IC_50_ values were derived from nonlinear regression analysis Table 2S.

### Molecular dynamics analysis

Molecular dynamics (MD) simulations were conducted to investigate the interactions between α-amylase (PDB ID: 2QV4) and α-glucosidase (PDB ID: 3AJ7) with 1,2,4-triazole derivatives **4** and **10**. All simulations were performed using GROMACS 2024.2, employing the CHARMM27 force field to describe atomic interactions. Protein structures were prepared by assigning the appropriate force field parameters and solvating them in TIP3P water molecules. Ligand structures were converted into a compatible format and positioned at the active site of each enzyme. The triclinic simulation box was defined with a minimum 1.0 nm buffer between the solute and the box edges. To neutralize the system, counterions were added by replacing an equivalent number of water molecules. Energy minimization was performed using the steepest descent algorithm to eliminate steric clashes and optimize the system’s initial configuration. The system was then equilibrated in two phases: first under constant volume and temperature (NVT) for 10 ns, where a velocity-rescaling thermostat was applied to regulate temperature, followed by constant pressure and temperature (NPT) for 10 ns, using the Parrinello-Rahman barostat to stabilize system density. Throughout equilibration, positional restraints were applied to the ligand to prevent large conformational fluctuations. Subsequently, production MD simulations were conducted in the NPT ensemble for 50 ns, maintaining biologically relevant temperature and pressure conditions. Post-simulation trajectory analysis included recentering and periodic boundary condition (PBC) corrections to remove system drifts and ensure accurate structural interpretation.

## Conclusion

In this study, we designed and synthesized a novel series of 1,2,4-triazole derivatives incorporating a thioacetamide linker for dual inhibition of α-amylase and α-glucosidase. Among the synthesized compounds, compounds **4** and **10** demonstrated the most potent in vitro inhibitory activity, surpassing the reference drug acarbose. Molecular docking and dynamics simulations confirmed strong and stable interactions with key catalytic residues in both target enzymes. Furthermore, in silico ADMET profiling indicated favorable pharmacokinetic properties and oral drug-likeness for the top candidates. These findings support the therapeutic potential of triazole-based scaffolds in managing postprandial hyperglycemia and highlight a promising direction for the development of next-generation antidiabetic agents. Future work will focus on in vivo validation, toxicity assessment, and pharmacokinetic profiling of the most active candidates to advance them toward clinical development.

## Electronic supplementary material

Below is the link to the electronic supplementary material.

Supplementary Material 1

## Data Availability

Data is provided within the manuscript or supplementary information files.
