# Correction: Dual α-amylase and α-glucosidase inhibition by 1,2,4-triazole derivatives for diabetes treatment

**DOI:** 10.1038/s41598-025-15989-4

**Published:** 2025-08-15

**Authors:** Mohammed A. Marzouk, Elsayed M. Mahmoud, Wesam S. Shehab, Sherif M. Fawzy, Samar M. Mohammed, Mahmoud Ashraf Abdel-Razek, Ghada E. Khedr, Doaa A. Elsayed

**Affiliations:** 1 Department of Pharmaceutical Organic Chemistry, Faculty of Pharmacy, Zagazig University, Zagazig, 44519 Egypt; 2 Department of Chemistry, Faculty of Science, Zagazig University, Zagazig, 44519 Egypt; 3 Department of Pharmaceutical Chemistry, Faculty of Pharmacy, Sinai University, Kantara Branch, Al-Ismailia, Egypt; 4 Microbiology and Immunology Department, Faculty of Pharmacy, Zagazig University, Zagazig, 44519 Egypt; 5 Department of Analysis and Evaluation, Egyptian Petroleum Research Institute, Cairo, 11727 Egypt

Correction to: *Scientific Reports* 10.1038/s41598-025-11214-4, published online 25 July 2025

The original version of this Article contained an error in the spelling of the author Elsayed M. Mahmoud which was incorrectly given as Mahmoud M. Elsayed.

The original Article has been corrected.

