# Supplementary material for: Dual α-amylase and α-glucosidase inhibition by 1,2,4-triazole derivatives for diabetes treatment
Source: Sci Rep. 2025 Jul 25;15:27172. doi: 10.1038/s41598-025-11214-4 (PMC12297423; doi:10.1038/s41598-025-11214-4)

Tables

Table 1S. Detailed results for α-amylase activity.

Code		conc	log	%inh	T2	T1	ΔT	RFU2	RFU1	ΔRFU	slope	K.Activity
3		100	2	92.3	30	0	30	0.159	0	0.159	0.069	9.2174
		10	1	83.4	30	0	30	0.344	0	0.344	0.069	19.942
		1	0	58.4	30	0	30	0.861	0	0.861	0.069	49.913
		0.1	-1	36.7	30	0	30	1.311	0	1.311	0.069	76
		0.01	-2	19.9	30	0	30	1.659	0	1.659	0.069	96.174
		**EC**		**0**	**30**	**0**	**30**	**2.077**	**0**	**2.077**	**0.069**	**120**
4	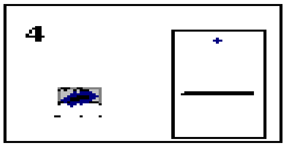 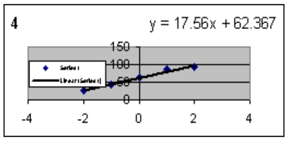	100	2	93.1	30	0	30	0.142	0	0.142	0.069	8.2319
		10	1	85.7	30	0	30	0.297	0	0.297	0.069	17.217
		1	0	62.6	30	0	30	0.775	0	0.775	0.069	44.928
		0.1	-1	44.6	30	0	30	1.146	0	1.146	0.069	66.435
		0.01	-2	25.8	30	0	30	1.535	0	1.535	0.069	88.986
		**EC**		**0**	**30**	**0**	**30**	**2.077**	**0**	**2.077**	**0.069**	**120**
6	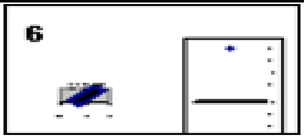 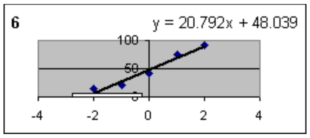	100	2	91.3	30	0	30	0.181	0	0.181	0.069	10.493
		10	1	74.1	30	0	30	0.537	0	0.537	0.069	31.13
		1	0	40.6	30	0	30	1.229	0	1.229	0.069	71.246
		0.1	-1	19.9	30	0	30	1.659	0	1.659	0.069	96.174
		0.01	-2	14.4	30	0	30	1.772	0	1.772	0.069	102.72
		**EC**		**0**	**30**	**0**	**30**	**2.077**	**0**	**2.077**	**0.069**	**120**
8	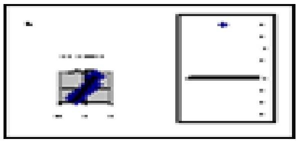	100	2	88.7	30	0	30	0.233	0	0.233	0.069	13.507
		10	1	63.1	30	0	30	0.764	0	0.764	0.069	44.29
		1	0	41.6	30	0	30	1.208	0	1.208	0.069	70.029
		0.1	-1	29.3	30	0	30	1.463	0	1.463	0.069	84.812
		0.01	-2	12	30	0	30	1.822	0	1.822	0.069	105.62

	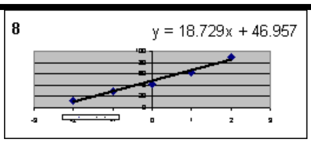	EC		**0**	**30**	**0**	**30**	**2.077**	**0**	**2.077**	**0.069**	**120**
9	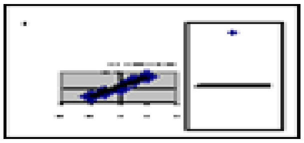 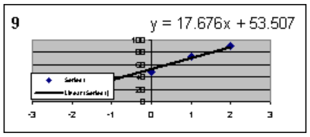	100	2	90	30	0	30	0.207	0	0.207	0.069	12
		10	1	73.3	30	0	30	0.552	0	0.552	0.069	32
		1	0	48.3	30	0	30	1.071	0	1.071	0.069	62.087
		0.1	-1	35.3	30	0	30	1.339	0	1.339	0.069	77.623
		0.01	-2	20.6	30	0	30	1.643	0	1.643	0.069	95.246
		EC		**0**	**30**	**0**	**30**	**2.077**	**0**	**2.077**	**0.069**	**120**
10	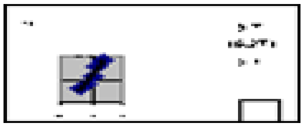 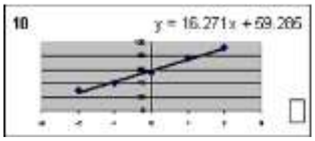	100	2	92.3	30	0	30	0.159	0	0.159	0.069	9.2174
		10	1	77.6	30	0	30	0.463	0	0.463	0.069	26.841
		1	0	56.1	30	0	30	0.909	0	0.909	0.069	52.696
		0.1	-1	41.2	30	0	30	1.217	0	1.217	0.069	70.551
		0.01	-2	29.2	30	0	30	1.466	0	1.466	0.069	84.986
		EC		**0**	**30**	**0**	**30**	**2.077**	**0**	**2.077**	**0.069**	**120**
Acar.	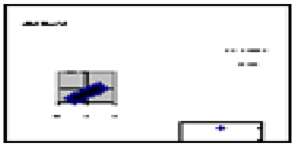 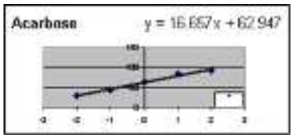	100	2	93.4	30	0	30	0.136	0	0.136	0.069	7.8841
		10	1	84.2	30	0	30	0.328	0	0.328	0.069	19.014
		1	0	62.8	30	0	30	0.771	0	0.771	0.069	44.696
		0.1	-1	44.3	30	0	30	1.152	0	1.152	0.069	66.783
		0.01	-2	30	30	0	30	1.448	0	1.448	0.069	83.942
		EC		**0**	**30**	**0**	**30**	**2.077**	**0**	**2.077**	**0.069**	**120**

Table 2S. Detailed results for α-glucosidase activity.

Code		conc	log	%inh	T2	T1	ΔT	RFU2	RFU1	ΔRFU	slope	K.Activity
3	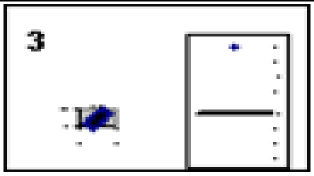	1000	3	77	30	0	30	0.441	0	0.441	0.064	27.563
		500	2.7	58.5	30	0	30	0.796	0	0.796	0.064	49.75
		250	2.4	40.9	30	0	30	1.135	0	1.135	0.064	70.938
		125	2.1	30	30	0	30	1.344	0	1.344	0.064	84
		63	1.8	15.7	30	0	30	1.618	0	1.618	0.064	101.13
	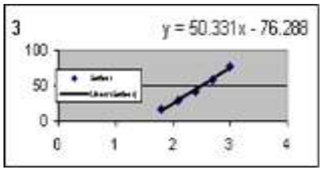	**EC**		**0**	**30**	**0**	**30**	**1.907**	**0**	**1.907**	**0.064**	**120**
4	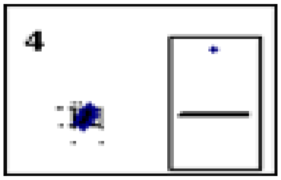	1000	3	82.4	30	0	30	0.337	0	0.337	0.064	21.063
		500	2.7	63.6	30	0	30	0.699	0	0.699	0.064	43.688
		250	2.4	50.9	30	0	30	0.943	0	0.943	0.064	58.938
		125	2.1	36.6	30	0	30	1.217	0	1.217	0.064	76.063
		63	1.8	19.9	30	0	30	1.538	0	1.538	0.064	96.125
	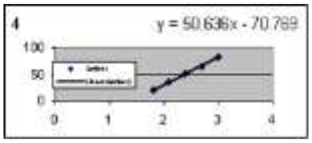	**EC**		**0**	**30**	**0**	**30**	**1.907**	**0**	**1.907**	**0.064**	**120**
6	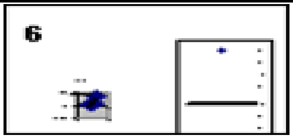	1000	3	69.2	30	0	30	0.592	0	0.592	0.064	37
		500	2.7	28.9	30	0	30	1.366	0	1.366	0.064	85.375
	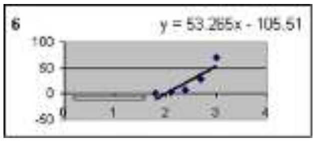	250	2.4	7.55	30	0	30	1.775	0	1.775	0.064	110.94
		125	2.1	4.01	30	0	30	1.843	0	1.843	0.064	115.19
		63	1.8	1.67	30	0	30	1.888	0	1.888	0.064	118
		**EC**		**0**	**30**	**0**	**30**	**1.907**	**0**	**1.907**	**0.064**	**120**
8	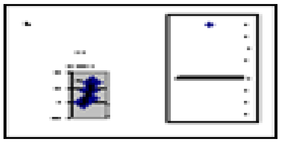	1000	3	67.3	30	0	30	0.628	0	0.628	0.064	39.25
		500	2.7	38.9	30	0	30	1.173	0	1.173	0.064	73.313
		250	2.4	18.4	30	0	30	1.567	0	1.567	0.064	97.938
	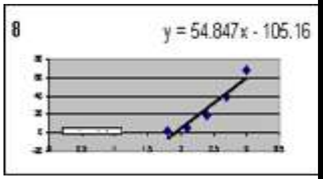	125	2.1	5.94	30	0	30	1.806	0	1.806	0.064	112.88
		63	1.8	1.46	30	0	30	1.892	0	1.892	0.064	118.25
		**EC**		**0**	**30**	**0**	**30**	**1.907**	**0**	**1.907**	**0.064**	**120**
9		1000	3	68.2	30	0	30	0.611	0	0.611	0.064	38.188

	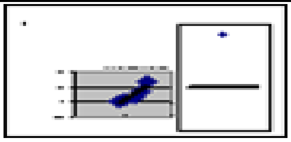	500	2.7	31.9	30	0	30	1.308	0	1.308	0.064	81.75
		250	2.4	14	30	0	30	1.652	0	1.652	0.064	103.25
	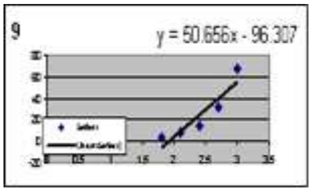	125	2.1	7.76	30	0	30	1.771	0	1.771	0.064	110.69
		63	1.8	4.22	30	0	30	1.839	0	1.839	0.064	114.94
		**EC**		**0**	**30**	**0**	**30**	**1.907**	**0**	**1.907**	**0.064**	**120**
10	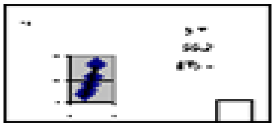	1000	3	82.8	30	0	30	0.331	0	0.331	0.064	20.688
		500	2.7	58	30	0	30	0.806	0	0.806	0.064	50.375
		250	2.4	39.9	30	0	30	1.153	0	1.153	0.064	72.063
	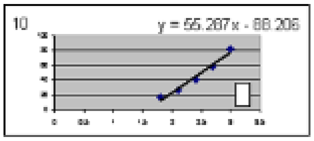	125	2.1	25.1	30	0	30	1.439	0	1.439	0.064	89.938
		63	1.8	16.3	30	0	30	1.608	0	1.608	0.064	100.5
		**EC**		**0**	**30**	**0**	**30**	**1.907**	**0**	**1.907**	**0.064**	**120**
Acar.	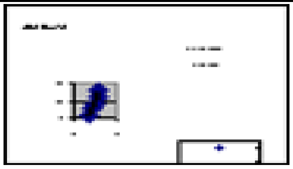	1000	3	76.8	30	0	30	0.446	0	0.446	0.064	27.875
		500	2.7	58.6	30	0	30	0.794	0	0.794	0.064	49.625
		250	2.4	40.6	30	0	30	1.141	0	1.141	0.064	71.313
		125	2.1	28	30	0	30	1.383	0	1.383	0.064	86.438
		63	1.8	8.91	30	0	30	1.749	0	1.749	0.064	109.31
	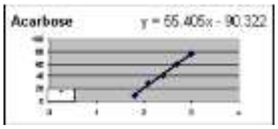	**EC**		**0**	**30**	**0**	**30**	**1.907**	**0**	**1.907**	**0.064**	**120**

Table (3S): The binding scores & RMSD values of the promising compounds as Antidiabetic to the target enzyme α-amylase and α-glucosidase

COMPOUNDS	α-amylase								α-glucosidase			
	4X9Y Wild-Type Human Pancreatic Alpha-Amylase		2QV4 Human pancreatic alpha-amylase complexed with nitrite and acarbose		5E0F Human pancreatic alpha-amylase in complex with mini-montbretin A		1OSE Porcine pancreatic alpha-amylase complexed with acarbose [Sus scrofa]		3A4A Crystal structure of isomaltase from Saccharomyces cerevisiae [Saccharomyces cerevisiae]		3AJ7 Crystal Structure of isomaltase from Saccharomyces cerevisiae [Saccharomyces cerevisiae]	
	Score (Kcal/mol)	RMSD	Score (Kcal/mol)	RMSD	Score (Kcal/mol)	RMSD	Score (Kcal/mol)	RMSD	Score (Kcal/mol)	RMSD	Score (Kcal/mol)	RMSD
1	-7.369	1.95	-7.283	1.95	-7.527	1.35	-6.853	1.54	-7.959	1.05	-8.422	1.58
2	-7.541	1.89	-7.295	1.40	-7.554	1.51	-7.117	1.82	-7.894	1.76	-7.687	1.74
3	-7.441	1.30	-7.424	1.33	-7.351	1.86	-7.322	1.34	-7.854	1.99	-8.291	1.45
4	-7.777	1.43	-7.758	1.83	-8.090	1.96	-7.487	1.99	-8.655	1.26	-8.116	1.37
5	-7.313	1.52	-7.567	1.85	-7.542	1.95	-7.211	1.79	-8.230	0.94	-8.023	1.15
6	-7.738	1.51	-7.396	1.39	-7.547	1.56	-7.635	1.52	-8.108	1.94	-8.248	1.87
7	-7.661	1.93	-7.44	1.69	-6.986	1.31	-7.306	1.35	-8.311	1.51	-8.208	1.39
8	-7.751	1.77	-7.924	0.96	-7.933	1.50	-7.790	1.94	-8.927	1.79	-8.948	1.89
9	-7.588	1.70	-7.465	0.86	-7.344	1.70	-6.994	1.88	-8.186	1.80	-8.158	1.37
10	-7.833	1.75	-7.682	1.84	-7.089	1.83	-7.335	1.87	-7.965	1.53	-8.014	0.95
forxiga	-6.956	1.36	-6.780	0.83	-6.706	1.43	-6.868	1.59	-7.893	1.43	-7.893	1.43
Acarbose	-8.369	3.02	-8.766	1.926	-8.533	1.81	-7.501	1.96	-10.426	1.42	-10.063	1.89

Spectral Analysis

IR-Chart of compound **1**

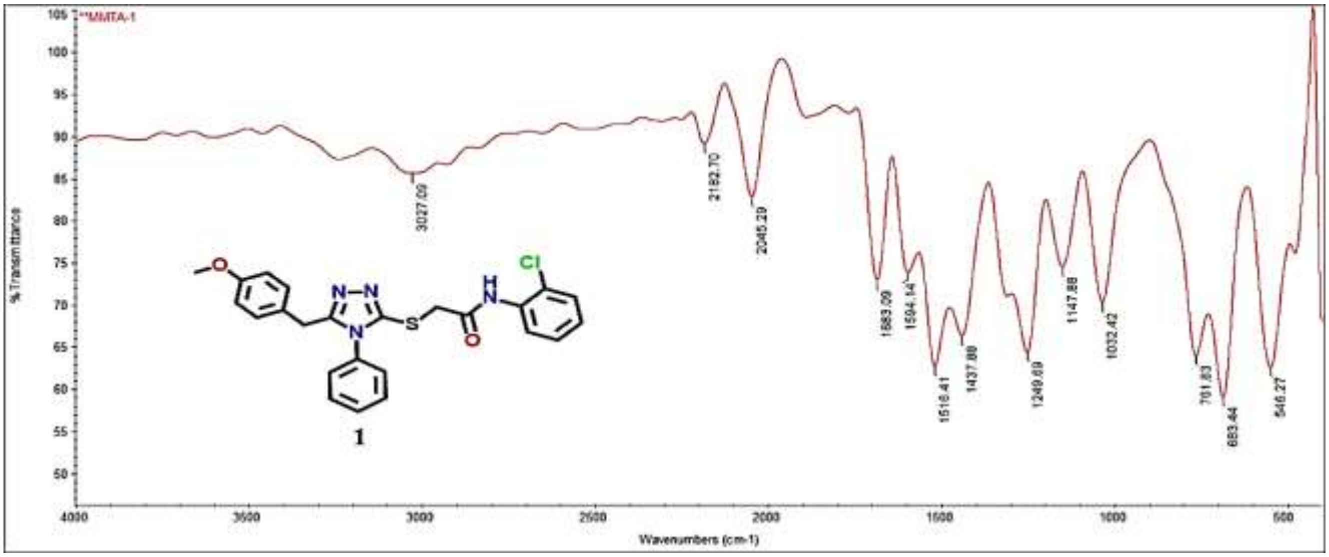

^{1}H NMR spectrum of compound **1**

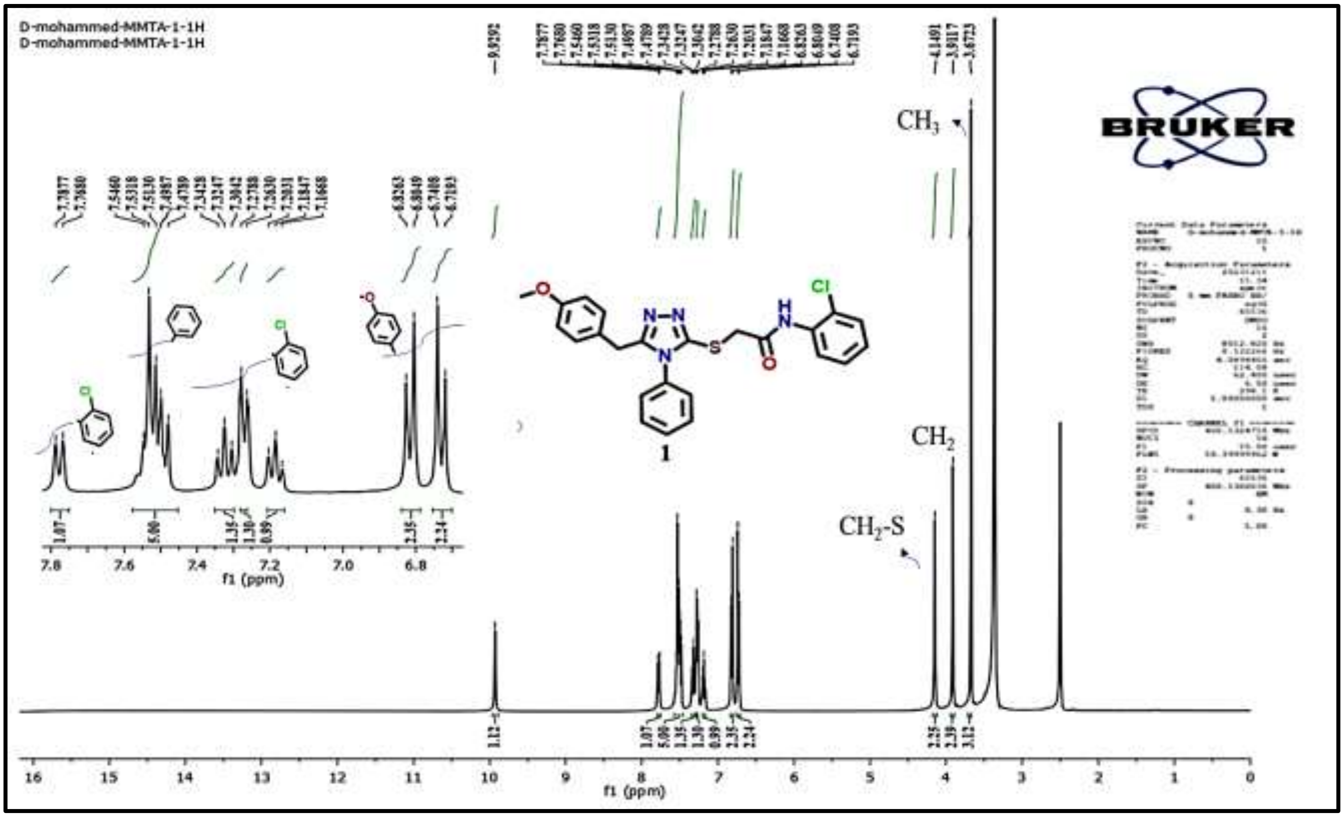

^{13}C NMR spectrum of compound **1**

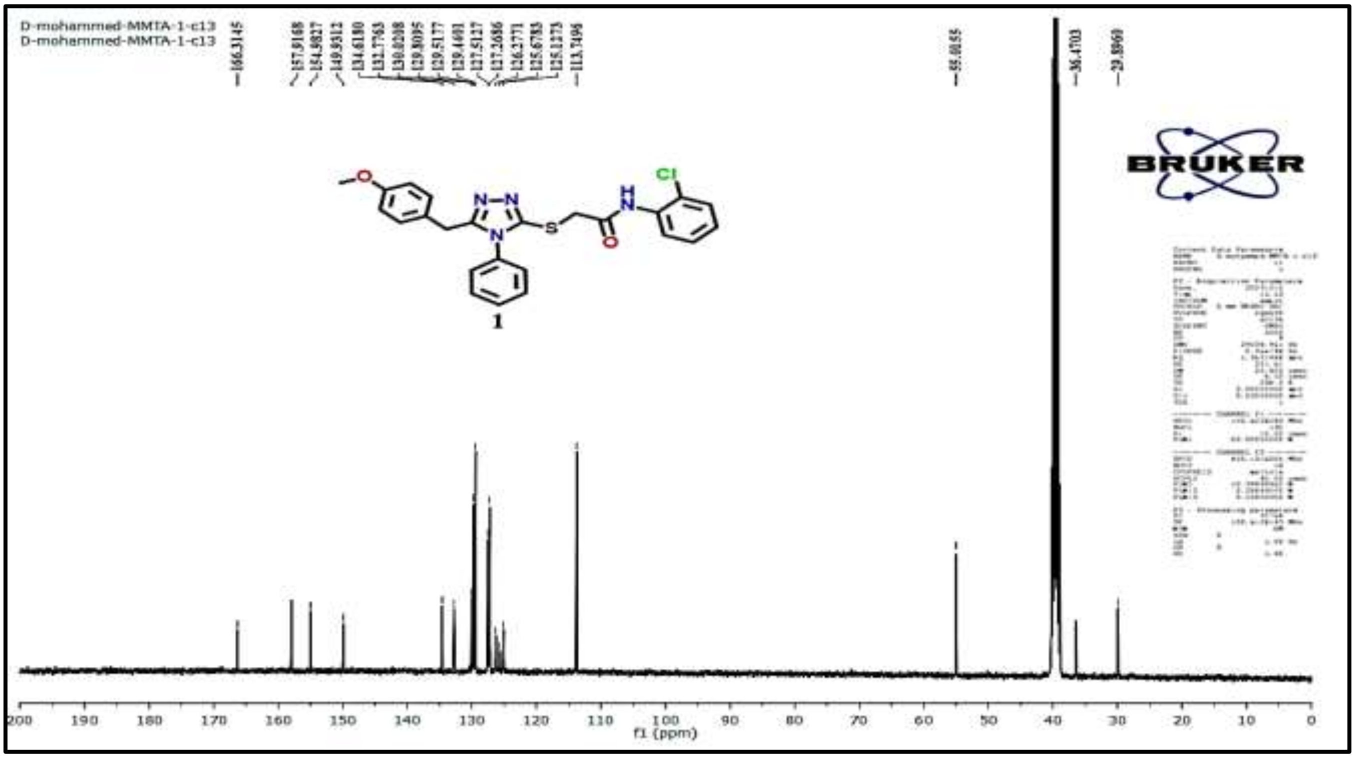

IR-Chart of compound **2**

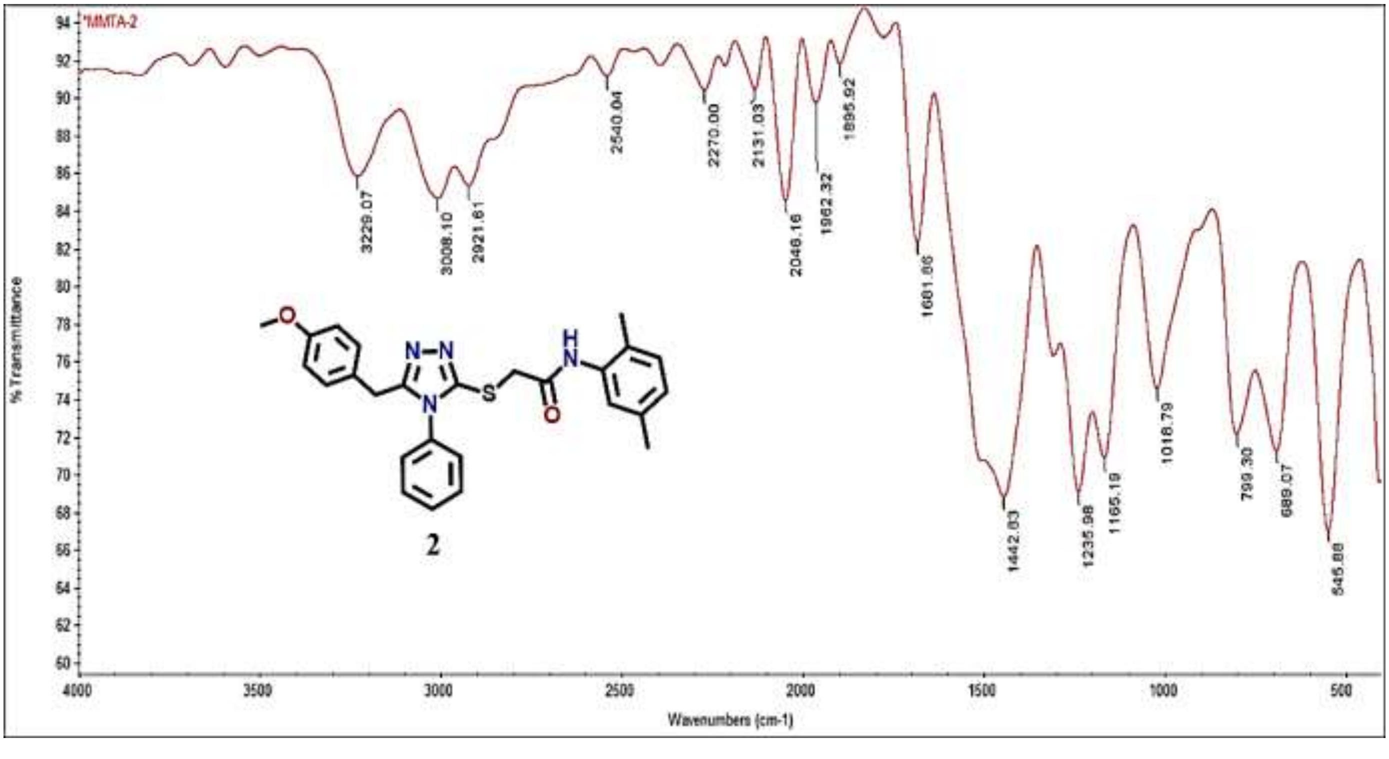

^{1}H NMR spectrum of compound **2**

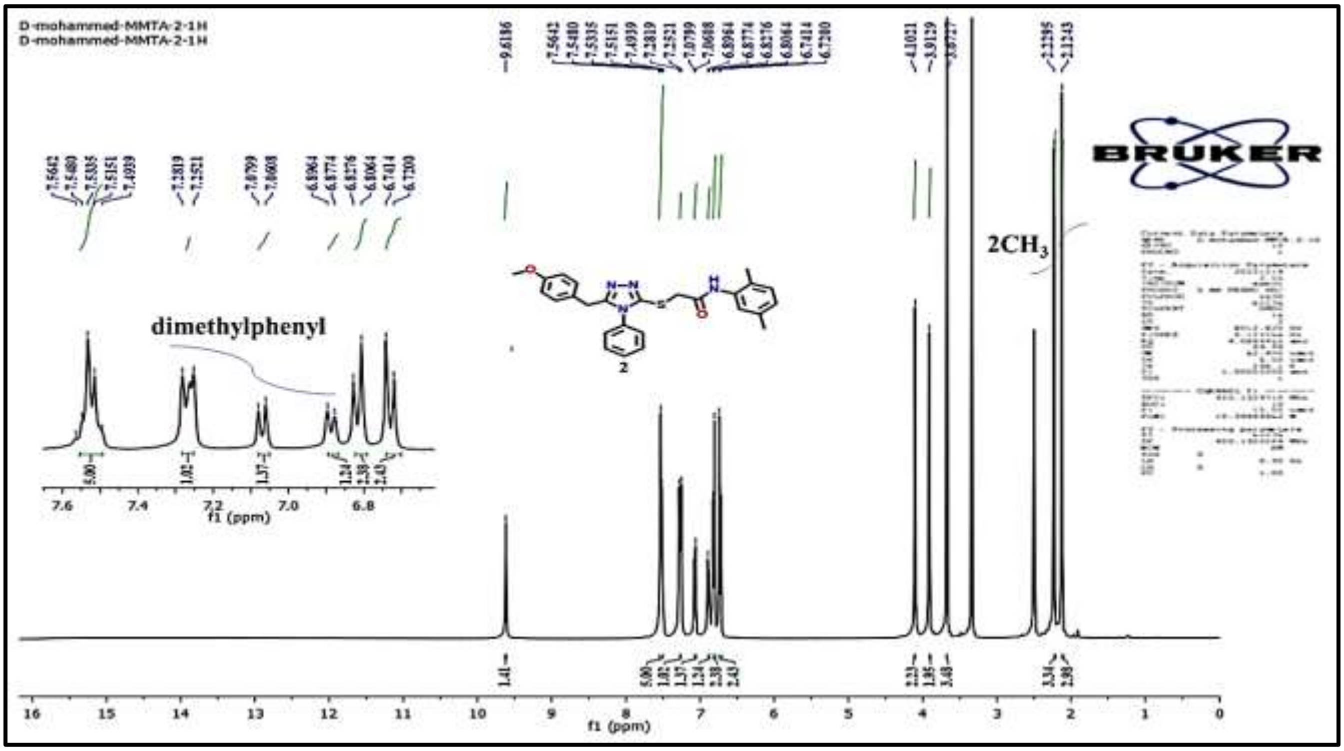

^{13}C NMR spectrum of compound **2**

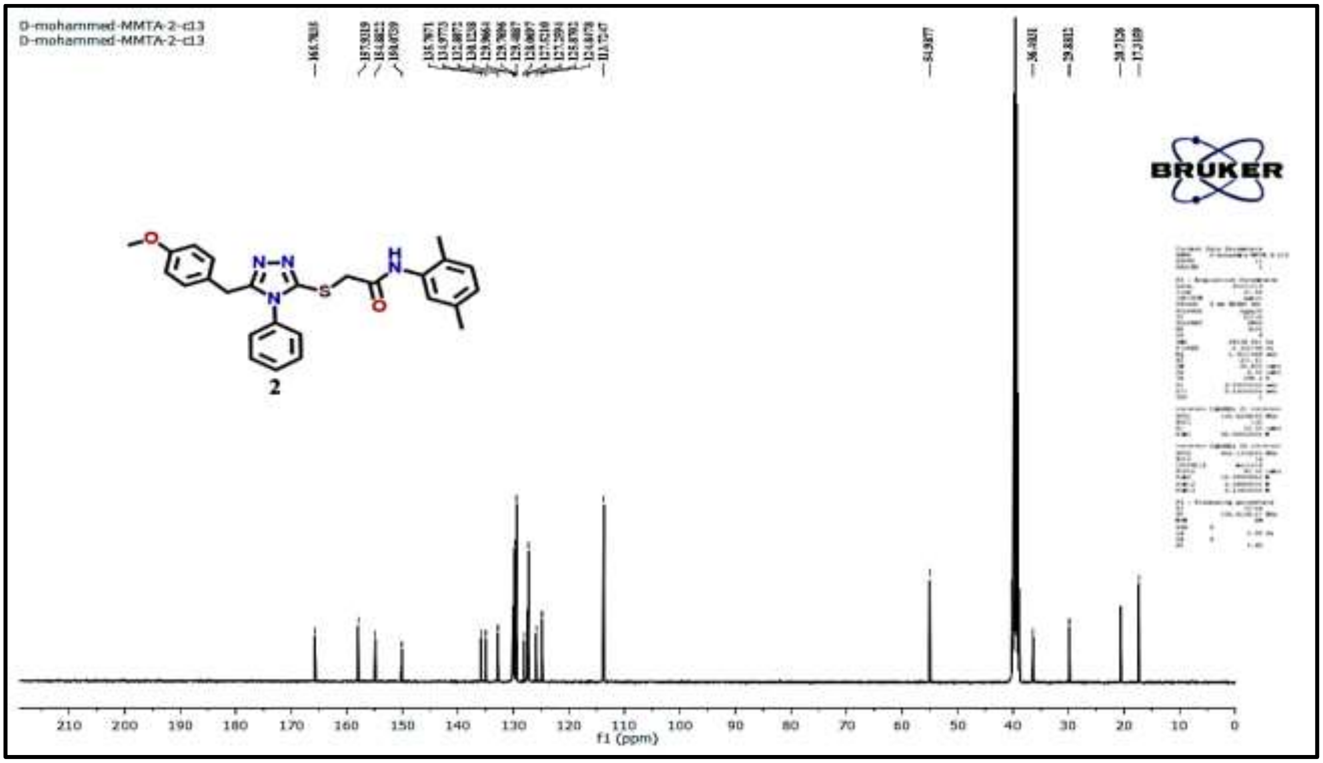

IR-Chart of compound **3**

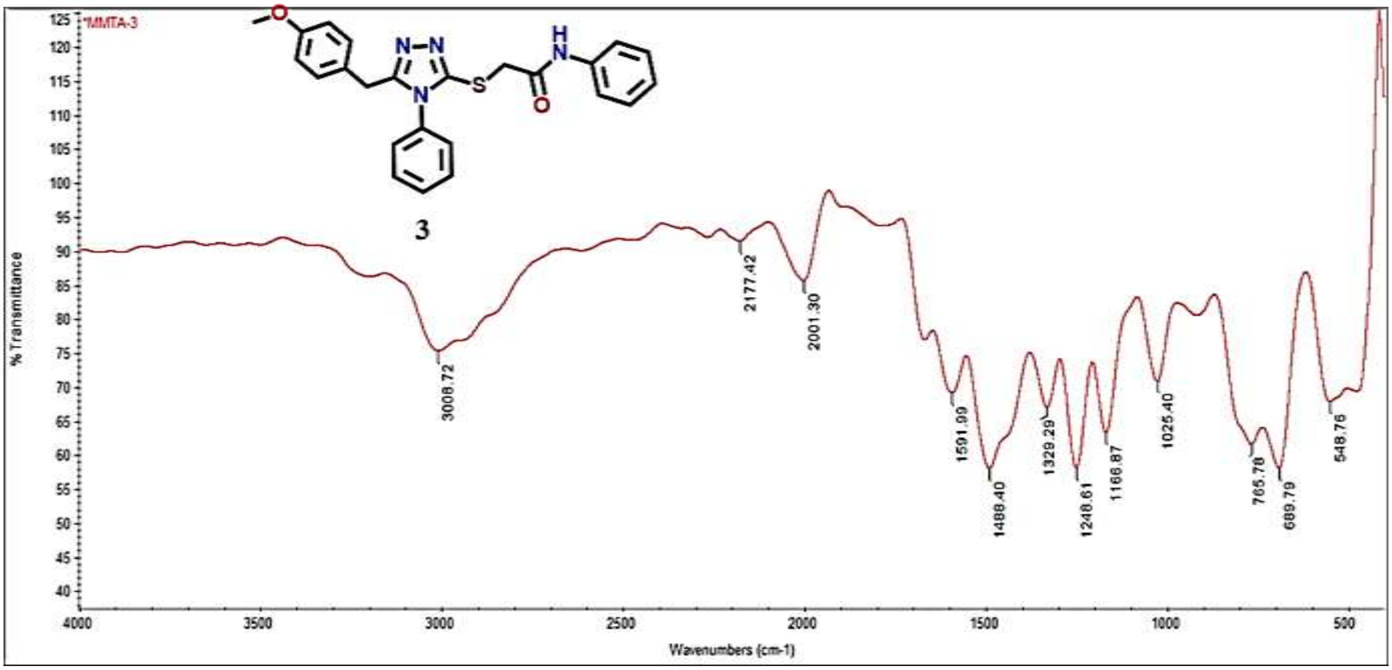

^{1}H NMR spectrum of compound **3**

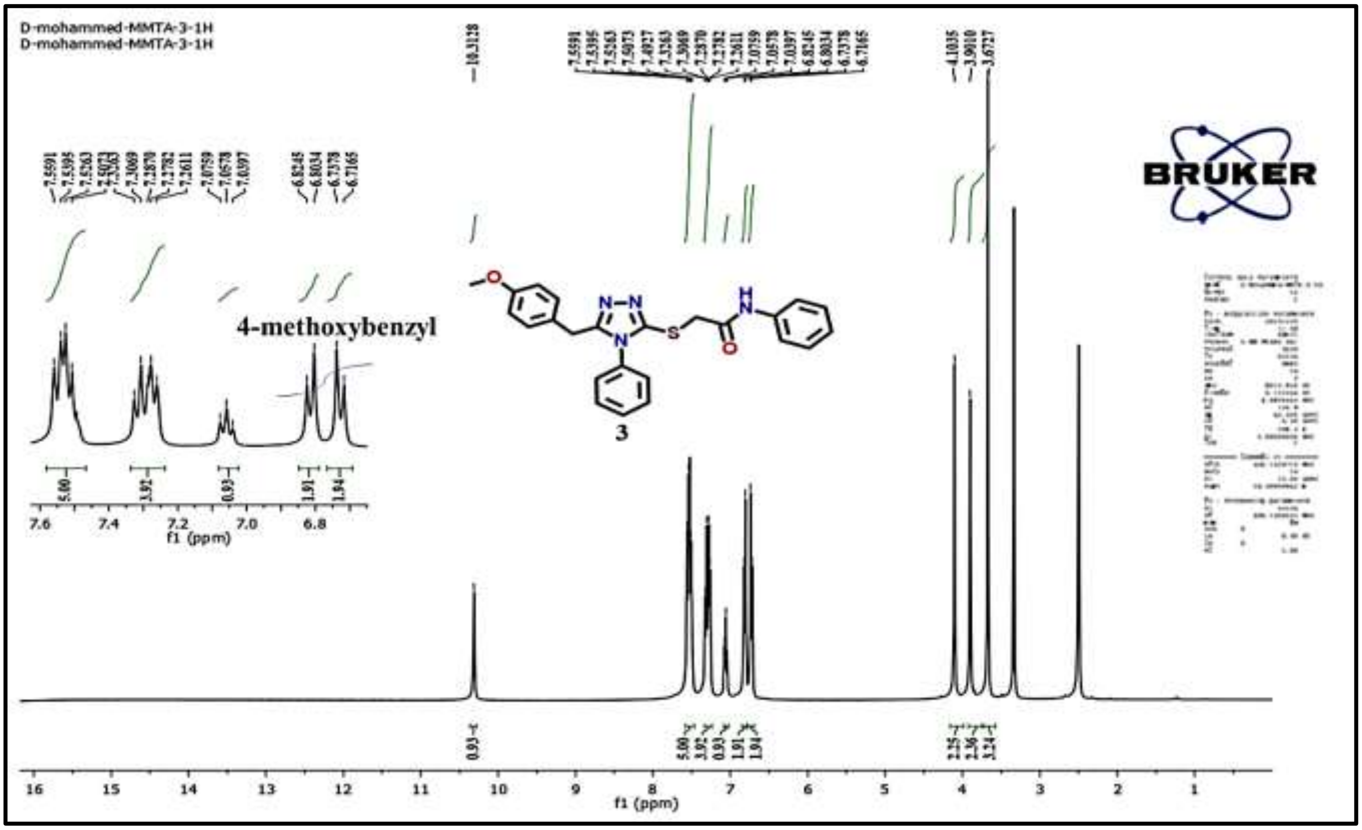

^{13}C NMR spectrum of compound **3**

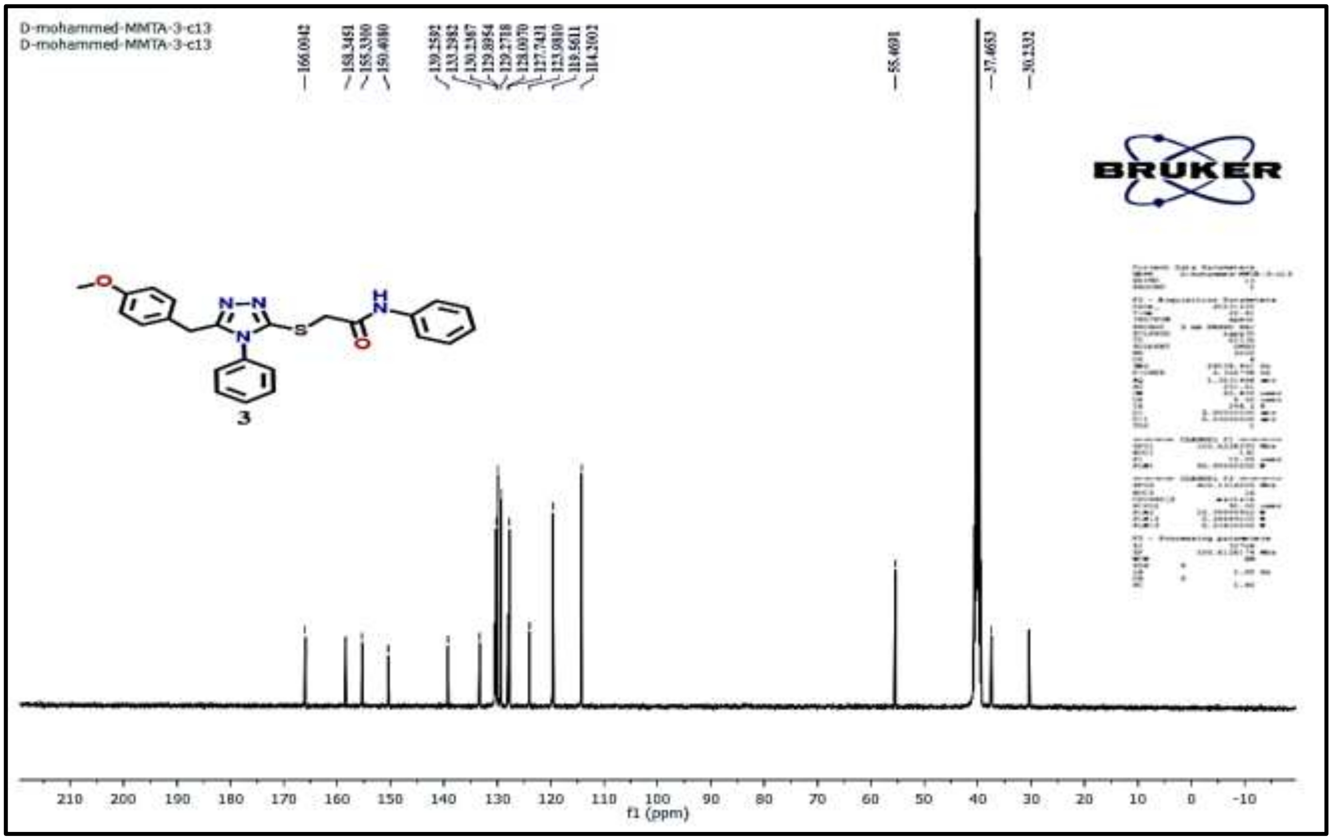

IR-Chart of compound **4**

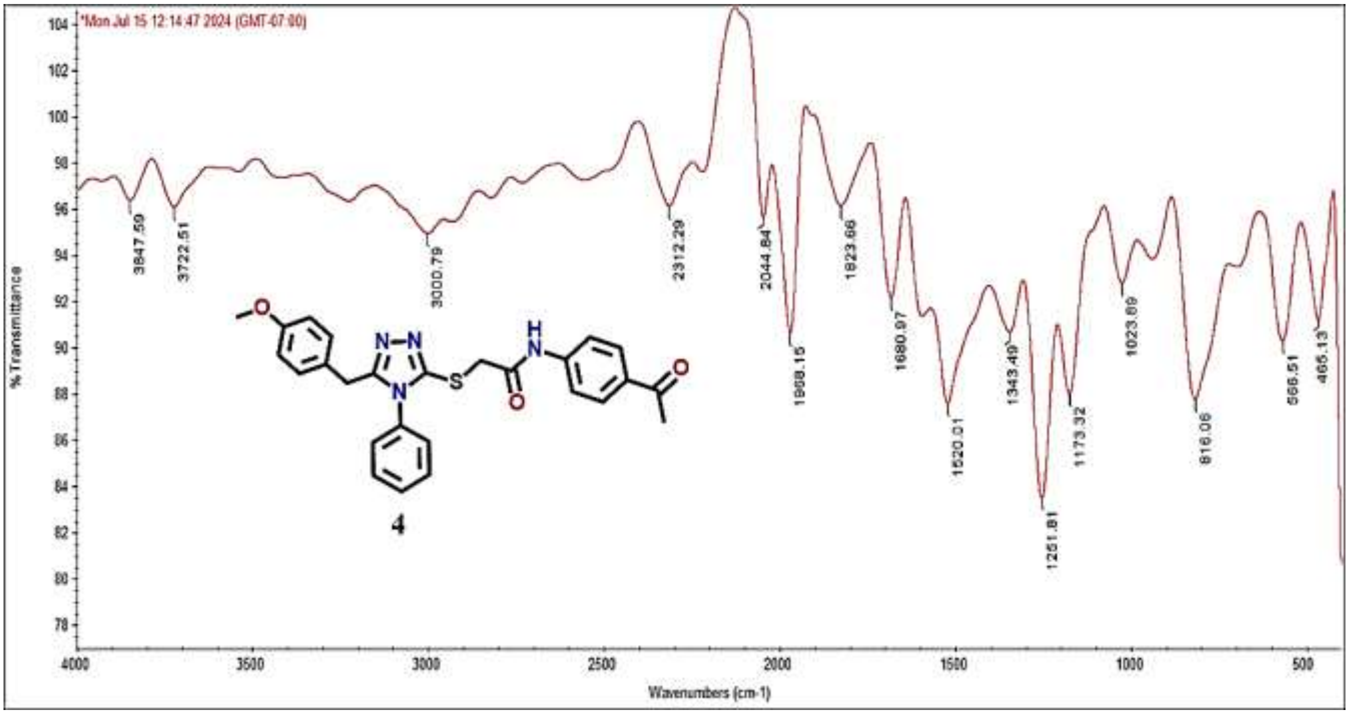

^{1}H NMR spectrum of compound **4**

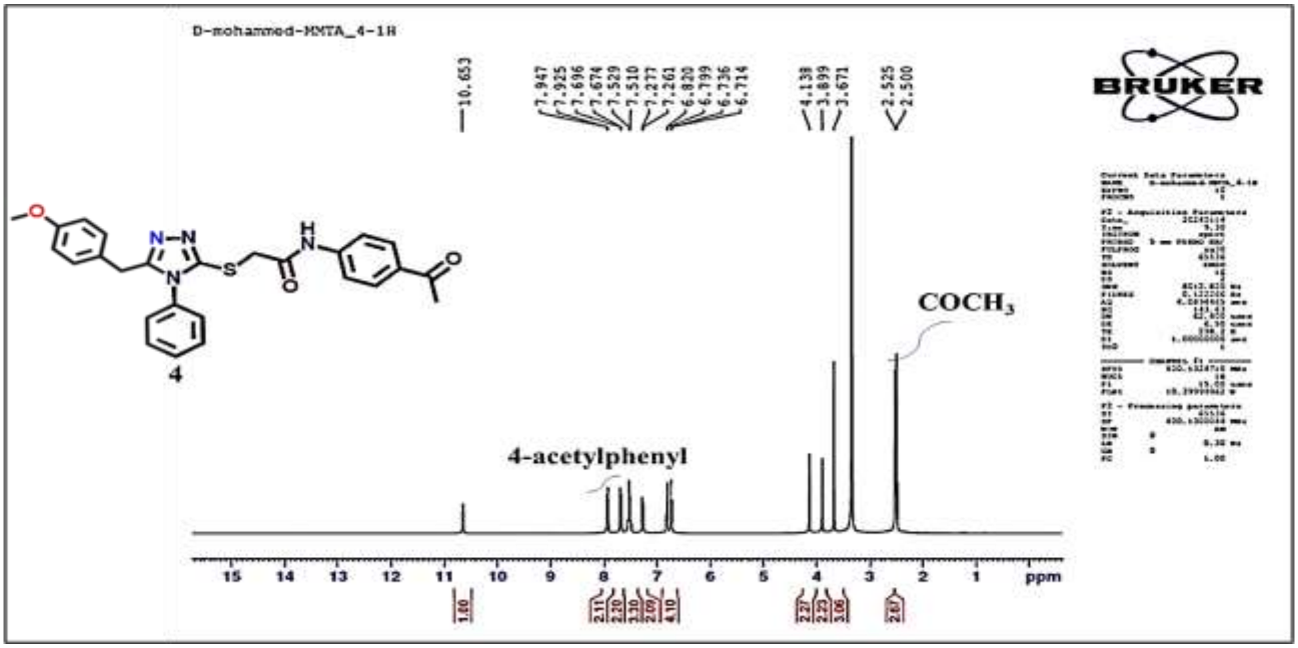

^{13}C NMR spectrum of compound **4**

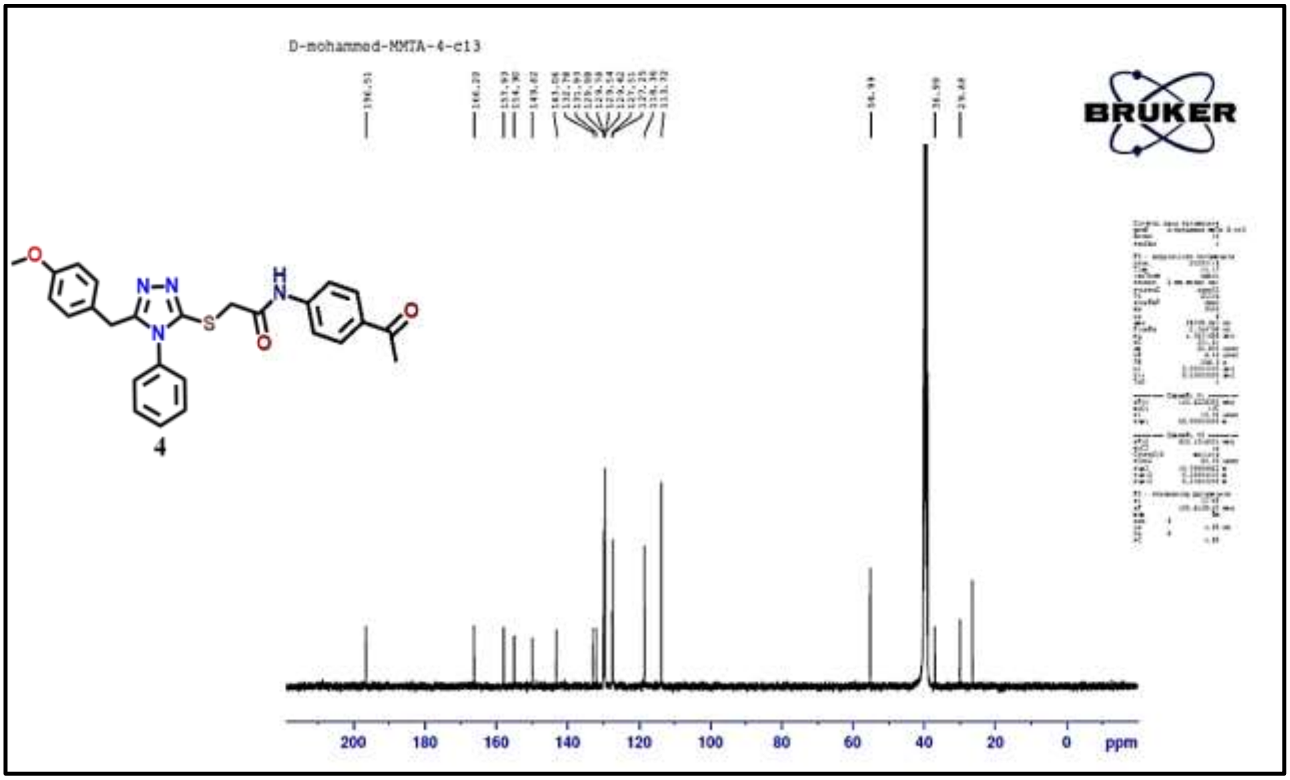

IR-Chart of compound **5**

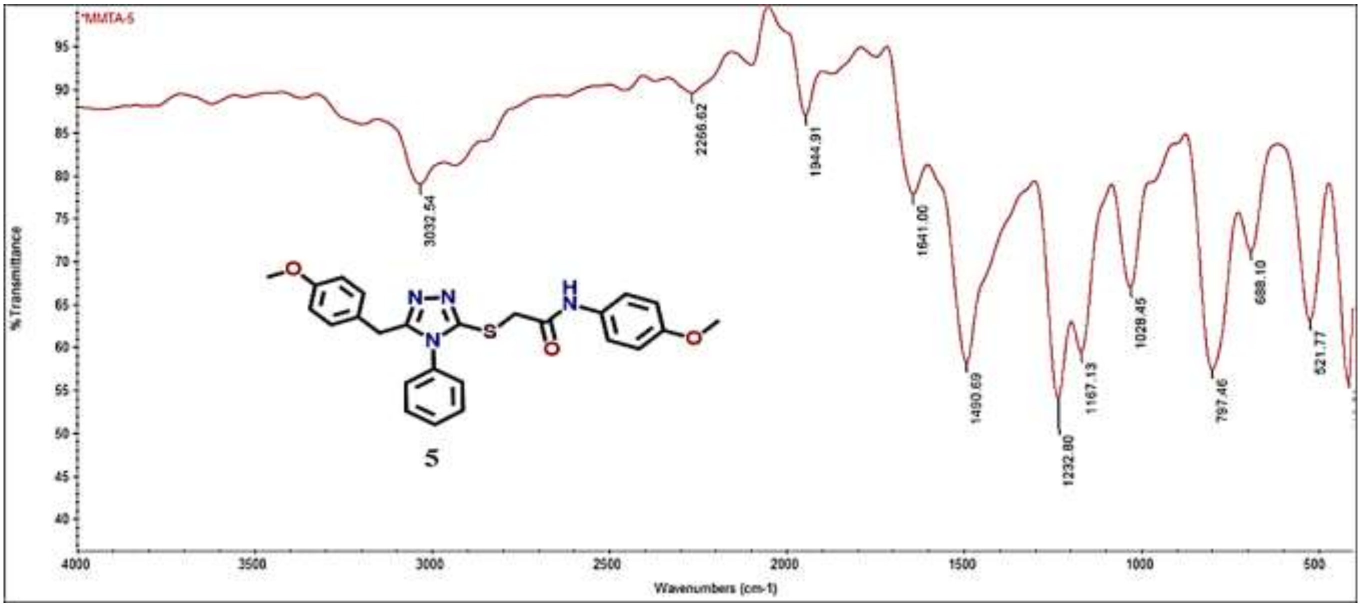

^{1}H NMR spectrum of compound **5**

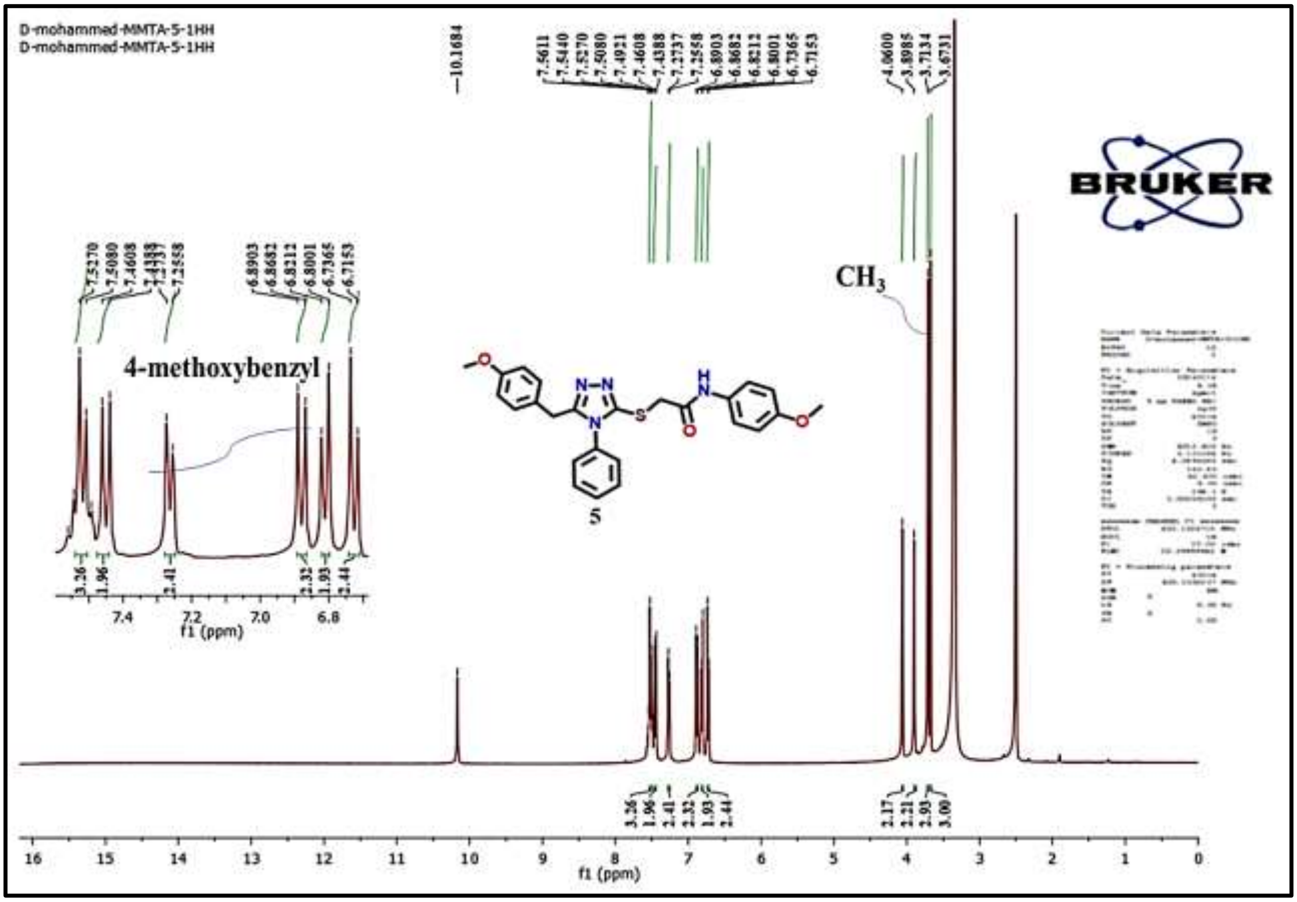

^{13}C NMR spectrum of compound **5**

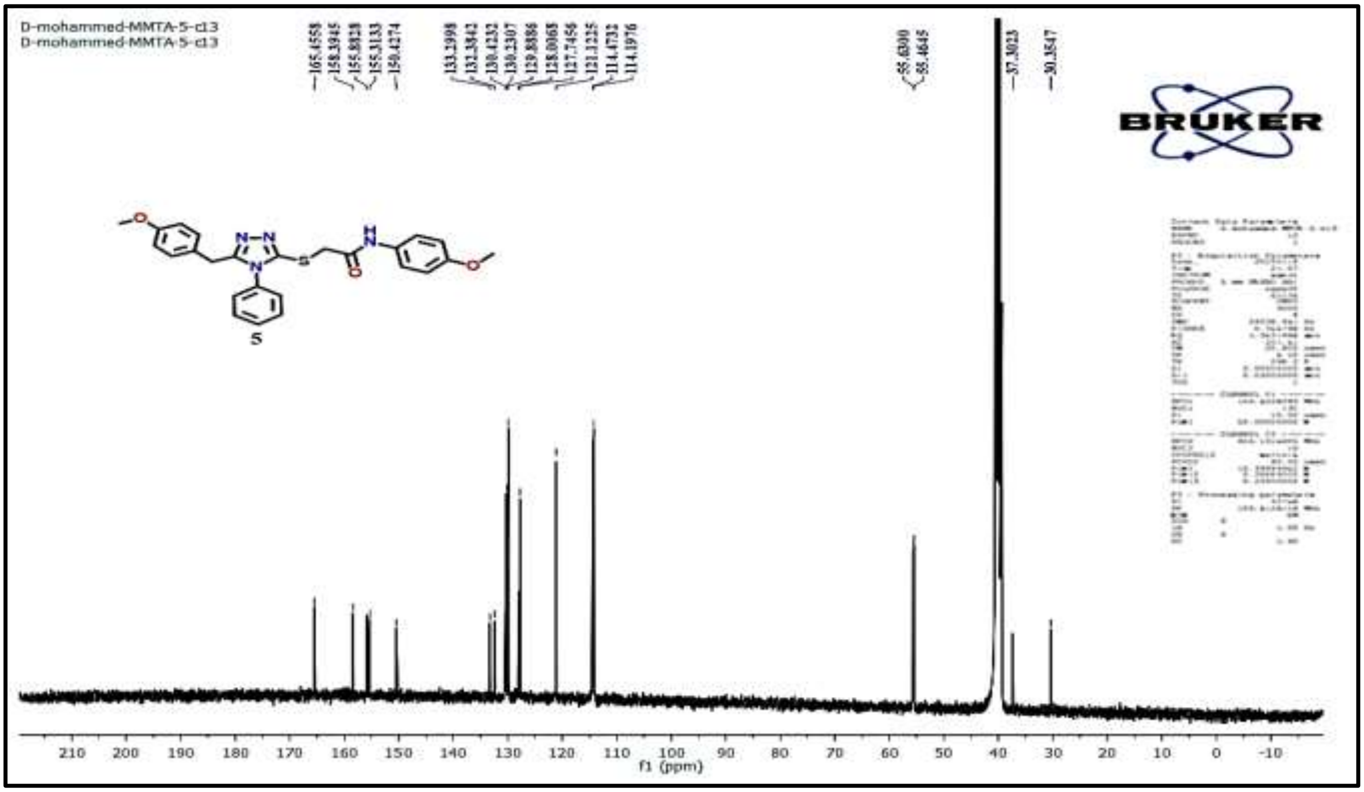

IR-Chart of compound **6**

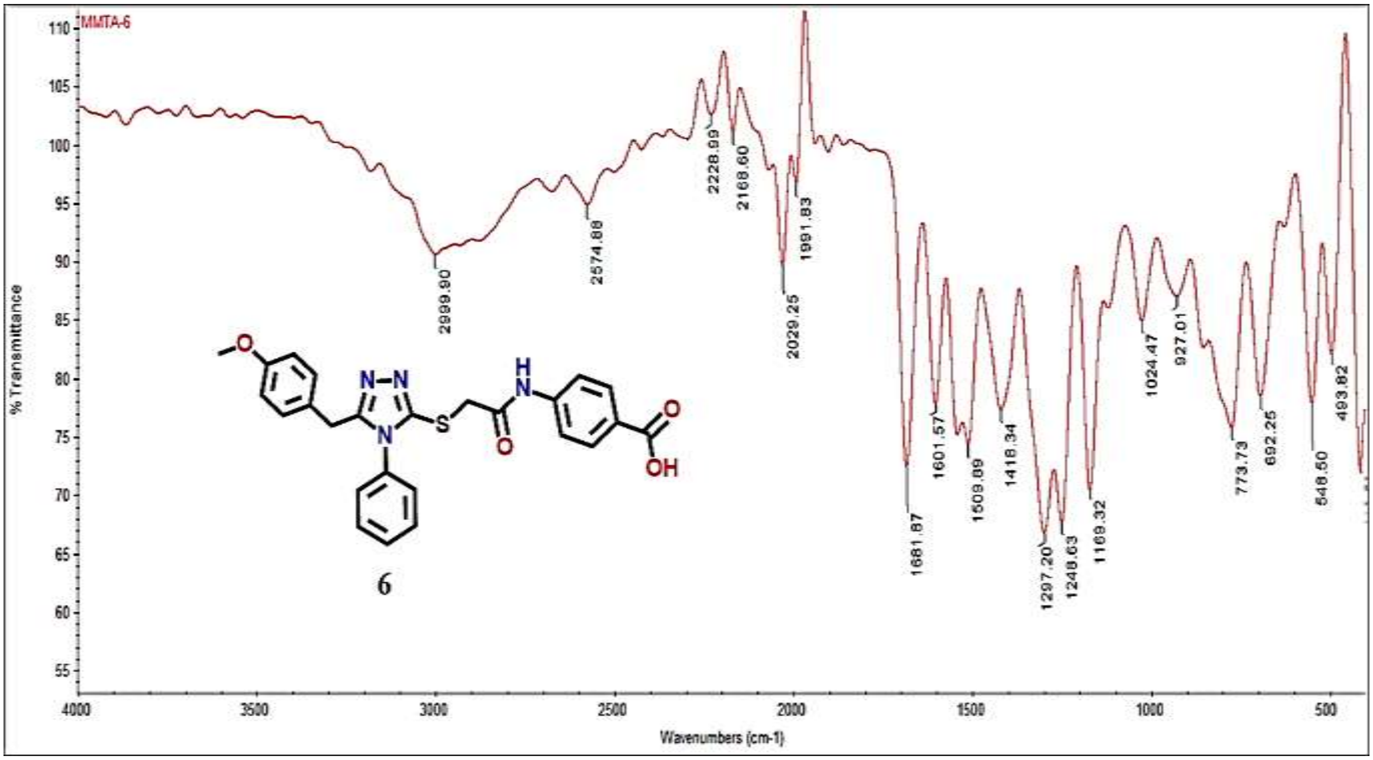

^{1}H NMR spectrum of compound **6**

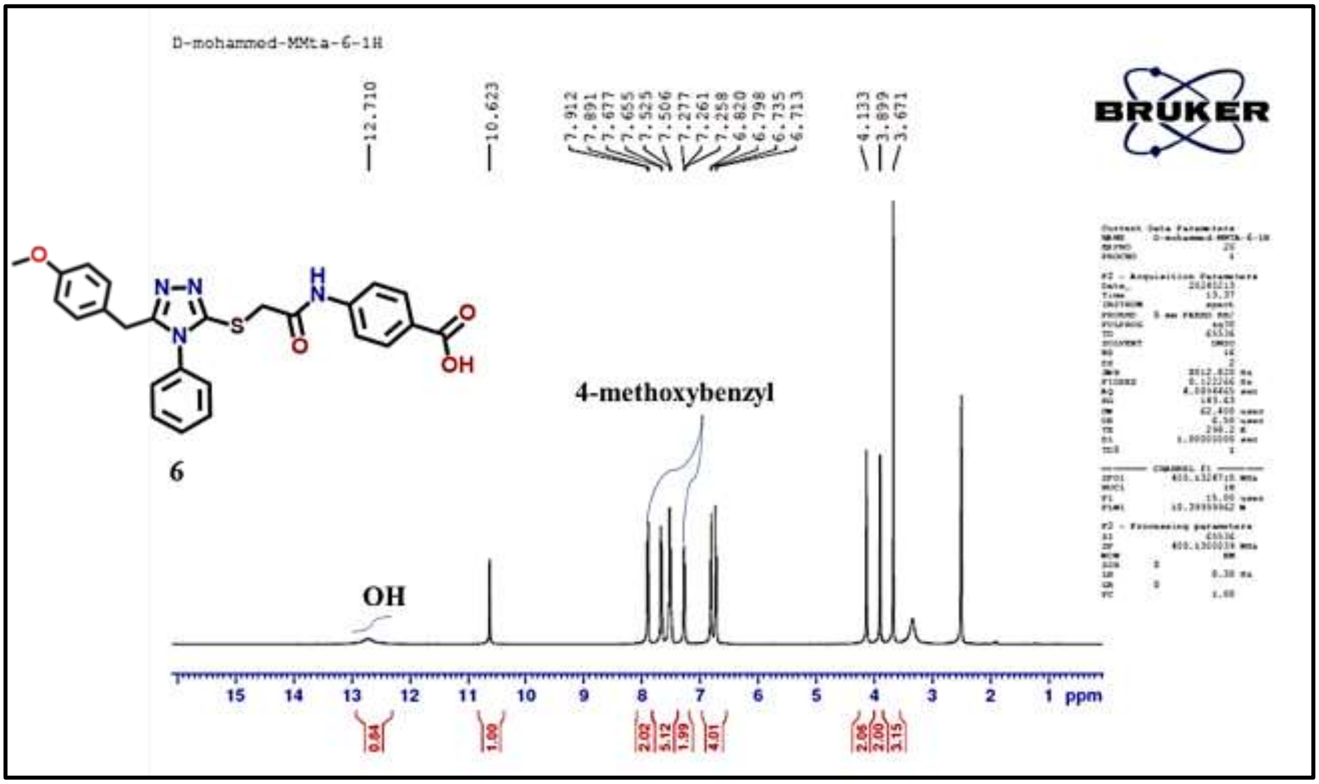

^{13}C NMR spectrum of compound **6**

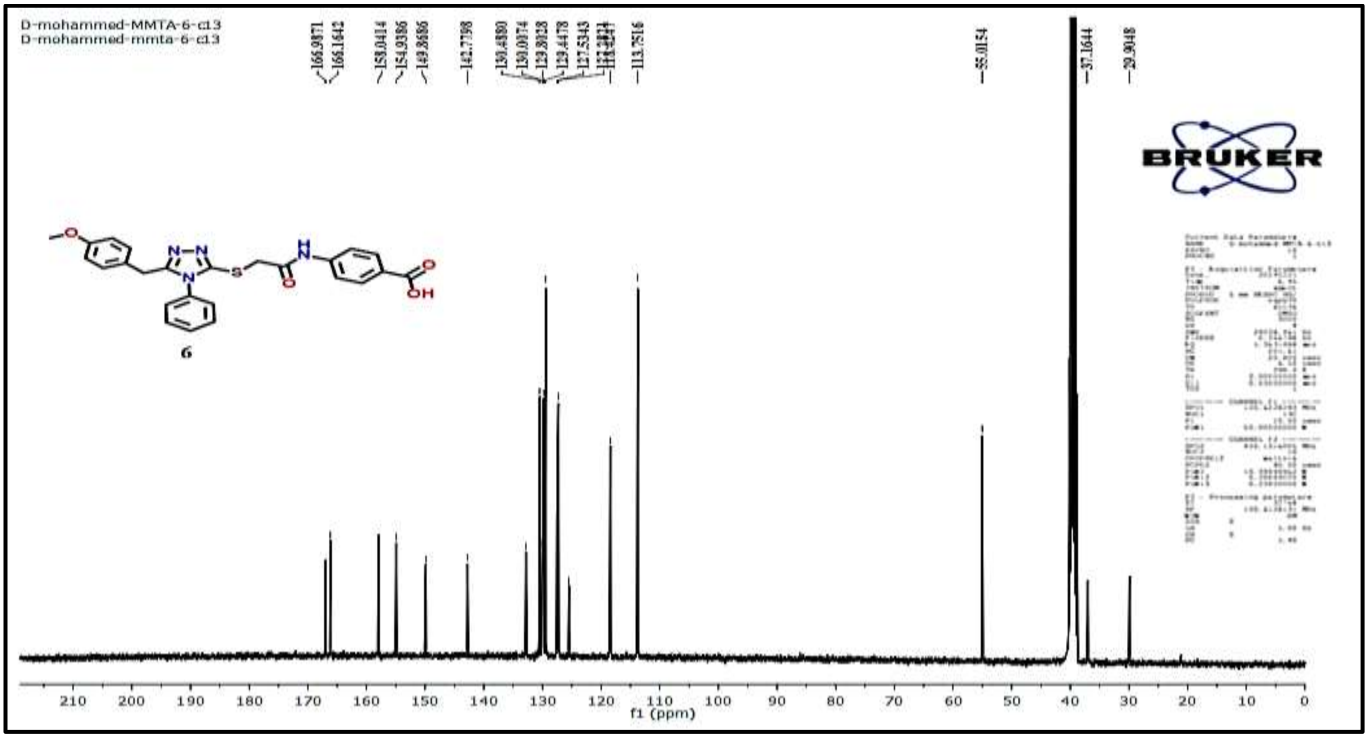

IR-Chart of compound **7**

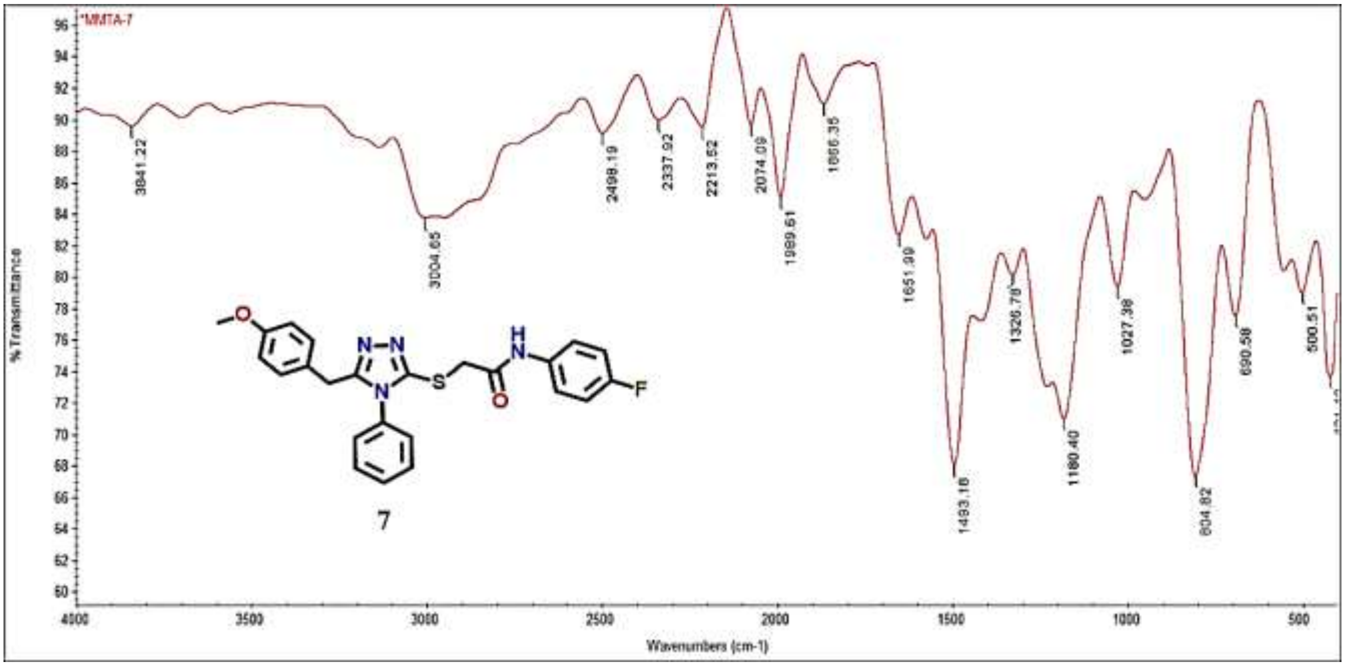

^{1}H NMR spectrum of compound **7**

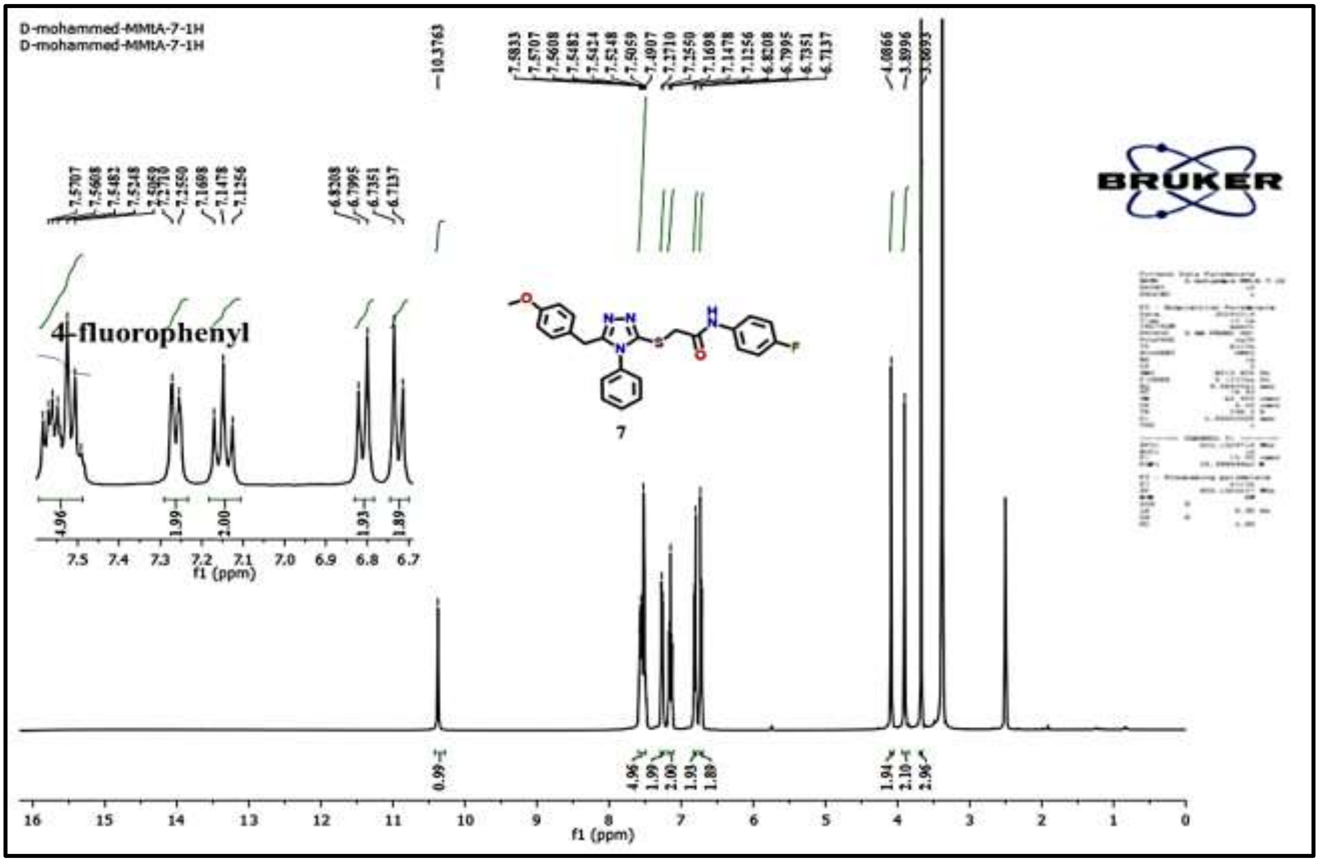

^{13}C NMR spectrum of compound **7**

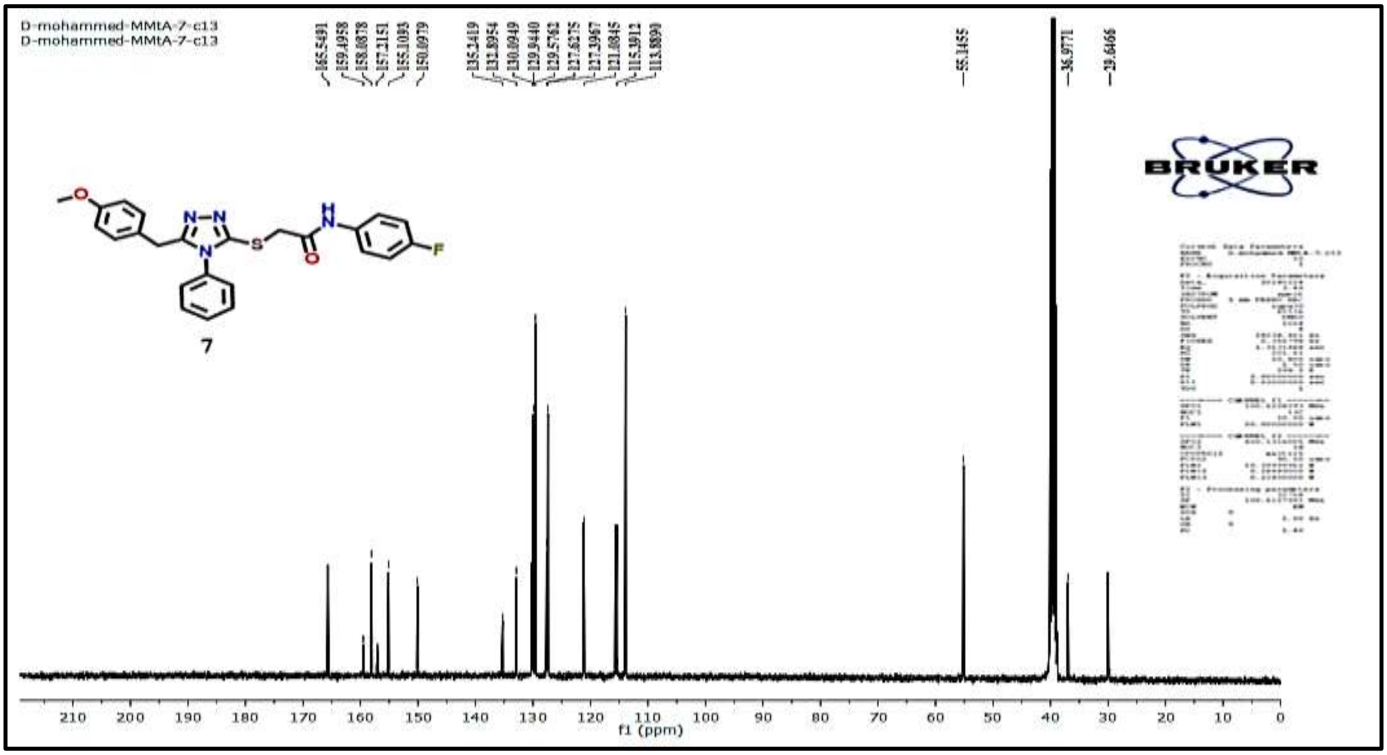

IR-Chart of compound **8**

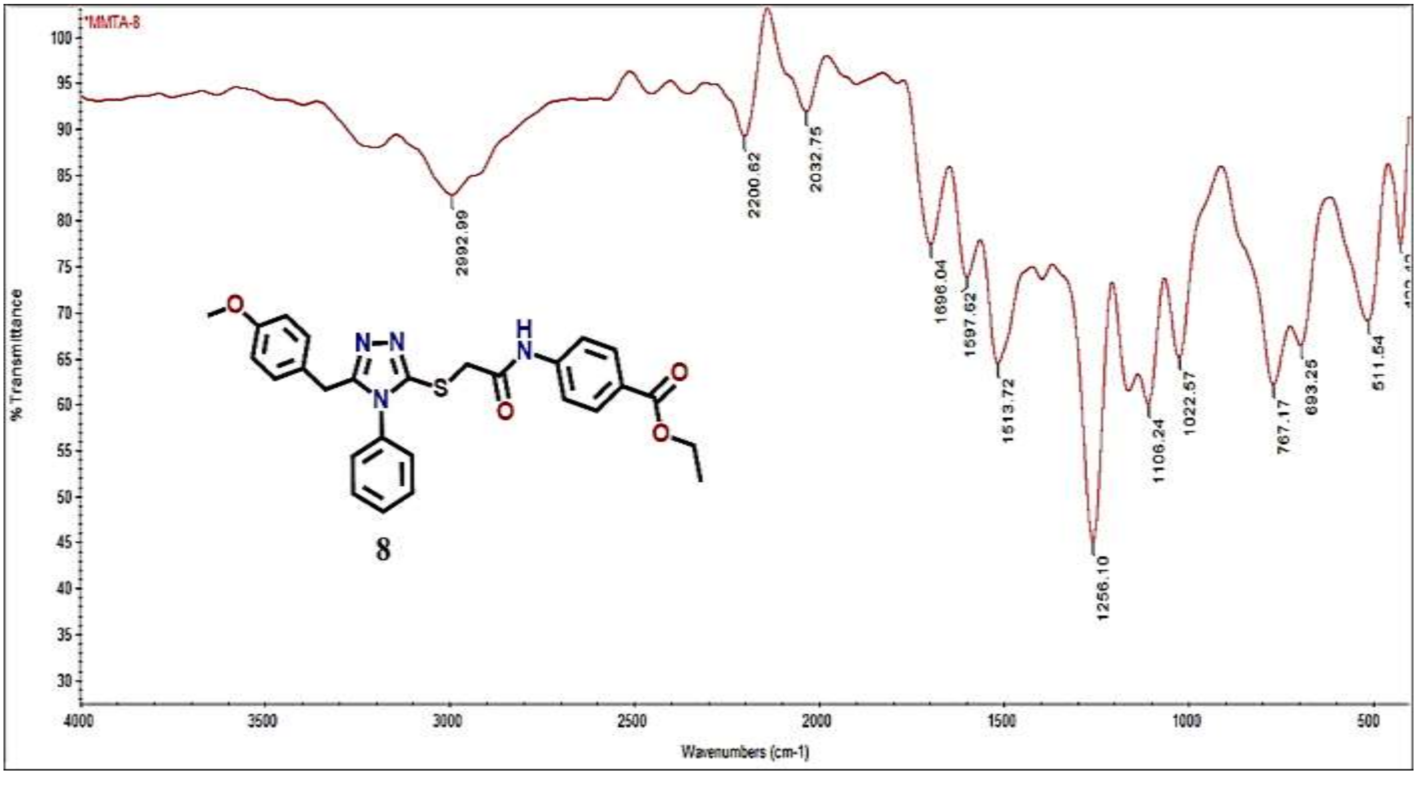

^{1}H NMR spectrum of compound **8**

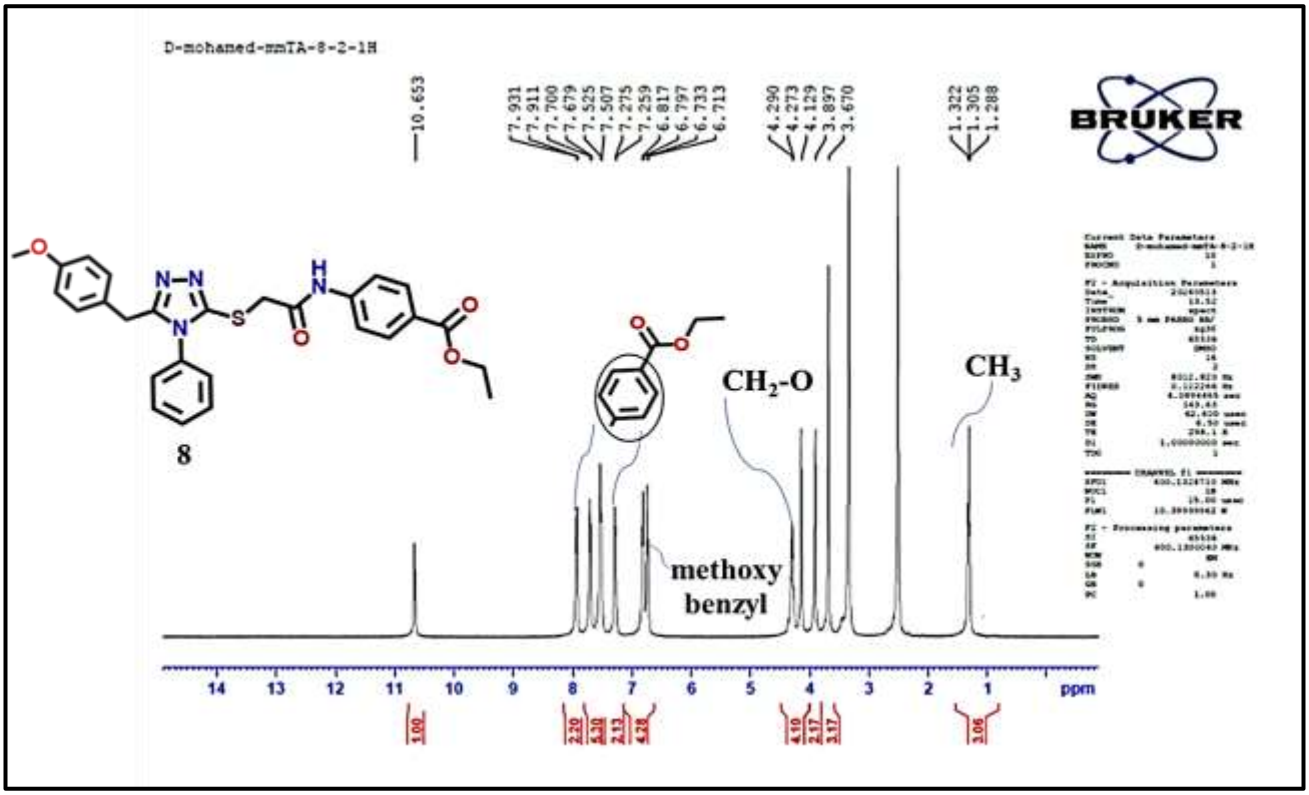

^{13}C NMR spectrum of compound **8**

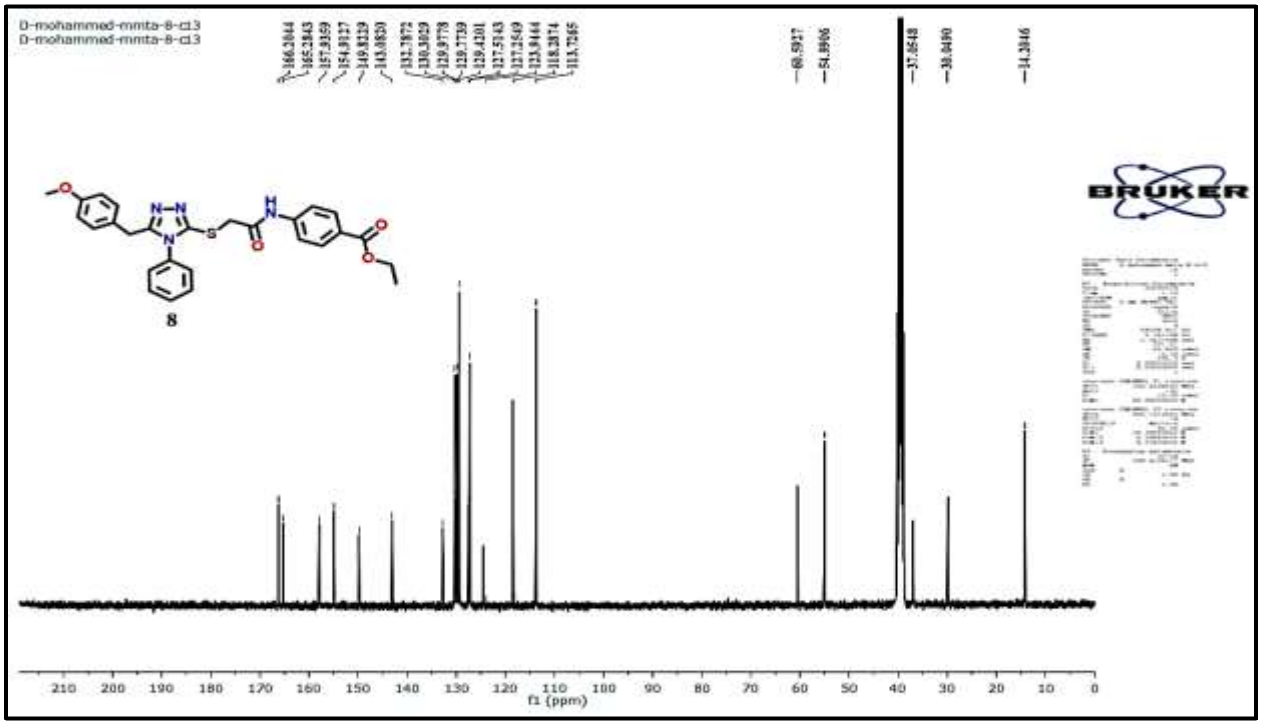

IR-Chart of compound **9**

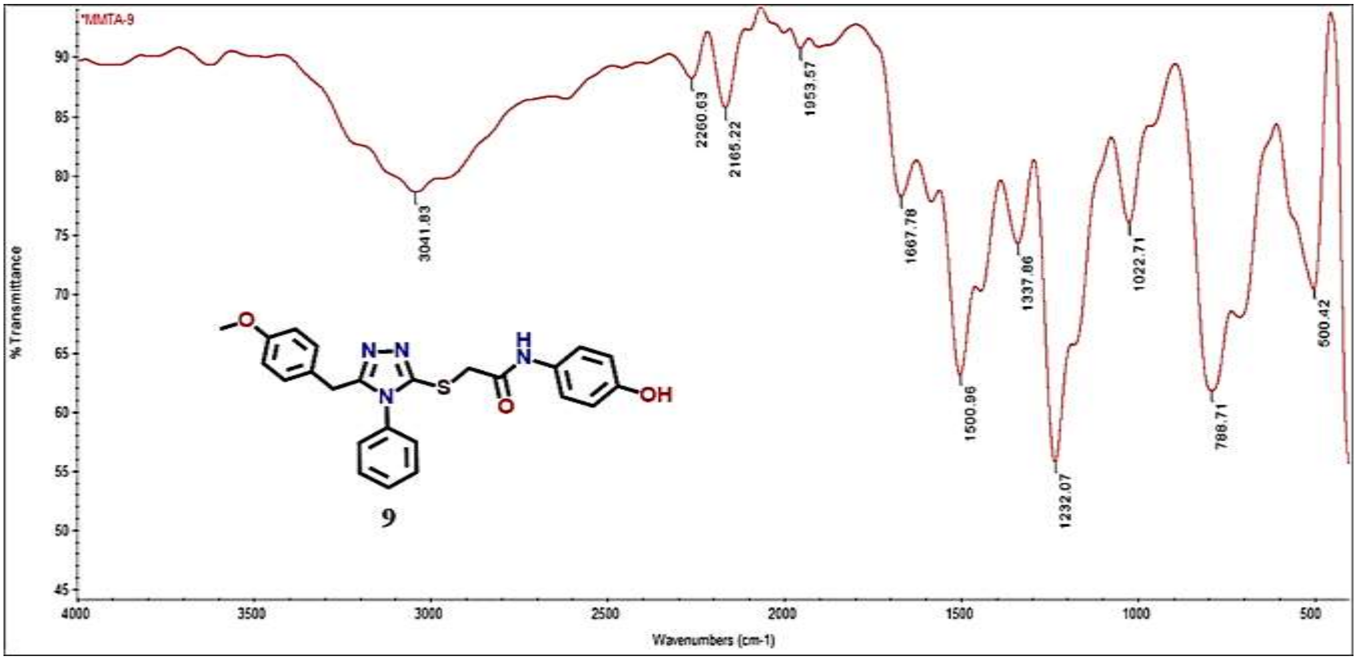

^{1}H NMR spectrum of compound **9**

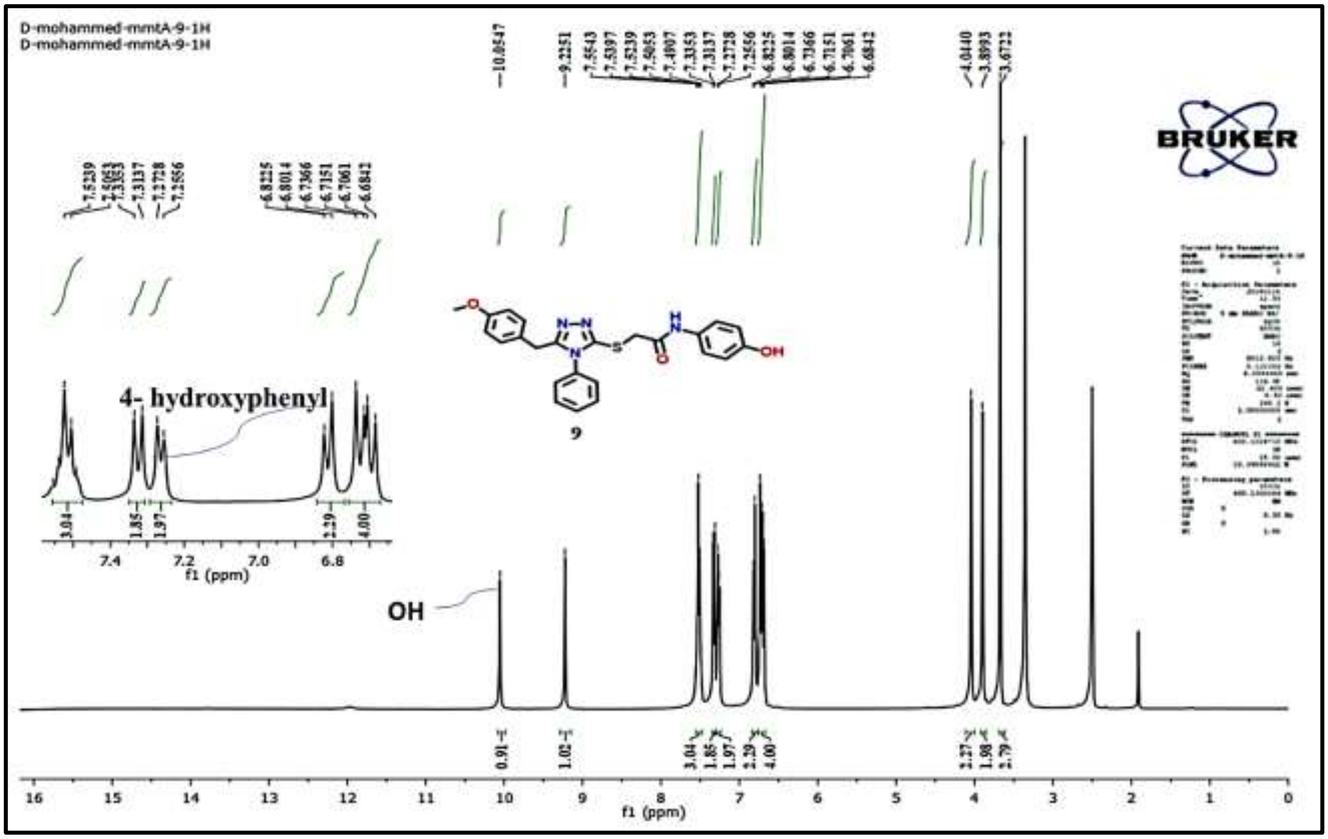

^{13}C NMR spectrum of compound **9**

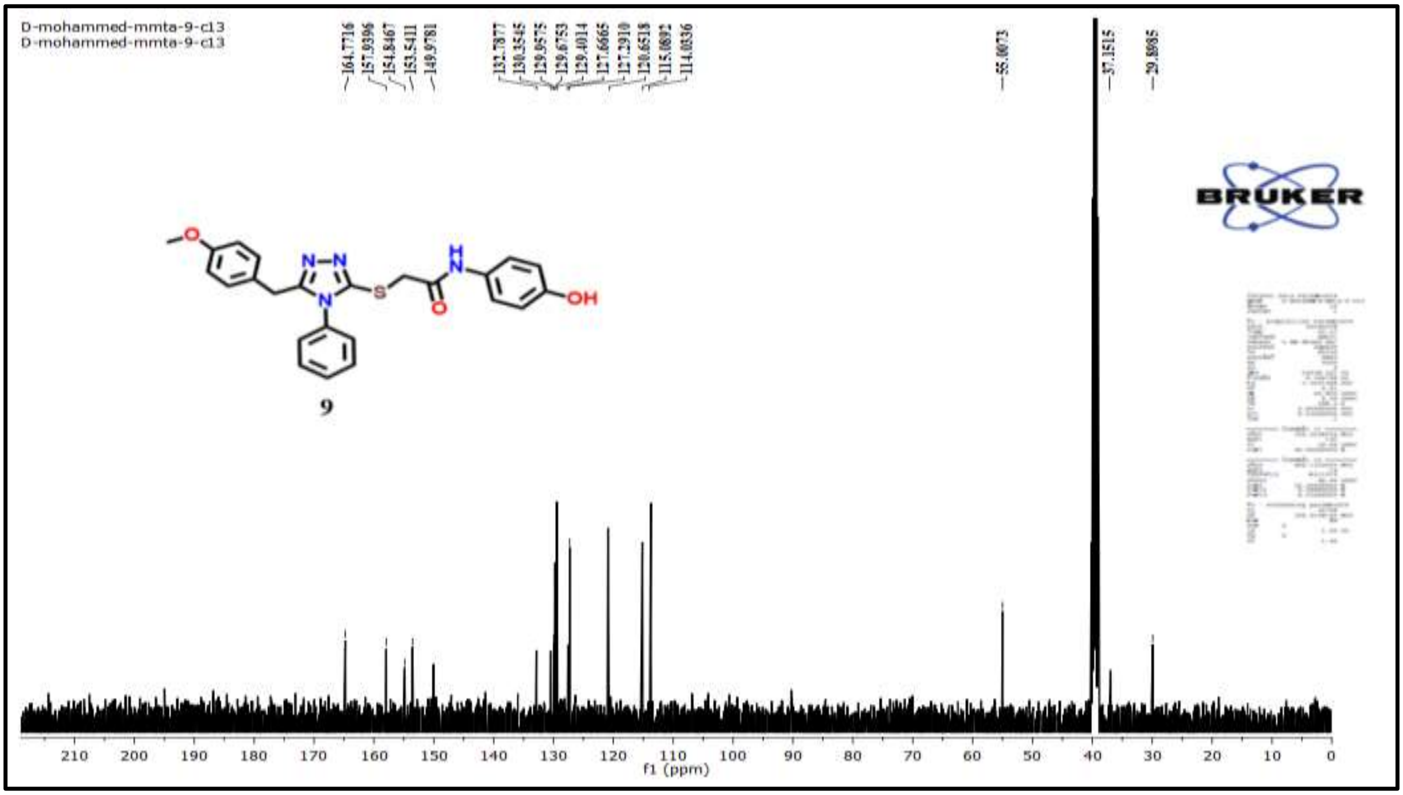

IR-Chart of compound **10**

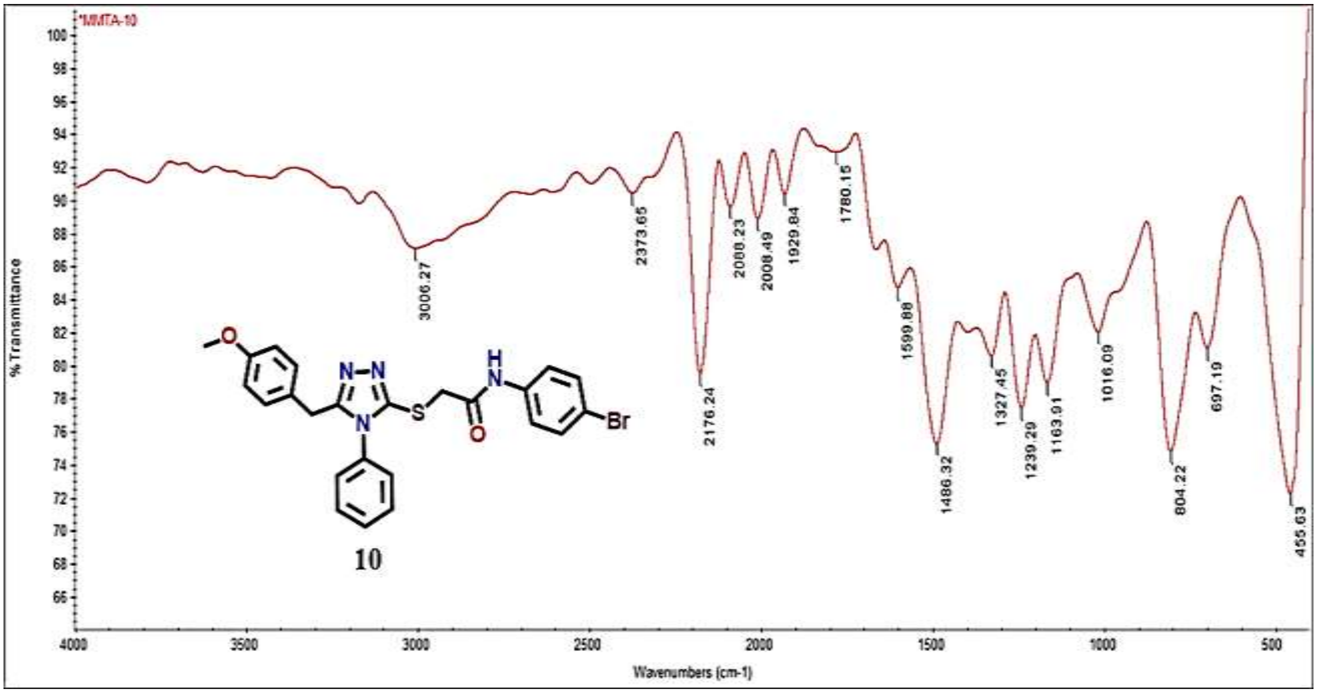

^{1}H NMR spectrum of compound **10**

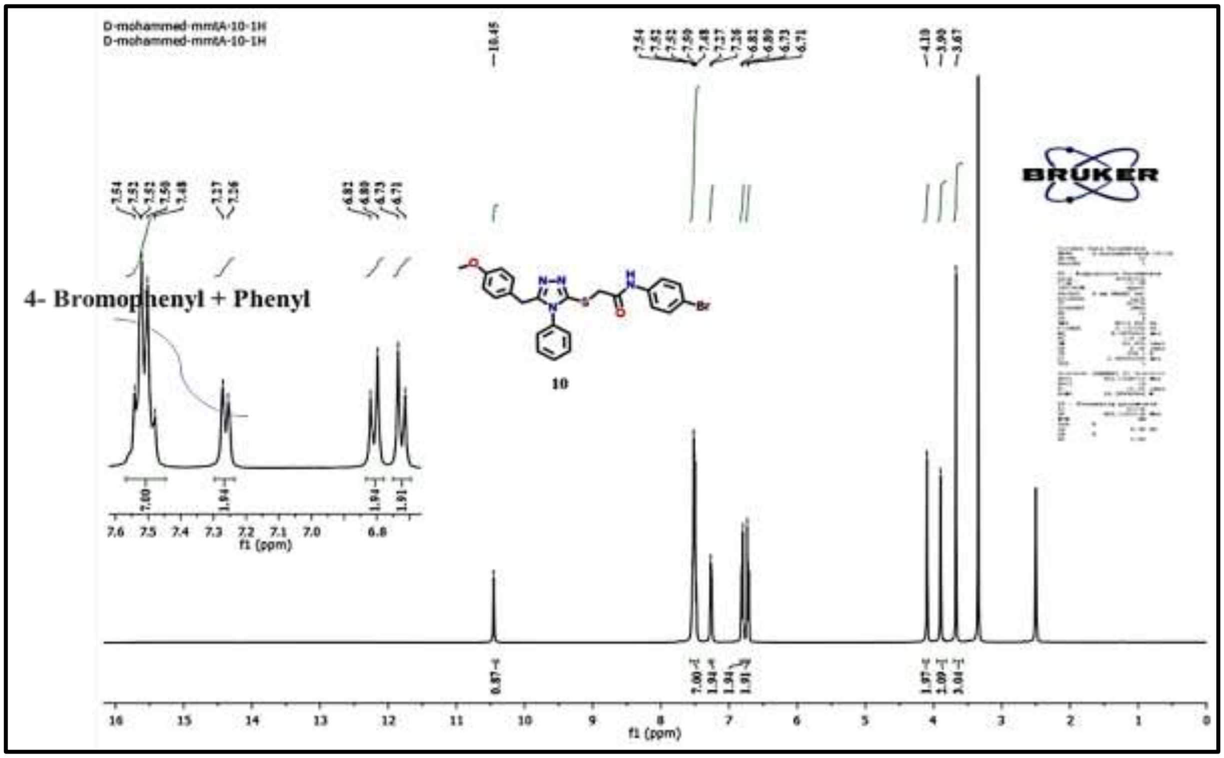

^{13}C NMR spectrum of compound **10**

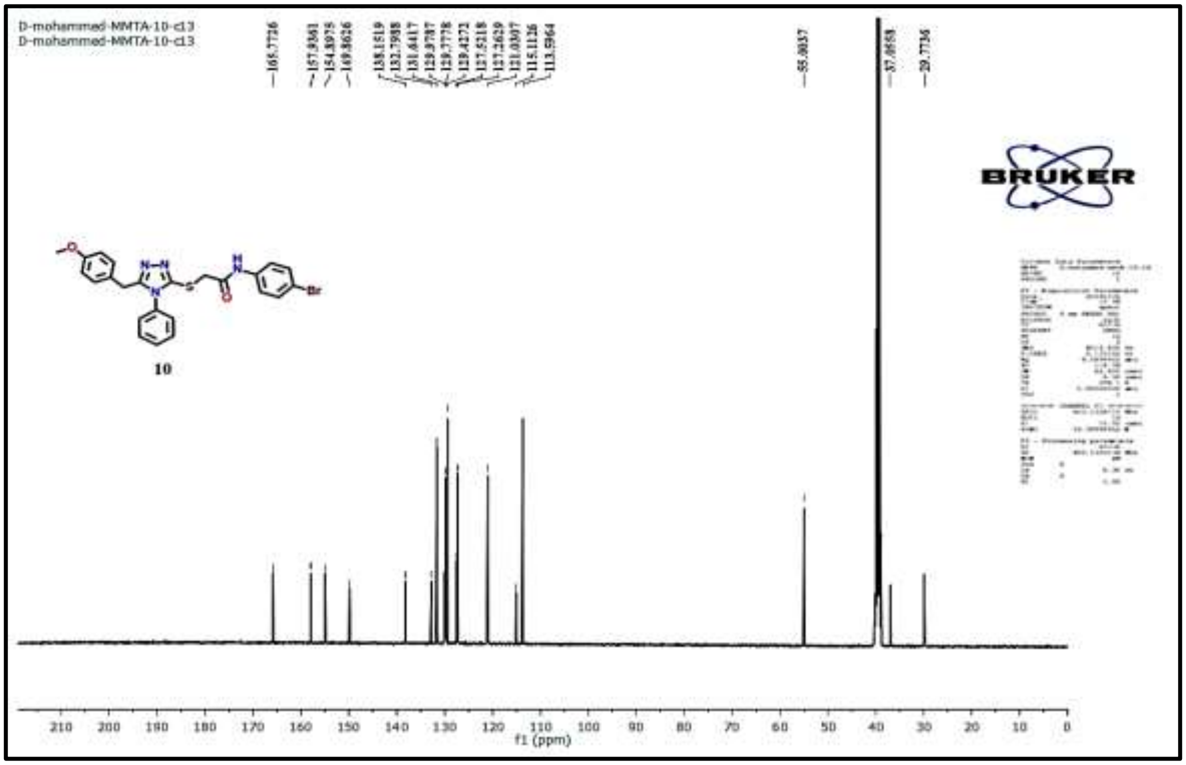

Supplement: Supplementary file 1 — Supplementary Material 1 [file 41598_2025_11214_MOESM1_ESM.pdf]
